# Pore Size Engineering of MOFs by Pore Edge Reaction: Tetrazine Click and Hydrogen Adsorption in Theory and Experiment

**DOI:** 10.1021/acs.chemmater.5c00914

**Published:** 2025-07-02

**Authors:** Damian Jędrzejowski, Michał Ryndak, Gabriela Jajko-Liberka, Paweł Kozyra, Witold Piskorz, Volodymyr Bon, Stefan Kaskel, Dariusz Matoga

**Affiliations:** † Faculty of Chemistry, Jagiellonian University in Krakow, Gronostajowa 2, 30-387 Kraków, Poland; ‡ Doctoral School of Exact and Natural Sciences, Jagiellonian University in Krakow, Łojasiewicza 11, 30-348 Kraków, Poland; § Faculty of Chemistry and Food Chemistry, 9169 Technische Universität Dresden, Bergstraße 66, 01069 Dresden, Germany

## Abstract

Precise control over the porosity of metal–organic frameworks (MOFs) is crucial to optimize their properties and leverage their inherent tunability. However, there are ongoing challenges in pore size engineering for each MOF platform such as preserving crystallinity and morphology and facilitating reliable theoretical predictions throughout a series of modulated structures. Among postsynthetic strategies, mainly covalent functionalization appears to simultaneously preserve structural integrity and enable accurate theoretical predictions. Here, we present a MOF platform [M_2_(RCOO)_4_(H_2_O)_2_], JUK-21­(M), M = Cu or Zn, containing a tetrazine-based tetracarboxylate linker, which we covalently functionalize using the inverse electron-demand Diels–Alder reaction (iEDDA) and five dienophiles of various bulkiness, yielding a series of JUK-21­(Cu)*-x* MOFs. In addition to experiments, the iEDDA reactivity is assessed by applying a charge distribution susceptibility analysis, including Fukui functions, hardness, and relevant donor/acceptor orbitals. Comprehensive theoretical and experimental insights into the adsorption of nitrogen and hydrogen by JUK-21­(Cu)*-x* enable rationalization of the observed isotherms and show the isosteric heat of hydrogen adsorption as a highly sensitive parameter to validate the modification efficiency. Our findings indicate to what extent the pore size of MOFs affects the adsorption properties and highlight potential pitfalls that arise even with the precise covalent functionalization of MOFs.

## Graphical abstract

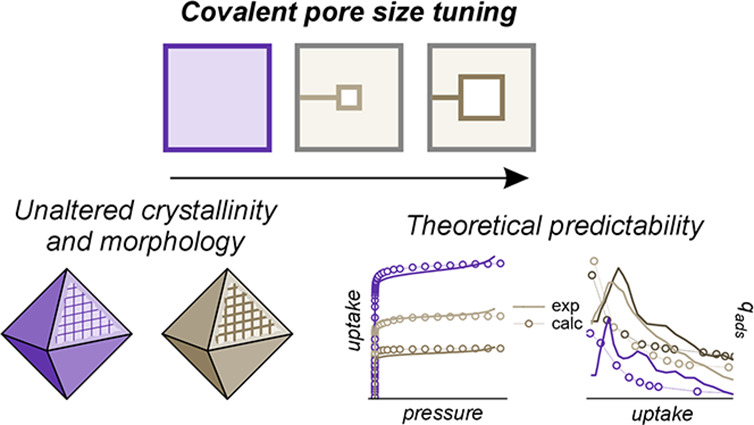

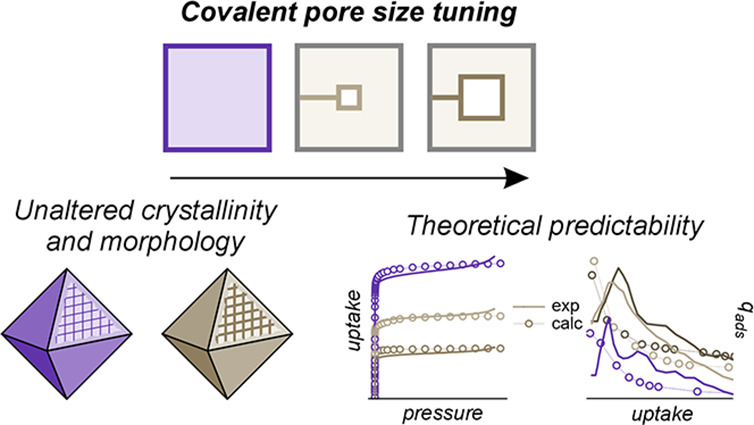

## Introduction

Metal–organic frameworks (MOFs) are a highly versatile class of porous materials, valued for their tunable chemistry, structural diversity, and high surface area, making them essential for applications in adsorption, separation, catalysis, and sensing.^1–6^ A key challenge in MOF functionalization, including pore size engineering, lies in achieving precise chemical functionalization while maintaining structural integrity.^7^ In general, functionalization can be presynthetic (functionalization of building blocks),^8–10^ postsynthetic (functionalization after framework assembly),^11,12^ or a combination of both.^13^ The choice of strategy depends on the feasibility of the synthesis, the stability of the reactants and products, and the application requirements. Furthermore, there are two fundamental constraints that limit the potential of the two strategies: theoretical predictive control over the resulting structures (limited by model accuracy),^14,15^ and experimental control over MOF morphology,^16,17^ which influences the solid-state properties of the material (Figure 1).

**Figure 1:**
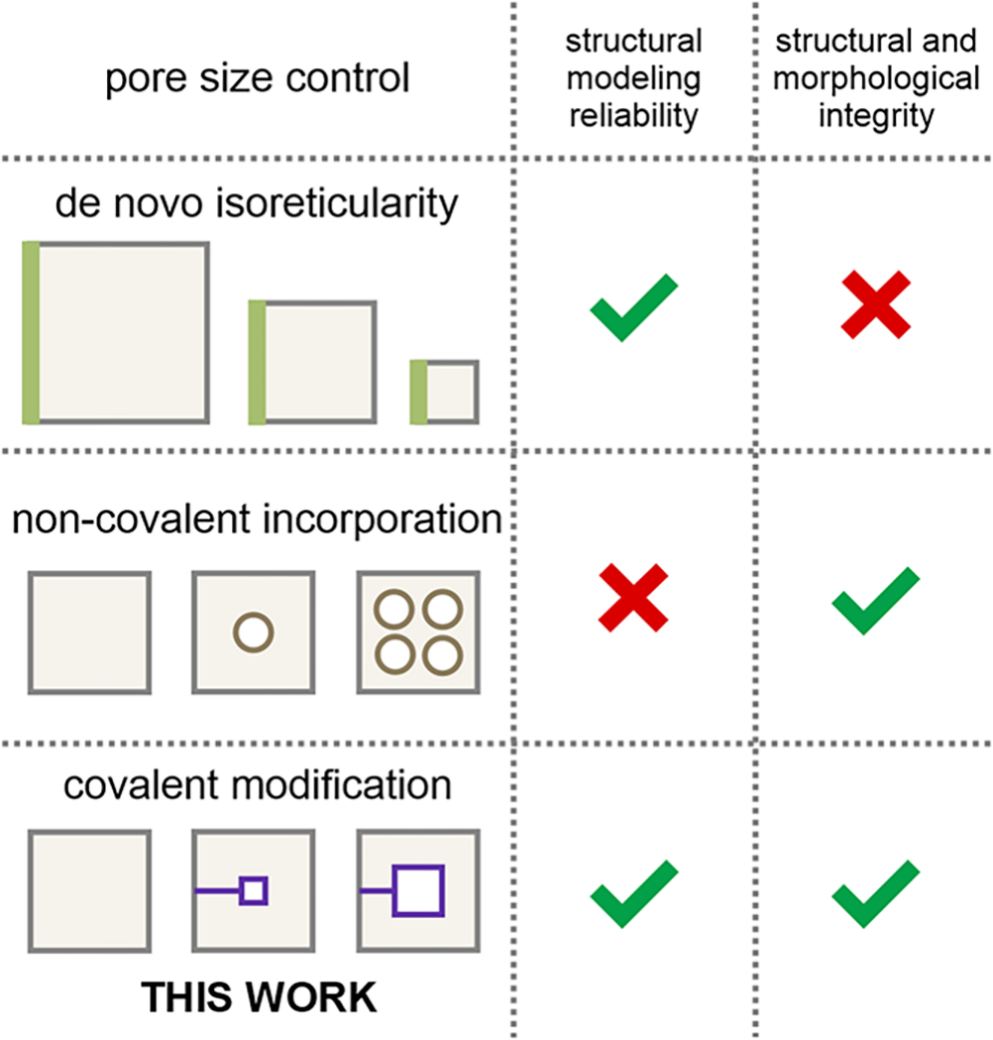
Strategies for pore size engineering of MOFs: de novo isoreticularity, noncovalent incorporation, and covalent modification, highlighting their impact on (i) the reliability of theoretical models, understood as both structural models and models describing material properties, and (ii) structural and morphological integrity, i.e., the ability to retain uniformity in the structural framework, crystallite size and shape, and defect levels across the entire series of modified materials.

In this context, pore size engineering of MOFs can be realized by three strategies: de novo isoreticular design, noncovalent incorporation, and covalent modification (Figure 1). While de novo isoreticular design (also referred to as presynthetic functionalization) allows precise theoretical modeling,^18,19^ it may disrupt topology and morphology in a MOF series or may result in interpenetrated or defective frameworks due to self-assembly constraints.^20,21^ Consequently, achieving two identical macroscopic batches from separate syntheses remains a major challenge.

Noncovalent incorporation includes both the introduction of guest molecules via weak interactions^22,23^ and coordination-based insertions.^24,25^ Such modifications typically maintain morphology, but lack predictability due to weak interactions (disorder, uncertain yield)^26,27^ or coordination bond-related constraints (inserted ligand lability, low functionalization efficiency).^28,29^ Covalent modifications seem to offer the best of both approaches, allowing controlled and periodic functionalization while preserving bulk morphology.^30–32^ Such modifications are performed directly on an existing parent framework whose structural integrity can be maintained, allowing reliable comparisons between different functionalized derivatives. Additionally, the formation of strong covalent bonds provides a well-defined, ordered functionalization, significantly improving theoretical predictability.^33^ However, the success of this strategy depends on high-yield, chemoselective, and irreversible reactions with rapid kinetics and mild conditions to prevent framework degradation.^34^ Ideally, such reactions should also be versatile, allowing for the introduction of various functionalities across different MOF platforms.

These criteria are met by bioorthogonal transformations,^35,36^ such as the inverse electron-demand Diels–Alder (iEDDA) reaction,^37^ which has emerged as a powerful tool for MOF functionalization.^38,39^ Although iEDDA reactions have been widely explored in solution-phase chemistry,^40–42^ their applications to MOFs remain limited to a few studies, including framework rigidification,^43^ luminescent sensing,^44^ conjugation of fullerenes^45^ or carbon nanotubes,^46^ introduction of superhydrophobicity,^47^ and photocatalyst design.^48^ These examples highlight the potential of iEDDA reactions for expanding the chemical versatility of MOFs through tailored postsynthetic transformations. It should be noted that the literature on MOFs lacks systematic research on iEDDA reactivity, its impact on MOF stability, and its theoretical predictability.

Herein, we present JUK-21, a new [M_2_(RCOO)_4_(H_2_O)_2_] (M = Cu^2+^, Zn^2+^) MOF based on a tetracarboxylate tetrazine functionalized ligand, 5,5′-(1,2,4,5-tetrazine-3,6-diyl)­diisophthalate (tztc^4–^), as a platform for covalent postsynthetic modification (CPSM) via the iEDDA reaction. Using a series of dienophiles of various bulkiness, we study the impact on structure and solid-state properties, with parallel experimental–theoretical validation of the sorption capacity and heat of hydrogen adsorption, which serve as a measure of the accuracy of the theoretical model. Through an in-depth theoretical analysis of the reactivity of dienes and dienophiles, we identified strategies to overcome the three main limitations of covalent modifications: low yields, framework destabilization, and formation of defects. Our results demonstrate that the iEDDA reaction enables precise chemical tuning of MOFs while preserving their morphology at both the atomic and crystallite scales, establishing it as a robust strategy for advanced MOF design.

## Results and Discussion

### Synthesis and Characterization of JUK-21­(M)

The inherent symmetry of the *s*-tetrazine ring and the synthetic constraints associated with its preparation have historically limited the design of tetrazine-based ligands for MOFs. To date, only two such ligands—dipyridyl (dpt)^43,44^ and bis­(phenylcarboxyl) (tzdc)^45,47,49^ —have been reported. However, dpt requires mixed-linker MOF construction, while tzdc significantly weakens coordination bonds.^49^ Here, we introduce a novel tetratopic ligand, tztc^4–^ (Figure 2a), offering a straightforward and versatile approach to the development of functional and functionalizable MOFs. The ligand precursor, H_4_tztc, was fully characterized using various physicochemical techniques (Supporting Information (SI), Section S3.1), and its structure was determined by single-crystal X-ray diffraction (Section S4.2).

**Figure 2:**
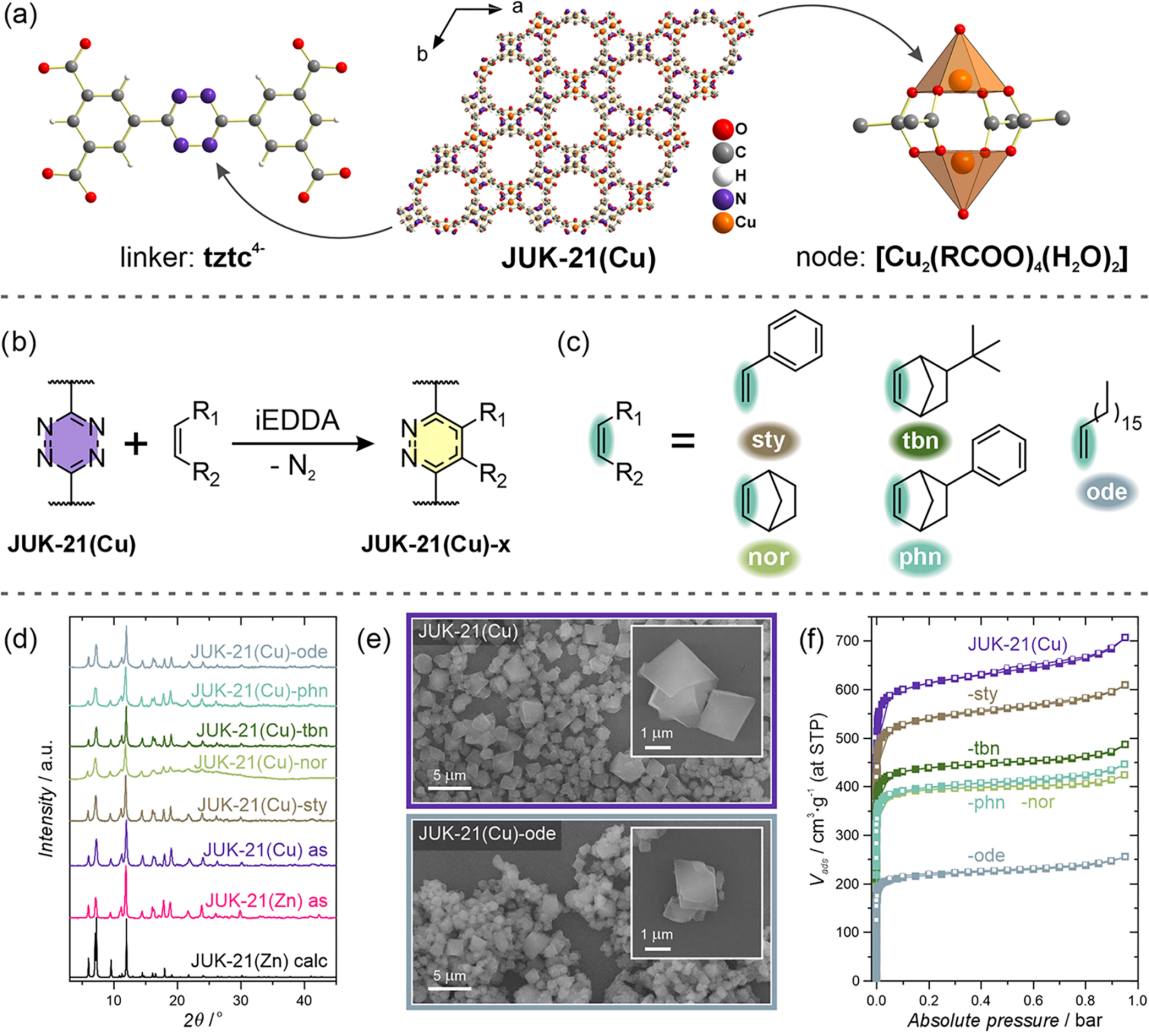
(a) Crystal structure of JUK-21­(Cu), view along [001], secondary building units (SBUs) shown enlarged. (b) Schematic representation of the iEDDA reaction on the tztc^4–^ ligand. (c) Structures of dienophiles used and reactive fragments are highlighted in blue and accompanied by color-coded labels (a color code consistent throughout the work). (d) Powder X-ray diffraction (PXRD) patterns of the JUK-21­(Cu)*-x* series compared with the experimental and calculated patterns of JUK-21­(Zn). (e) Scanning electron microscopy (SEM) images of JUK-21­(Cu) and its reaction product with dienophile *ode* under the harshest conditions (additional images in Section S9). (f) Nitrogen adsorption–desorption isotherms at 77 K of the JUK-21­(Cu)*-x* materials.

Two MOFs, JUK-21­(Zn) and JUK-21­(Cu), were synthesized in the solvothermal reaction between zinc and copper nitrates and the H_4_tztc ligand precursor by using *N*,*N*-dimethylformamide (DMF) as a solvent (Figure 2a). Large crystals (200–400 μm), obtained for JUK-21­(Zn) were ideally suited for single-crystal X-ray diffraction as a structural analysis technique (Section S4.1), while the isostructurality of JUK-21­(Cu), which crystallizes as ∼1 μm particles (Figures 2e and S48), was confirmed by powder X-ray diffraction (PXRD) (Figure 2d; Section S4.3). Both materials crystallize in the trigonal *R*3̅*m* space group and adopt an *nbo* topology, comprising tztc^4–^ linkers and paddlewheel [M_2_(RCOO)_4_(H_2_O)_2_] secondary building units. While this coordination motif is common in copper-based MOFs, it is rarely reported for zinc-based systems.^50,51^ For example, a paddlewheel nontetrazine analogue of JUK-21­(Cu) was reported in the literature as a benchmark material for high H_2_ sorption capacity.^52^ This study focused exclusively on presynthetic pore size control and sorption performance, without theoretical modeling or morphology analysis (cf. Figure 1), both of which are central to our investigation.

Further structural validation for JUK-21­(Cu) was obtained from spectroscopic analysis, with NMR confirming the exclusive presence of ligand-derived peaks (Figure S18) and infrared spectroscopy identifying characteristic ν_C–O,sym_ (1457 cm^–1^) and ν_N(ar)=N(ar)_ (1384 and 1362 cm^–1^) vibrations (Figure S38). The frameworks feature an extensive network of void channels initially filled with solvent molecules. Activation of JUK-21­(Cu) by removal of guest molecules yields a permanently porous material with a high Brunauer–Emmett–Teller (BET) surface area of 2460 m^2^/g and a total pore volume of 1.10 cm^3^/g (Figure 2f; Section S10). Gas adsorption measurements confirm porosity toward both nitrogen and carbon dioxide (Figure S55), with pore size distribution analysis revealing a dominant channel diameter of ∼12 Å.

Thermogravimetric analysis (TGA) indicates a mass loss of ∼49%, corresponding to the removal of 2 H_2_O and 15 MeOH molecules per [Cu_2_(tztc)] unit, and defines a stable activated phase over the 160–265 °C range (Figures S45 and S46). Oxidative conditions revealed missing linker defects at an estimated level of ∼11% (Figure S47 and Table S4).

Due to the unfavorable coordination environment of zinc­(II), JUK-21­(Zn) is neither stable nor porous upon full guest removal. Theoretical calculations further indicate significantly lower reactivity in the iEDDA reaction, which we confirmed experimentally. Consequently, JUK-21­(Zn) serves solely as a structural model subjected to basic characterization, while further studies, including iEDDA modifications and sorption measurements, were conducted exclusively on JUK-21­(Cu).

### iEDDA Postsynthetic Modification of JUK-21­(Cu)

To ensure a strong experimental–theoretical correlation while preserving material morphology, JUK-21­(Cu) was modified using the inverse electron-demand Diels–Alder reaction (iEDDA) (Figure 2c) with a series of dienophiles: styrene (*sty*), 2-norbornene (*nor*), 5-*tert*-butyl-2-norbornene (*tbn*), 5-phenyl-2-norbornene (*phn*), and 1-octadecene (*ode*) (Figure 2d). This series introduces moieties of varying steric bulk, deliberately lacking functional groups that could introduce secondary interactions, ensuring a pure steric pore control effect. Notably, *tbn* and *phn* are used here for the first time in the iEDDA reaction. Unlike guest-responsive and interpenetrated JUK-20,^43,44^ the fully rigid framework of JUK-21­(Cu) allows for precise structural interpretation.

All five dienophiles selectively and rapidly reacted with the tetrazine system under mild conditions (Section S3.3), resulting in modified materials with different colors (Figure S42). The completeness of the reaction was confirmed by ultraviolet–visible–near-infrared spectroscopy (UV–vis–NIR) reflectance spectroscopy (loss of the 542 nm absorption band and appearance of a 352 nm band, Figure S44), infrared spectroscopy (disappearance of the N_ar_=N_ar_ vibration bands, Figures S39 and S40), and NMR spectroscopy of digested and ligand-extracted samples (Section S5.2). The compositions of the materials were further corroborated by elemental analysis (Section S3.3). Although most modifications reached full conversion (100%), JUK-21­(Cu)-*tbn* and JUK-21­(Cu)-*ode* retained residual tetrazine absorbance, with NMR analysis determining conversion rates of 97 and 82%, respectively (Section S5.3).

The characterization of the bulk JUK-21­(Cu)*-x* series was carried out using PXRD (Figures 2d and S5), SEM imaging (Figures 2e and S48–S53), TGA (Figures S45–S47), and N_2_ adsorption studies (Figures 2f and S57). All materials retained crystallinity and are isostructural with the parent framework, except JUK-21­(Cu)-*nor*, which showed partial amorphization. Its reduced stability was further supported by thermal activation temperature analysis (Figure S56) and morphological characterization, which revealed a significant reduction in the average crystallite size to 0.56 μm, while all other samples retained similar dimensions and an intact morphology (Figure S54). The impact of vacuum desolvation temperature on porosity retention was studied to further assess thermal robustness. For JUK-21­(Cu)-*tbn*, -*phn*, and -*ode*, full desolvation without signs of decomposition was achieved within 25–120 °C. On the contrary, JUK-21­(Cu)-*sty* showed early signs of degradation at lower temperatures, while JUK-21­(Cu)-*nor* exhibited partial degradation even after room temperature activation (Figure S56). These results reflect notable differences in the thermal resilience across the series. Thermogravimetric analysis performed under nitrogen (Figure S45) and in synthetic air (Figure S46) further supported this trend. The temperature at which the maximum decomposition rate occurs (dTGA minimum) decreased from 303 °C for the parent JUK-21­(Cu) to 230–280 °C for modified derivatives. Although all materials exhibit initial mass loss between 50 and 180 °C due to solvent removal, postsynthetic modification leads to a gradual reduction in thermal stability, more pronounced under oxidative conditions.

Quantitative defect analysis based on thermogravimetric data revealed that JUK-21­(Cu)-*sty* exhibited a notably higher fraction of missing linkers (∼25%), whereas the other materials maintained the defect concentration of defects at 8–12%, consistent with the parent MOF (Table S4). The calculation procedure is detailed in Section S8, which confirms that the defect content remains essentially unchanged after the postsynthetic modification, except for JUK-21­(Cu)-*sty*. Nitrogen adsorption isotherms aligned well with theoretical predictions (with the only exception of JUK-21­(Cu)-*nor*, Figures S83 and S84), preserving the shape of the type I isotherm and confirming a gradual reduction in specific surface area (*S*_BET_), pore volume (*V*_pore_), and average pore size (*d*_pore_) (Figures 2f and S57; Tables 1 and S5).

**Table 1:** BET Parameters and Sorption Data for JUK-21­(Cu)*-x* Materials^a^

sample	*V*_max_ (cm^3^/g)^b^	*S*_BET_ (m^2^/g)	*V*_pore_^exp^ (cm^3^/g)^b^	*d*_pore_ (Å)	*V*_pore_^calc^ (cm^3^/g)
JUK-21(Cu)	707	2460	1.10	1.19	1.11
JUK-21(Cu)-*sty*	608	2160	0.94	1.11	0.82
JUK-21(Cu)-*tbn*	486	1770	0.75	1.06	0.70
JUK-21(Cu)-*phn*	446	1610	0.69	1.04	0.65
JUK-21(Cu)-*nor*	424	1600	0.66	1.07	0.84
JUK-21(Cu)-*ode*	226	866	0.40	1.11	0.40

a Details are in the Supporting Information, Section S10.

b At *p*/*p*_0_ = 0.95.

Overall, the JUK-21­(Cu)-*x* series exhibits preserved crystallinity, morphology, and well-defined bulky functional groups. The deviations observed for JUK-21­(Cu)-*nor* and JUK-21­(Cu)-*sty* provide valuable insight into structure–property relationships and are discussed further in the following sections. All materials remain chemically stable upon exposure to air and common organic solvents such as methanol, acetone, or toluene, provided they are not vacuum-activated. After activation, the materials must be handled under an inert atmosphere because of the susceptibility of the Cu paddlewheel node to hydrolysis.

### JUK-21­(Cu)-*x* Structural Analysis

Due to the micrometer-sized crystallites of JUK-21­(Cu)-*x*, single-crystal X-ray diffraction could not be used to resolve their structures. The nonreactivity of JUK-21­(Zn) in the iEDDA reaction prevented isostructural validation as was done for JUK-20­(Zn).^44^ Instead, molecular evidence of reaction completion and macroscopic techniques confirmed structural preservation. A theoretical model was built using JUK-21­(Zn) as a starting point, substituting Zn with Cu and modifying the ligand accordingly. Geometry optimizations were performed at the periodic DFT level of theory with use of VASP^53,54^ (SI, Section S4.4). For structural analysis, axial water molecules on Cu were retained, while for sorption modeling, they were removed, which corresponds to activation before adsorption. To explore the potential energy surface in a broad way, the model was simplified to a primitive P1 unit cell. Figure 3 illustrates the optimized asymmetric unit and framework packing along [001], with additional projections provided in Figures S6–S11. Each iEDDA-functionalized group fits into the pores, leaving a free space.

**Figure 3:**
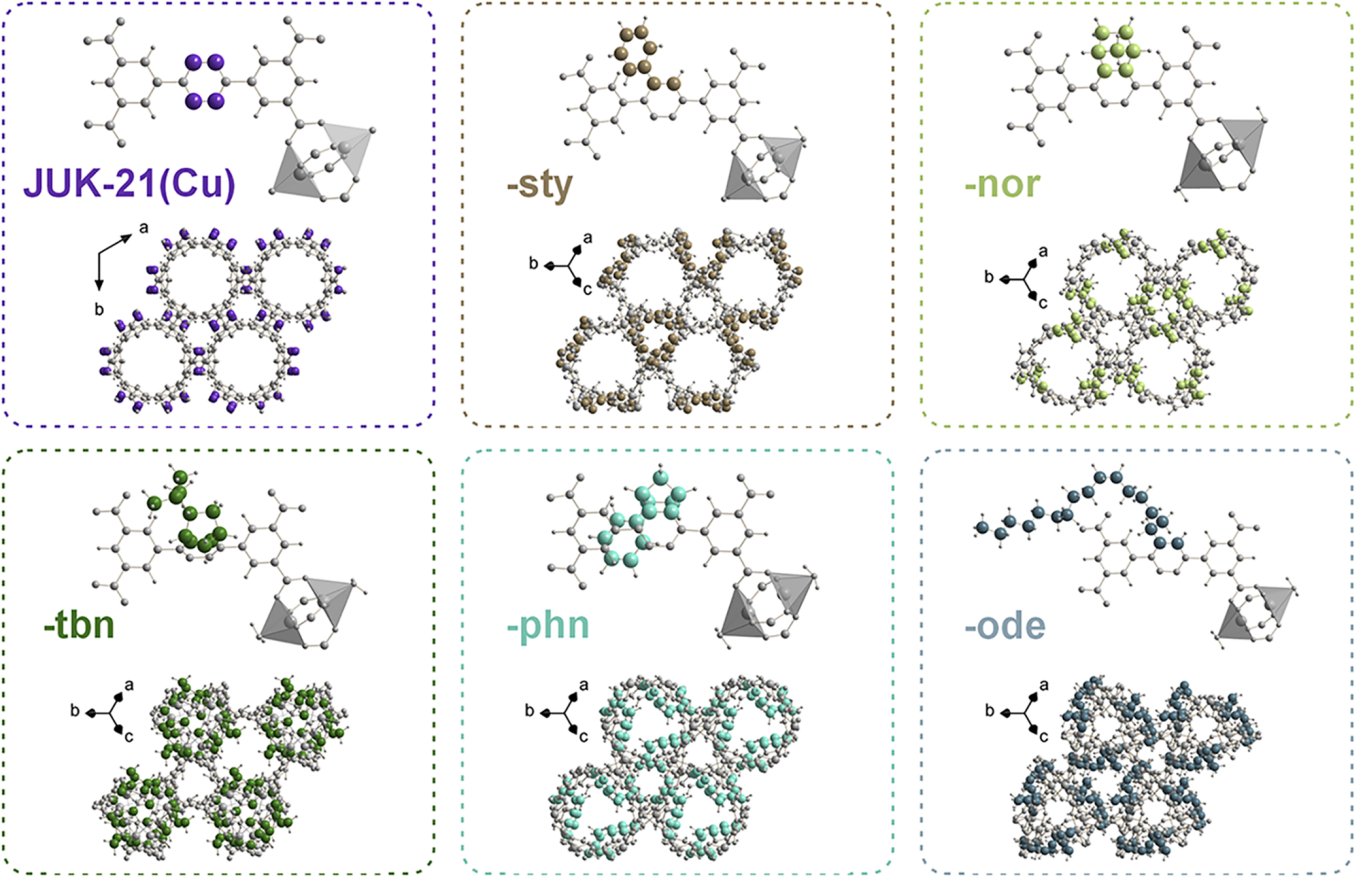
Basic building unit of each representative in the JUK-21­(Cu)-*x* series showing the ligand and coordination environment. Structure projections along the [001] direction ([111] for the triclinic system) illustrate the primary free-space channels and their progressive reduction in size upon modification. Alternative projections can be found in SI, Figures S6–S11.

The optimized ligand geometries in JUK-21­(Cu)-*x* reveal structural distortions that affect the stability of the materials (SI, Section S4.5). Three deviations from the initial *nbo* topology of the parent JUK-21­(Cu) were analyzed: inner-ring torsion, outer-ring twisting, and linker bending (Figures S12 and S13). The most pronounced inner-ring torsion occurs in JUK-21­(Cu)-*tbn* (68°), followed by -*phn* (44°) and -*ode* (37°). The twisting of the outer rings is greatest for -*tbn*, -*ode*, and -*sty*, while JUK-21­(Cu)-*nor* shows no twist due to its symmetric nature. However, JUK-21­(Cu)-*nor* exhibits the highest linker bending (159°), introducing an internal stress that elongates coordination bonds and leads to partial degradation. JUK-21­(Cu)-*sty* shows a smaller bending angle (163°), but, combined with significant twisting, undergoes partial ligand decoordination, increasing the materials defect concentration. In JUK-21­(Cu)-*tbn*, -*phn*, and -*ode*, the central ring torsion is counteracted by minimal bending (170–175°), with JUK-21­(Cu)-*phn* further stabilized by π–π interactions (Figure S14). This structural analysis accounts for the higher defectivity and reduced stability of JUK-21­(Cu)-*sty*, in line with the TGA-based findings, as well as for the instability of JUK-21­(Cu)-*nor* and the enhanced robustness of the other materials.

### Theory on iEDDA Reactivity

Theoretical analysis of dienes and dienophiles is essential for rational material selection for iEDDA modification. Martí-Gastaldo and co-workers demonstrated that incorporating the tzdc^2–^ ligand into a MOF lowers the lowest unoccupied molecular orbital (LUMO) energy by 3.8 kJ/mol, improving reactivity compared to the free ligand.^45^

Here, we analyzed three dienes: H_4_tztc, JUK-21­(Zn), and JUK-21­(Cu), along with five dienophiles. To compare their highest occupied molecular orbital (HOMO) (dienophiles) and LUMO (dienes) energies, we employed absolute hardness (η),^55^ equal to the midpoint of the HOMO–LUMO gap (Figure 4a).^56^ Each level was verified using Fukui function analysis with representative contours shown in Figure 4b (full data in Figures S70 and S71). Computational details are provided in the Supporting Information (Section S13). JUK-21­(Zn) shows only a slight decrease in LUMO energy (0.041 eV, ∼3.9 kJ/mol), similar to UiO-68-tzdc,^45^ whereas JUK-21­(Cu) shows a substantial reduction (0.206 eV, ∼20 kJ/mol) compared to H_4_tztc.

**Figure 4:**
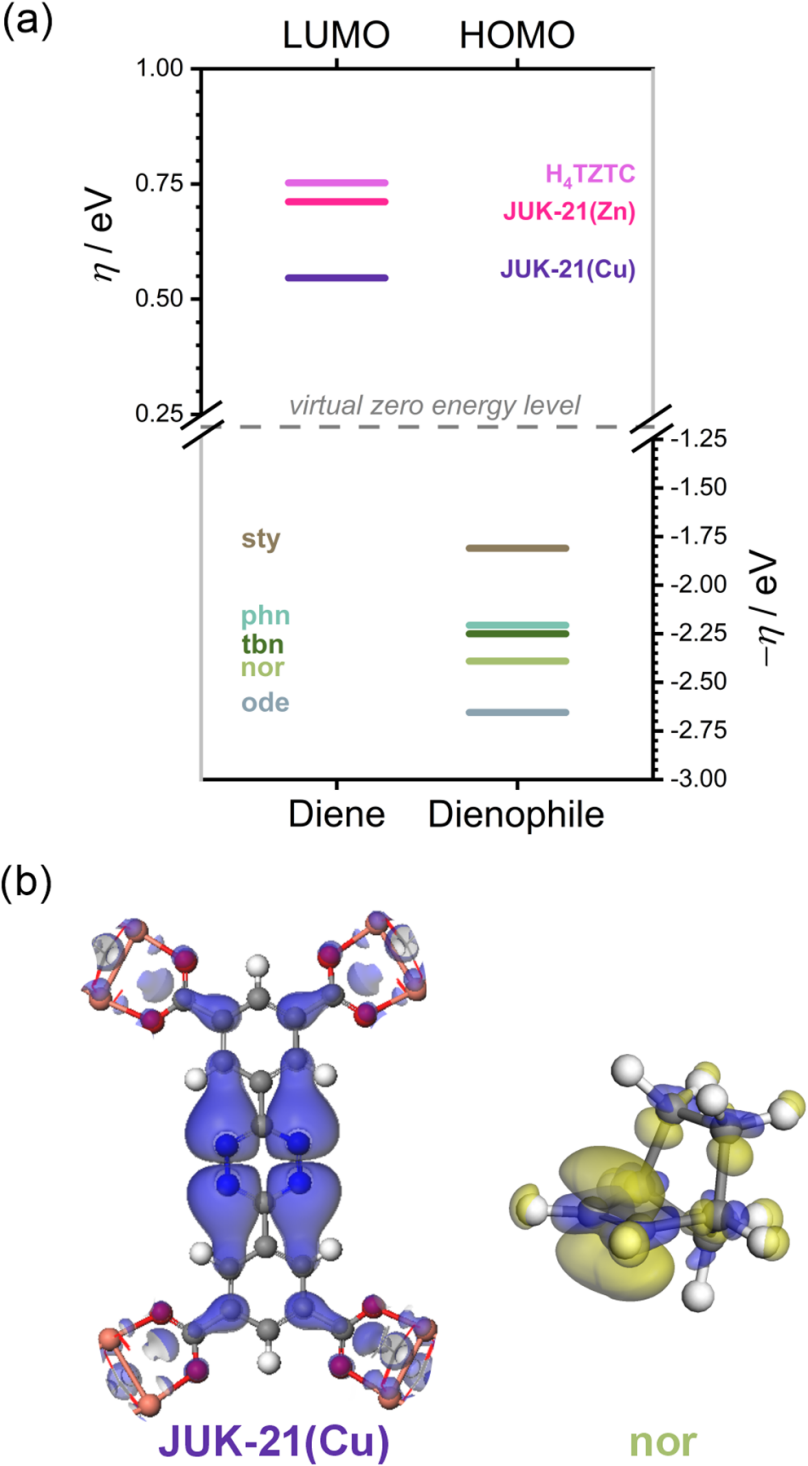
(a) Diagram of LUMO energy levels for dienes (H_4_tztc, JUK-21­(Zn)), LUMO + 3 for JUK-21­(Cu) and HOMO levels for dienophiles (*sty*, *nor*, *tbn*, *phn*, *ode*), defined by absolute hardness (η). The dashed line marks the virtual zero reference level. (b) Isosurfaces of the electrophilic (blue) Fukui function for JUK-21­(Cu) and the nucleophilic (yellow) Fukui function for *nor*, illustrating the spatial distribution of acceptor and donor reactivity indicators, respectively.

This drastic decrease in the HOMO–LUMO gap explains the high reactivity of JUK-21­(Cu) and the inertness of JUK-21­(Zn). Additional factors, such as larger JUK-21­(Zn) crystallites that cause diffusion limitations and lower structural stability, further hinder its reactivity. The enhanced reactivity of JUK-21­(Cu) arises from both the Cu­(II) coordination environment and the solid-state band structure.

Dienophile reactivity also depends on the electronic structure, angular strain, and diffusion effects (summarized in Table 2). The HOMO energy difference between the apparently most reactive *sty* and the least reactive *ode* is 0.844 eV (∼81 kJ/mol), strongly determines its reactivity (see Table S1). However, *nor* is experimentally the most reactive due to angular strain, enhanced diffusion, and material degradation that improves crystallite access. *Phn* reacts faster than *tbn* but slower than *sty*, benefiting from the angular strain and π-π stabilization. *Tbn* exhibits a reduced reactivity because of diffusion constraints and significant ligand rearrangement.

**Table 2:** Theoretically Calculated and Structural Parameters of Dienophiles and Products of Their Reaction with JUK-21­(Cu), Affecting the Rate of the iEDDA Reaction

*x* ^a^	η_Cu_ + η*_x_* (eV)^b^	angular strain	molecular size (Å)^c^	π–π stabilization	material degradation
*nor*	2.94	+	4.3	–	+
*sty*	2.36	–	6.2	–	±^d^
*phn*	2.75	+	8.3	+	–
*tbn*	2.80	+	6.3	–	–
*ode*	3.20	–	17.3	–	–

a Dienophiles arranged in order of iEDDA reaction rate (Table S1).

b Sum of absolute hardness of JUK-21­(Cu) and dienophile (Tables S5 and S6).

c Estimated largest molecule size (based on JUK-21­(Cu)-*x* crystal structure).

d Formation of defects.

These findings underscore the crucial role of the electronic structure in iEDDA reactivity. The choice of metal must consider not only coordination bond stability but also LUMO energy modulation, favoring Cu­(II) for stable and reactive tetrazine MOFs. Dienophile selection should account for electronic effects, although angular strain and molecular size adjustments can partially compensate for unfavorable electronic effects.

### Hydrogen Adsorption—Experimental and Theoretical Studies

To evaluate the effect of pore size on hydrogen sorption capacity, we conducted the series of hydrogen physisorption experiments and calculated heat of adsorption in a structurally consistent JUK-21­(Cu)-*x* series. While nitrogen adsorption provides basic porosity data, hydrogen is a more application-relevant probe, crucial for energy storage materials. The heat of adsorption of hydrogen depends on the concentration of open metal sites (OMS) and the diameter of the pores, while the contribution of other framework functional groups is typically marginal.^57–59^ Our materials feature a constant OMS concentration across the series and different pore sizes as a result of the IEDDA modifications. To isolate this effect, we deliberately employed dienophiles bearing no functional groups capable of strong specific interactions such as hydrogen bonding. This ensured that the observed variations in adsorption enthalpy result exclusively from steric pore modulation without interference from the chemical functionalities. With a well-defined crystallite morphology and a reliable structural model, we can, for the first time, quantitatively assess the impact of the pore size on the hydrogen heat of adsorption.

JUK-21­(Cu) adsorbs more than 50 mg/g of hydrogen at 100 bar (77 K, Figures S58–S61) and shows a minor isotope effect at low pressures (57–77 K), with deuterium uptake (Figures S62 and S63) and heat of adsorption (Figure S64) slightly exceeding that of hydrogen. These trends, typical of porous materials containing OMS, establish a reference for modified structures.^60–62^

Hydrogen adsorption in the JUK-21­(Cu)-*x* series was analyzed experimentally and by grand canonical Monte Carlo (GCMC) simulations (Figures 5a and S72, details in Section S14.1). Four of six materials aligned well with the theoretical model, including JUK-21­(Cu)-*ode*, which, despite lower conversion, behaved predictably. Significant deviations were observed for JUK-21­(Cu)-*nor*, due to its instability, and for JUK-21­(Cu)-*sty*, whose high defectivity—resulting from both structural torsion-induced linker loss and brief air exposure prior to activation, led to partial degradation. In contrast, the consistent defect level across the remaining materials supports their structural integrity and validates the accuracy of hydrogen uptake predictions.

**Figure 5:**
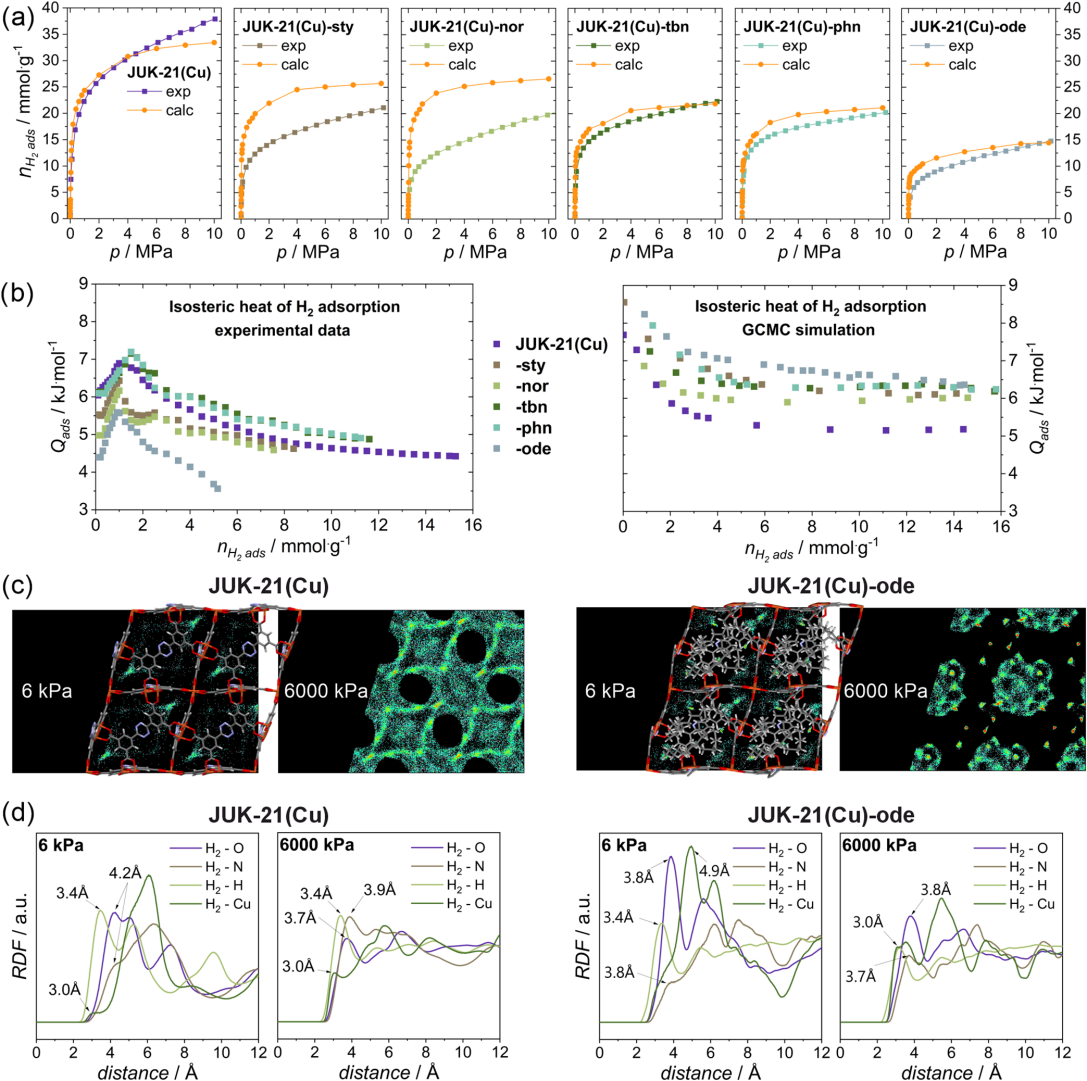
(a) High-pressure hydrogen adsorption isotherms (desorption omitted for clarity) obtained experimentally at 77 K (exp, total adsorption, Figure S60) and via GCMC simulations (calculated) for the JUK-21­(Cu)-*x* series. (b) Comparison of isosteric heat of hydrogen adsorption for each material, determined experimentally (left) and theoretically (right). Average occupation profiles (c) projected along [100] (triclinic cell) and radial distribution function (RDF) (d) for H_2_ adsorption in JUK-21­(Cu) and -*ode* at 6 and 6000 kPa.

The isosteric heat of adsorption was determined by using the Clausius–Clapeyron equation (Section S12.3) and compared with the GCMC simulation and the Widom particle insertion method (Figure 5b; Section S14.6). JUK-21­(Cu)-*tbn* and -*phn* showed a 10–15% increase in the heat of adsorption (for loading from 2 to 12 mmol_H_2__/g), as predicted. On the contrary, JUK-21­(Cu)-*sty* and -*nor* exhibited a slight (∼5%) decrease, despite the model predicting an increase, likely due to defect-related effects outweighing the reduction in pore size. JUK-21­(Cu)-*ode* displayed the largest deviation, with a 30% lower heat of adsorption (3.5 kJ/mol vs the expected maximum), attributed to the flexible hexadecyl side chain. Its conformational lability sterically shields the OMS and disrupts framework–H_2_ interactions, replacing them with weaker H_2_–H interactions. This discrepancy arises because the theoretical model assumes a rigid side group, while in reality, its flexibility significantly alters the adsorption behavior.

The discrepancies between theoretical prediction and experimental results underscore a key observation: while reducing the pore size theoretically increases the adsorption energy, even minor structural changes can dominate in real materials. The heat of adsorption of hydrogen proves to be a highly sensitive parameter, revealing that factors such as reduced crystallite stability (*nor*) or increased defectivity (*sty*) can outweigh the expected pore size effect. This highlights not only the importance of precise MOF design for targeted adsorption properties but also the unique value of the H_2_ heat of adsorption as a precise research tool for evaluating subtle structural modifications.

To refine the adsorption mechanisms, we analyzed average occupation profiles (Figure 5c, complete set in Figure S73) and radial distribution functions (Figure 5d; Section S14.5). At low pressure, the adsorbate molecules preferentially occupy the edges of the framework near the paddlewheel units, while at high pressure, JUK-21­(Cu) utilizes its full volume, still showing the highest concentration around the Cu atoms. A more localized, selective adsorption is discerned for the modified materials, probably because of the more limited access to copper atoms caused by the bulky linkers. This is particularly visible for the JUK-21­(Cu)-*ode*, where access to the metal centers is particularly difficult. This behavior was also confirmed by analyzing the radial distribution functions. Cu–H_2_ interactions (3.0 Å) dominate in JUK-21­(Cu), but for bulkier dienophiles, the distance increases, shifting dominance to H–H_2_ interactions (3.0–3.4 Å), particularly in JUK-21­(Cu)-*ode*, where Cu–H_2_ contacts are nearly lost.

Our study underscores that while nitrogen sorption correlates well with theory, hydrogen adsorption, being more sensitive, reveals fine structural modifications. Our findings highlight key factors for the selection of MOFs and dienophiles in iEDDA-based modifications, ensuring retention of solid-state properties while tuning adsorption characteristics. To achieve this, we employed a structurally rigid MOF platform and controlled all of the structural and morphological parameters, varying only the steric environment of the pores. This strategy allowed us to systematically probe the isolated effect of the pore size on gas sorption properties.

## Conclusions

This work serves as a proof of concept for iEDDA-based covalent postsynthetic modification (CPSM) in MOFs, demonstrating its potential for precise pore size tuning while maintaining theoretical predictability as well as structural and morphological integrity. Using a series of dienophiles for covalent functionalization of JUK-21­(Cu), we established its impact on the adsorption properties while preserving a consistent MOF structure. Our findings define the extent to which reduction of pore size influences hydrogen adsorption enthalpy and highlight key challenges of CPSM, including structural destabilization, defect formation, and incomplete conversion. Crucially, we outline design principles for MOFs and dienophiles to mitigate these unfavorable scenarios, ensuring a controlled functionalization without compromising crystallinity. Beyond validating iEDDA as a selective CPSM strategy, this study underscores hydrogen adsorption as a highly sensitive tool for evaluating the modification efficiency. Our observations are useful in designing MOFs with targeted adsorption properties, including those that may have applications in gas storage and separation technologies.

## Experimental Section

### Synthesis of MOFs and Precursors

All reagents and solvents used for the syntheses of ligands, dienophiles, and MOFs (unless otherwise noted) were commercially available and used without additional purification (Section S1). *Phn* and *tbn* dienophiles were synthesized using optimized literature protocols^63,64^ (see SI, Section S3). The protocols developed for H_4_tztc, JUK-21­(Zn), and JUK-21­(Cu) syntheses are described in detail in the SI (Sections S3.1 and S3.2). Covalent modifications of JUK-21­(Cu) via the inverse electron-demand Diels–Alder (iEDDA) reaction are described in detail in the SI (Section S3.3).

### Physicochemical Characterization

Details of the instrumentation used in this work are provided in the SI (Section S2). The characterization of the synthesized precursors was performed by nuclear magnetic resonance (^1^H, ^13^C NMR, Section S5) and infrared vibrational spectroscopy (IR, Section S6). The physical characterization of the MOFs was performed by powder X-ray diffraction (PXRD, Section S4), CHNS elemental composition analysis (Section S3.3, Table S4), ^1^H NMR after digestion in a mixture of dimethyl sulfoxide (DMSO)-*d*_6_/D_2_SO_4_ and after extraction of the ligand from the digested MOFs (Section S5), IR spectroscopy (Section S6), electron spectroscopy in reflectance mode (UV–vis–NIR, Section S7), thermogravimetric analysis (TGA, Section S8), scanning electron microscopy (SEM, Section S9), and volumetric adsorption analysis of nitrogen and carbon dioxide (at 77 and 195 K, Section S10). Further characterization includes high-pressure hydrogen and low-pressure hydrogen and deuterium volumetric adsorption analysis (at 57–77 K) (Sections S11 and S12).

Details on the solution and refinement of the crystal structures of the H_4_tztc ligand and the material JUK-21­(Zn) are provided in the SI (Section S4). The deposition numbers 2390929 (for H_4_tztc) and 2390930 (for JUK-21­(Zn)) contain the supplementary crystallographic data for this paper. These data can be obtained free of charge from the Cambridge Crystallographic Data Centre www.ccdc.cam.ac.uk.data_request/cif.

### Crystal Structures Modeling and GCMC Simulations

All quantum-chemical calculations of geometries, energies, and electron properties, including orbital energy, orbital contours, and Fukui functions, were performed at the periodic DFT level of theory with use of VASP.^53,54^ The details are described in SI, Section S4.4.

Theoretical studies on the iEDDA reaction, including absolute hardness calculations and calculations of Fukui functions, are provided in SI, Section S13.

Theoretical N_2_ and H_2_ sorption isotherms (at 77 K), average occupation profiles, radial distribution functions, hydrogen heat of adsorption, and pore size distribution in JUK-21­(Cu)-*x* series were calculated using the grand canonical Monte Carlo (GCMC) method, with RASPA code.^65,66^ Details of the models and force fields used are described in the SI (Section S14).

## Supplementary Material

























## Data Availability

Replication data deposited in the Repository of Open Research Data at https://uj.rodbuk.pl/ (10.57903/UJ/VURXSV).

## Notes

All necessary information on the materials, methods of synthesizing ligands and MOFs, modification methods, and descriptions of the physicochemical examination techniques used (PDF)
CIF file for H_4_tztc crystal structure (CIF)
CIF file for JUK-21­(Zn) crystal structure (CIF)
CIF file for JUK_21­(Cu) modeled crystal structure (CIF)
CIF file for JUK_21­(Cu)-*nor* modeled crystal structure (CIF)
CIF file for JUK_21­(Cu)-*ode* modeled crystal structure (CIF)
CIF file for JUK_21­(Cu)-*phn* modeled crystal structure (CIF)
CIF file for JUK_21­(Cu)-*sty* modeled crystal structure (CIF)
CIF file for JUK_21­(Cu)-*tbn* modeled crystal structure (CIF)
AIF files for H_2_ and D_2_ low-pressure sorption on JUK-21­(Cu)-x series: JUK-21­(Cu)-*x*_H2_D2_LP (ZIP)
AIF files for H_2_ high-pressure sorption on JUK-21­(Cu)-x series: JUK-21­(Cu)-*x*_H2_HP (ZIP)
AIF files for N_2_ and CO_2_ low-pressure sorption on JUK-21­(Cu)-x series:JUK-21­(Cu)-*x*_N2_CO2 (ZIP)

## Abbreviations

BET: Brunauer–Emmett–Teller (surface area)
CPSM: covalent post-synthetic modification
DFT: density functional theory
DMF: *N*,*N*-dimethylformamide
SI: Supporting Information
GCMC: grand canonical Monte Carlo
HOMO: highest occupied molecular orbital
iEDDA: inverse electron-demand Diels–Alder
IR: infrared spectroscopy
JUK: Jagiellonian University in Krakow
LUMO: lowest unoccupied molecular orbital
MOF: metal–organic framework
NMR: nuclear magnetic resonance
OMS: open metal site
PXRD: powder X-ray diffraction
RDF: radial distribution function
SEM: scanning electron microscopy
TGA: thermogravimetric analysis
UiO: Universitetet i Oslo
UV–vis–NIR: ultraviolet–visible–near-infrared spectroscopy
