# Supplementary material for: Pore Size Engineering of MOFs by Pore Edge Reaction: Tetrazine Click and Hydrogen Adsorption in Theory and Experiment
Source: Chem Mater. 2025 Jul 2;37(14):5206–16. doi: 10.1021/acs.chemmater.5c00914 (PMC12287999; doi:10.1021/acs.chemmater.5c00914)
Supplement: Supplementary file 1 [file cm5c00914_si_001.pdf]

Supporting Information for

Pore Size Engineering of MOFs by Pore Edge Reaction: Tetrazine Click and Hydrogen Adsorption in Theory and Experiment

Damian Jędrzejowski,[a,b] Michał Ryndak,[a] Gabriela Jajko-Liberka,[a] Paweł Kozyra,[a] Witold Piskorz,[a] Volodymyr Bon,[c] Stefan Kaskel,[c] and Dariusz Matoga,[a]

[a]*Faculty of Chemistry, Jagiellonian University, Gronostajowa 2, 30-387 Kraków, Poland*

[b]*Doctoral School of Exact and Natural Sciences, Jagiellonian University, Łojasiewicza 11, 30-348 Kraków, Poland*

[c]*Department of Inorganic Chemistry, Technische Universitat Dresden, Bergstrasse 66, 01062 Dresden, Germany*

Table of contents

S1. Materials 3
S2. Instrumentation 3
S3. Experimental procedures 5
S3.1. Synthesis of organic linker 5
S3.2. Synthesis of MOFs 6
S3.3. iEDDA post-synthetic modifications of JUK-21(Cu) 7
S4. Structural analysis 8
S4.1. JUK-21(Zn) crystal structure 9
S4.2. H_4tztc crystal structure 10
S4.3. JUK-21(Cu) crystal structure 12
S4.4. JUK-21(Cu)-x modelled crystal structures 13
S4.5. JUK-21(Cu)-x torsion angle analysis 21
S5. NMR spectra analysis 25
S5.1. NMR spectra of H_4tztc and JUK-21(Cu) 25
S5.2. NMR spectra of JUK-21(Cu) after iEDDA 27
S5.3. Calculation of iEDDA reaction yield 38
S6. IR spectra 39
S7. UV-vis-NIR electron spectroscopy 41
S8. TG and defect analysis 43
S9. SEM images 47
S10. Nitrogen and carbon dioxide sorption isotherms studies and BET surface area calculations 51
S11. High pressure hydrogen sorption isotherms studies 54
S12. Low pressure hydrogen and deuterium sorption isotherms studies 57
S12.1. Low pressure hydrogen and deuterium adsorption and selectivity calculations by JUK-21(Cu) 57
S12.2. Low pressure hydrogen adsorption by JUK-21(Cu)-x 59
S12.3. Isosteric heat of hydrogen adsorption by JUK-21(Cu)-x 60
S13. Theoretical studies on iEDDA reaction 62
S14. Nitrogen and hydrogen adsorption isotherms simulations 65
S14.1. Structures models and force field optimization 65
S14.2. Structural properties of computational models – helium void fractions, pore volumes and pore size distributions 67
S14.3. High pressure hydrogen adsorption – isotherm simulation 68
S14.4. High pressure hydrogen adsorption – average occupation profiles 69
S14.5. High pressure hydrogen adsorption – radial distribution function 70
S14.6. Low pressure hydrogen adsorption – heat of adsorption 76
S14.7. Low pressure nitrogen adsorption – isotherm simulation 79
S15. References 81

S1. Materials

Substrates: Znic(II) nitrate hexahydrate ($Zn(NO_3)_2 \cdot 6H_2O$, Avantor, >98%), Copper(II) nitrate trihydrate ($Cu(NO_3)_2 \cdot 3H_2O$, ChemPur, >98%), 5-aminoisophthalic acid (AmBeed, 98%), Sodium nitrite ($NaNO_2$, ChemPur, >98%), Potassium cyanide (KCN, Alfa Aesar, >96%), Copper(I) cyanide (CuCN, Merck, 99%), Sodium carbonate (Na_2CO_3, Merck, >99.5%), Hydrochloric acid (HCl, Pure Land, 36-38% in H_2O), Ammonia (NH_3, ChemPur, 25% in H_2O), Zinc trifluoromethanesulfonate ($Zn(OTf)_2$, AmBeed, 98%), Hydrazine hydrate ($N_2H_4 \cdot H_2O$, hydrazine 64%, Alfa Aesar), Dicyclopentadiene (Merck, >96%), Hydroquinone (Syntetyka, >96%), 3,3-Dimethyl-1-butene (Acros, >95%), were used as received, without further purification.

Dienophiles: Styrene (**sty**, Alfa Aesar, 99%), 2-Norbornene (**nor**, TCI, >97%) and 1-Octadecene (**ode**, AmBeed, 98%) were used as received, without further purification.
5-phenylbicyclo[2.2.1]hept-2-ene (**phn**) and 5-(tert-butyl)bicyclo[2.2.1]hept-2-ene (**tbn**) were synthesized and purified (for phn - vacuum distillation) according to literature procedures,[1,2] yielding pure dienophiles (verified by ^{1}H NMR and IR).

Solvents: N,N-dimethylformamide (DMF, Eurochem, >97%), Methanol (MeOH, Merck, >99%), Toluene (PhMe, Merck, >99.5%), deuterated dimethylsulfoxide (DMSO-d_6, Deutero, 99.8 atom% D), deuterated sulfuric acid (D_2SO_4, Deutero, 96-98% solution in D_2O, 99.5 atom% D), were used as received, without further purification.

S2. Instrumentation

^{1}H and ^{13}C nuclear magnetic resonance (NMR) spectra were recorded using a JEOL ECZL-400S spectrometer at 298 K and were calibrated on the residual solvent peak (DSMO-d_6: 2.50 ppm). The NMR samples of MOFs before and after functionalization were prepared by digesting 5-10 mg of air-dried material in 8-10 droplets of D_2SO_4 and then diluting it with 0.75 ml of DMSO-d_6.

Infrared (IR) spectra were collected on Nicolet iS10 FT-IR Spectrometer (Thermo Scientific) with diamond iD7 diamond ATR attachment (Attenuated Total Reflection mode). The spectra were collected in a 4000-400 cm^{-1} range with number of scans set to 32.

Single-crystal X-Ray diffraction data were collected on Bruker D8 Quest Eco diffractometer, which was equipped with a Photon II detector, a Mo Kα (λ = 0.71073 Å) radiation source with a graphite monochromator, and an Oxford Cryostream cooling system. Crystals for measurement were extracted from the mother solution and covered with NVH immersion oil. All measurements were performed at 100 K. Data reduction and cell parameter refinement was carried out using Apex software[3] which included the SAINT and SADABS programs. The intensities of reflections for the sample absorption were corrected using the multiscan method. Structures were solved by the intrinsic phasing method and refined anisotropically with the SHELXL-2019[4] refinement package using weighted full-matrix least squares minimisation on F^2 with the Olex 2[5] graphic interface. Soft SHELXT restraints (SIMU, ISOR) were used to correct the geometry of the disordered parts and the thermal parameters of the corresponding atoms. These restraints were necessary to improve the model and stabilize the refinement. Heavy atoms were refined with anisotropic displacement parameters, whereas hydrogen atoms were assigned at calculated positions with thermal displacement (Uiso(H)) parameters fixed to 1.5 Ueq of methyl C and 1.2 of other C atoms. The results of the data collection and refinement have been summarized in Table S3.
CCDC **JUK-21(Zn)** – 2390929, **H_4tztc** – 2390930 contain the supplementary crystallographic

data for all the compounds, respectively. These data can be obtained free of charge from the Cambridge Crystallographic Data Centre www.ccdc.cam.ac.uk.data_request/cif.

Powder X-Ray diffraction patterns were recorded at room temperature (295 K) on a Rigaku Miniflex 600 diffractometer with Cu−Kα radiation (λ = 1.5418 Å) in a 2θ range from 3° to 45° with a 0.02° step at a scan speed of 3° min^{-1}. The powder pattern of JUK-21(Cu) immersed in DMF was measured on STOE STADI P diffractometer equipped with a line-focus Cu X-ray tube operated at 40 kV/30 mA, a focusing Ge (111) monochromator (λ = 0.15405 nm), and a MYTHEN (DECTRIS) detector. The suspension was sealed in a borosilicate capillary (d = 0.5 mm) and the measurement was conducted using 2Theta scans with exposition of 120 s/step and a detector step size of 2θ = 1°.

Nitrogen and carbon dioxide sorption isotherms were measured on a Quantachrome Autosorb iQ-C-XR-XR EPDM instrument. Before measurements, the samples were washed at least three times with methanol (between each wash the sample was incubated in the solvent for 24 h) and then degassed at 25 or 80 °C for 8 or 20 h (for details see Section S3.3. iEDDA post-synthetic modifications of JUK-21(Cu). The sorption measurements were carried out at 77 K for N_2 and at 195 K for CO_2.Temperature control was afforded by using a liquid N_2 bath (77 K) or dry ice – acetone bath (195 K). Brunauer-Emmett-Teller (BET) theory was used to calculate the specific surface area of the materials obtained, using BET Surface Identification (BETSI) software.[6] For all N_2 isotherm analyses extended Roquerol criteria[7] were used, the pressure range optimized to get at least 10 points of linear fit in the BET equation. The pore size distribution plots were derived from sorption data by NLDFT calculations using a silica cylindrical pore model.

High pressure hydrogen sorption isotherms were measured on BELSORP HP Automatic Gas Adsorption Apparatus. As equilibrium conditions, a pressure change of 0.1% upon 300 s was chosen for each point of the isotherm. The dead volume was determined using helium (99.999%). The values for adsorbed hydrogen are given at standard conditions (273.15 K, 101.325 kPa) in $cm^3 \cdot g^{-1}$ or $mg \cdot g^{-1}$. The sorption measurements were carried out at 77 K by using a liquid N_2 bath. The sample (120-250 mg) was placed in a cylindrical stainless steel cell with 0.7 cm diameter, and connected to the instrument using a 3 mm stainless steel capillary.

Low pressure hydrogen and deuterium sorption isotherms were measured on BELSORP-max instrument. As equilibrium conditions, a pressure change of 1% upon 300 s was chosen for each point of the isotherm. The dead volume was determined using helium (99.999%). The values for adsorbed gases are given at standard conditions (273.15 K, 101.325 kPa) in $cm^3 \cdot g^{-1}$. To reach the desired adsorption temperatures, a DE-202AG (ARS-Cryo) cryostat with connected helium cycle and an LS-366 (LAKE SHORE) control unit was used. A water-cooled ARS-2HW helium compressor dissipated produced heat of the cryostat. The sample (30-60 mg) was placed in a self-made 3 cm long, cylindrical copper cell with 1 cm diameter closed by a copper dome, isolated by dynamic vacuum ($p < 10^{-4}$ kPa), and connected to the instrument using a 3 mm stainless steel capillary.

Scanning Electron Microscopy (SEM) images were collected on Helios 5 PFIB Dual Beam Scanning Electron Microscope (SEM, Thermo Fisher Scientific) in immersion mode.

Thermogravimetric analysis were performed at atmospheric pressure under flowing argon (or synthetic air) on a Mettler-Toledo TGA/SDTA 851e instrument at a heating rate of 5°C min^{-1} in the temperature range of 25−600°C (for measurements in argon flow) or 25-1000°C (for measurements in air flow) (sample weights were ca. 50 mg).

Carbon, hydrogen, and nitrogen content were determined by conventional microanalysis with the use of an Elementar Vario MICRO Cube elemental analyzer.

Electron UV-vis-NIR reflectance spectra were recorded on a Shimadzu UV-2101PC spectrometer equipped with an ISR-260 attachment.

S3. Experimental procedures

S3.1. Synthesis of organic linker

5,5'-(1,2,4,5-tetrazine-3,6-diyl)diisophthalic acid (H_4tztc): The synthesis of the ligand H_4tztc was carried out in three steps: (1) the synthesis of an organic nitrile using the Sandmeyer reaction, (2) the cyclization of the nitrile to dihydrotetrazine, and (3) its oxidation to an s-tetrazine (see Figure S1).

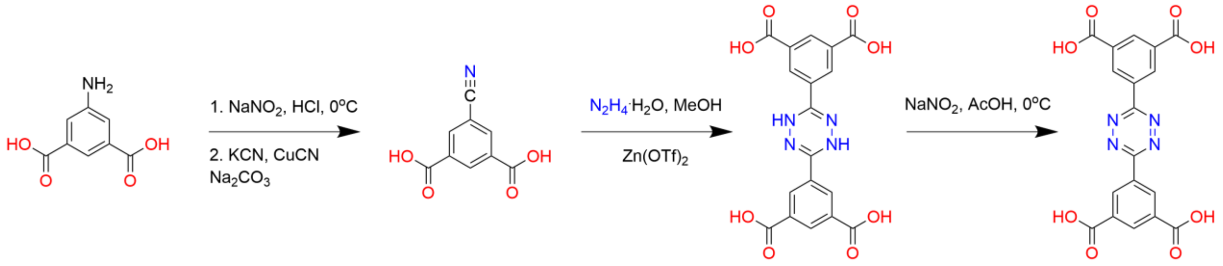

Figure S1. Scheme of H_4tztc ligand synthesis.

(1) ***Synthesis of 5-cyanoisophthalic acid***
5-Aminoisophthalic acid (7.24 g, 40.0 mmol) was suspended in 10 mL of deionized water in a 250 mL round-bottom flask, and 10 mL of concentrated hydrochloric acid was carefully added with stirring. After 30 minutes of stirring at 25 °C, the mixture was cooled to 0 °C using an NaCl-ice bath. A sodium nitrite solution (3.16 g, 45.8 mmol of $NaNO_2$ in 8 mL of water) was then added dropwise using a dropping funnel, ensuring that the temperature remained below 5 °C. The resulting suspension contained yellow diazonium chloride.

Meanwhile, in a 400 mL beaker, KCN (8.98 g, 138 mmol), CuCN (4.12 g, 46.0 mmol), and Na_2CO_3 (4.00 g, 33.7 mmol) were mixed in 26 mL of water and heated to 60 °C until complete dissolution (~10 min). All subsequent operations were conducted under a closed fume hood with proper ventilation. The cooled diazonium salt suspension was added to the heated cyanide mixture using a pipette. After complete addition, both the flask and beaker were rinsed with distilled water, and the resulting brown mixture was cooled to room temperature. The beaker was then placed in an ice-water bath to prevent HCN evolution, and concentrated hydrochloric acid was carefully added dropwise until the yellowish precipitate fully formed (~20 mL of HCl was added). The suspension was filtered, washed with water, and the solid residue was treated with 28 mL of concentrated ammonia solution before being filtered again (the solid residue contained Cu(I), which rapidly oxidized upon air exposure, turning blue). The filtrate was diluted with 50 mL of water, placed in an ice-water bath, and concentrated hydrochloric acid was added again to precipitate the product. The yellowish solid was recrystallized from water (once or twice), dried in a vacuum oven, yielding 3.16 g (41%) of beige solid 5-cyanoisophthalic acid.

^{1}H NMR (400 MHz, DMSO-d$_6$, ppm): 13.78 (2H, s), 8.64 (1H, t, J = 1.6Hz), 8.49 (2H, d, J = 1.6Hz).
^{13}C NMR (100 MHz, DMSO-d$_6$, ppm): 165.1, 136.5, 133.8, 132.7, 117.4, 112.7.
FT-IR (ATR, cm^{-1}): 2500-3200 (br s, $\nu_{O\text{-}H}$, CO**OH**), 2241 (m, $\nu_{C\equiv N}$), 1710 (s, $\nu_{C=O}$, **CO**OH), 1250-1280 (m, $\nu_{C\text{-}O}$, **COO**H).
Melting point: 249 °C (lit. 251 °C).

(2) ***Cyclization to dihydrotetrazine***

The second and third steps were optimized variants of the Pinner tetrazine synthesis with a different catalyst.[8] A 100 mL round-bottom flask was purged with argon, and 5-cyanoisophthalic acid (1.00 g, 5.23 mmol), $Zn(OTf)_2$ (485 mg, 1.33 mmol), and 7 mL of methanol were introduced under an argon atmosphere. The mixture was heated to 60 °C on a magnetic stirrer until the substrates dissolved. Then, $N_2H_4 \cdot H_2O$ (8.10 mL, 162 mmol) was added, leading to the formation of a light brown precipitate. The mixture was stirred at 60 °C for 14 hours under an argon overpressure. After completion, the reaction mixture was cooled and quantitatively transferred (rinsing with a small amount of distilled water) into a tall beaker, which was placed in an ice-water bath. The dihydrotetrazine intermediate was not isolated but immediately subjected to the next reaction step.

(3) ***Oxidation to H_4tztc***

Oxidation was carried out by gradually adding ~50 mL of 50% acetic acid to the yellow suspension obtained in step 2. An aqueous $NaNO_2$ solution was then added until the precipitate color changed from dark yellow to red and foaming ceased. The precipitate was centrifuged, washed with water, and suspended in ~60 mL of 10% hydrochloric acid. The mixture was stirred on a magnetic stirrer for 1 hour, after which the precipitate was centrifuged and washed three times with distilled water. The dark pink product was dried in a vacuum oven (80 °C, 10 mbar), yielding 596 mg of H_4tztc (nitrile-based yield: 55%).

^{1}H NMR (400 MHz, DMSO-d_6, ppm): 13.74 (0.6H, s), 9.26 (4H, d, J = 1.7Hz), 8.74 (2H, t, J = 1.6Hz). Peak assignment in the Figure S16.
^{13}C NMR (100 MHz, DMSO-d_6, ppm): 165.9, 162.7, 133.4, 132.8, 132.7, 131.8. Peak assignment in the Figure S17.
FT-IR (ATR): 2600-3400 (br s, $\nu_{O\text{-}H}$, CO**OH**), 1720 (s, $\nu_{C=O}$, **CO**OH), 1370 (m, $\nu_{C=N\ Ar}$, **Tz**) 1200-1250 (m, $\nu_{C\text{-}O}$, **C**O**O**H).
Melting point: ~330 °C

Single-Crystal Growth

Single crystals of H_4tztc were obtained by dissolving 10 mg of the compound in 2 mL of DMF. The vial containing this solution was subjected to slow vapor diffusion with water. After approximately one week, violet-red single crystals formed. These crystals were used for structural determination, providing additional confirmation of the intended molecular structure (Section S4.2. H_4tztc crystal structure).

S3.2. Synthesis of MOFs

JUK-21(Zn): 143 mg (0.481 mmol) of $Zn(NO_3)_2 \cdot 6H_2O$ and 98.0 mg (0.239 mmol) of the H_4tztc ligand precursor were dissolved in DMF (15.0 mL) with ultrasonication. The mixture was heated at 100 °C for 8 hours, with time control playing a critical role in this synthesis. The resulting red crystals (crystallite size of 100–200 µm) were filtered off and washed several times with DMF. Yield: 63 mg (30%). iEDDA modifications of the material were attempted without additional solvent exchange; however, for sorption measurements (and where indicated), guest molecules were exchanged with methanol by immersing the sample in 20 mL of methanol at least three times for 24 hours each. The prepared sample was then dried and stored under a protective atmosphere.

JUK-21(Cu): 29.0 mg (0.120 mmol) of $Cu(NO_3)_2·3H_2O$ and 24.5 mg (0.060 mmol) of the H_4tztc ligand precursor were dissolved in DMF (15.0 mL) using ultrasonication and subsequently heated in a sealed vial at 100 °C for 24 hours. The resulting polycrystalline purple product (crystallite size < 5 μm) was filtered off and washed several times with DMF. Yield: 26 mg (49%). Material for the iEDDA reaction was prepared according to the procedure described in Section S3.3. iEDDA post-synthetic modifications of JUK-21(Cu). For most measurements, the sample was prepared by exchanging guest molecules with methanol by immersing the sample in 20 mL of methanol at least three times for 24 hours each. The prepared sample was then dried and stored under a protective atmosphere.

S3.3. iEDDA post-synthetic modifications of JUK-21(Cu)

JUK-21(Cu)-x: After washing with DMF, the JUK-21(Cu) material was stored as a suspension in DMF before preparing the iEDDA reaction. The concentration of JUK-21(Cu) in the suspension was determined as follows: 200 μL of the thoroughly shaken suspension was taken, placed on a watch glass of precisely known mass, and completely dried on a heating plate at 100 °C. The mass of the solid residue was multiplied by five to determine the concentration, e.g., 38.5 mg_{MOF}/mL.

A volume of the suspension containing 200 mg (~0.254 mmol) of MOF (e.g., 5.19 mL) was centrifuged, and the supernatant was removed. To the solid residue, 10 mL of toluene was added, followed by shaking and centrifugation. The solid was then quantitatively transferred to a 20 mL vial using 7 mL of toluene. Dienophile was added in either a 5-fold or 15-fold molar excess (see Table S1). Temperature and reaction time were optimized based on reaction conversion rate verification and are provided in Table S1.

The completeness of the reaction was monitored by a visible color change (Figure S42), UV-vis reflectance spectra (Figure S44), a reduction in the intensity of the 1380–1410 cm^{-1} band in infrared spectra (Figure S39-Figure **S40**), and the disappearance of signals from the tetrazine ligand along with the formation of peaks from the new ligand in the ^1H NMR spectra after digestion (Section S5.2. NMR spectra of JUK-21(Cu) after iEDDA).

Upon completion, the product was centrifuged and washed once with hot toluene (stirred at the reaction temperature for 60 min), twice with toluene at room temperature (to remove excess dienophile), and twice with methanol. The product was subsequently immersed in methanol, with the solvent replaced every 24 hours for three cycles. The final sample was dried and stored under a protective atmosphere. It is noteworthy that despite the different color of the samples suspended in methanol, each has the same purple-blue color after activation.

Table S1. Synthetic details of the reaction of JUK-21(Cu) with dienophiles.

**Dienophile**	**Mass used** (per 200 mg of MOF)	**Reaction temperature** (°C)	**Reaction time** (hrs)	**Product colour** (in methanol)
**sty**	132 mg (1.27 mmol)	60	6	Green
**nor**	120 mg (1.27 mmol)	60	2	Blue
**tbn**	191 mg (1.27 mmol)	100	36	Green-blue
**phn**	216 mg (1.27 mmol)	60	12	Blue
**ode**	962 mg (3.81 mmol)	100	72	Navy blue

The vacuum-activated materials (after solvent exchange with methanol) were initially analyzed using CHNS elemental composition analysis. Activation was intended to remove all guest molecules, and analyzing the material post-activation ensured that any remaining guests were limited to water molecules adsorbed from atmospheric moisture. In terms of skeletal composition, the most relevant parameters are the carbon and nitrogen content, and in particular their ratio, while the hydrogen content also reflects the presence of residual water. Table S2 compiles the experimental elemental composition alongside the calculated theoretical values. The number of water molecules was found to be relatively consistent and depended primarily on the duration of exposure to air. For the JUK-21(Cu)-ode sample, the theoretical composition was adjusted to reflect incomplete conversion (82% yield, as determined in Section S5.3). Full molecular formulas are additionally provided in Table S4.

Table S2. The results of elemental analysis for a series of activated JUK-21(Cu)-x MOFs.

MOF	Composition		%C	%N	%H	%C / %N
**JUK-21(Cu)**	{[Cu_2(tztc)]·2H_2O}	exp	38.08	9.58	2.081	3.975
		calc	37.97	9.83	1.770	3.861
		|exp-calc|	0.11	0.25	0.31	0.114
**-sty**	{[Cu_2(tztc-sty)] 4H_2O}	exp	46.17	4.23	2.969	10.915
		calc	45.96	4.12	2.968	11.152
		|exp-calc|	0.21	0.11	0.00	0.237
**-nor**	{[Cu_2(tztc-nor)] 4H_2O}	exp	44.91	4.17	3.547	10.770
		calc	44.85	4.19	3.313	10.716
		|exp-calc|	0.06	0.02	0.23	0.053
**-tbn**	{[Cu_2(tztc-tbn)] 5H_2O}	exp	46.76	3.86	4.245	12.114
		calc	46.83	3.77	4.337	12.422
		|exp-calc|	0.07	0.09	0.09	0.308
**-phn**	{[Cu_2(tztc-phn)] 5H_2O}	exp	48.54	3.72	3.52	13.048
		calc	48.76	3.67	3.695	13.288
		|exp-calc|	0.22	0.05	0.18	0.240
**-ode**	{[Cu_2(tztc-ode)$_{0.82}$ (tztc)$_{0.18}$] 4H_2O}	exp	49.53	4.66	5.335	10.629
		calc	49.25	4.48	5.181	10.991
		|exp-calc|	0.28	0.18	0.15	0.362

The data presented in Table S2 demonstrate good agreement between the expected and experimentally determined elemental composition of the MOFs after the iEDDA reactions. While this type of analysis reflects the average composition across the bulk sample, it is subject to certain limitations. Therefore, further evidence for the occurrence of the iEDDA reactions, along with both qualitative and quantitative characterization of the resulting products, is presented in the following sections.

S4. Structural analysis

S4.1. JUK-21(Zn) crystal structure

Data collection and reduction

The details on obtaining a diffraction data for a single crystal were discussed in Section S2. Instrumentation.

Structure solution and refinement

The phase problem was solved by the intrinsic phasing method and positions of all non-hydrogen atoms were determined using SHELXT program.[9] All non-hydrogen atoms were refined anisotropically using weighted full-matrix least-squares on F^2. Refinement and further calculations were carried out using SHELXL-2019.[4] Soft SHELXT restraints (SIMU, ISOR) were used to correct the thermal parameters of the C8 and N9 atoms. These restraints were necessary to improve the model and stabilize the refinement.
The results of the data collection and refinement have been summarized in Table S3.

Hydrogen atoms treatment

All hydrogen atoms joined to carbon atoms were positioned with an idealized geometry and refined using a riding model with Uiso(H) fixed at 1.5 Ueq of methyl C and 1.2 of other C atoms. Hydrogen atoms joined to nitrogen were also positioned with an idealized geometry like in aromatics and refined using a riding model with Uiso(H) fixed at 1.2 Ueq of origin nitrogen atom (AFIX 43).

Notes

The electron densities corresponding to the disordered solvent molecules that are unreachable from Fourier difference map were flattened using the 'SQUEEZE' option of PLATON.[10] This procedure was used to avoid the problems with disordered DMF molecules and to improve convergence process.

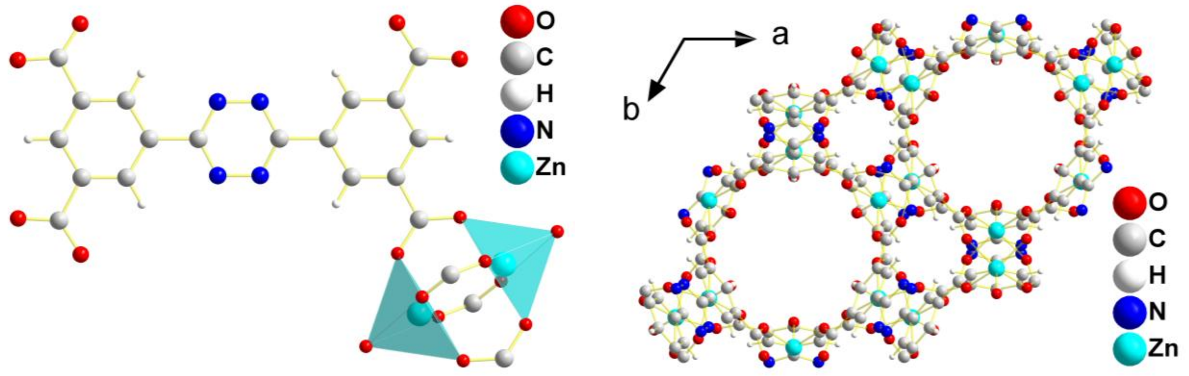

Figure S2. Structural fragments of the JUK-21(Zn) material: ligand and zinc cation coordination environment (paddlewheel type) (right) and packing in the [001] direction (left).

S4.2. H_4tztc crystal structure

Data collection and reduction

The details on obtaining a diffraction data for a single crystal were discussed in Section S2. Instrumentation.

Structure solution and refinement

The phase problem was solved by the intrinsic phasing method and positions of all non-hydrogen atoms were determined using SHELXT program.[9] All non-hydrogen atoms were refined anisotropically using weighted full-matrix least-squares on F^2. Refinement and further calculations were carried out using SHELXL-2019.[4]

The results of the data collection and refinement have been summarized in Table S3.

Hydrogen atoms treatment

All hydrogen atoms joined to carbon atoms were positioned with an idealized geometry and refined using a riding model with Uiso(H) fixed at 1.5 Ueq of methyl C and 1.2 of other C atoms.

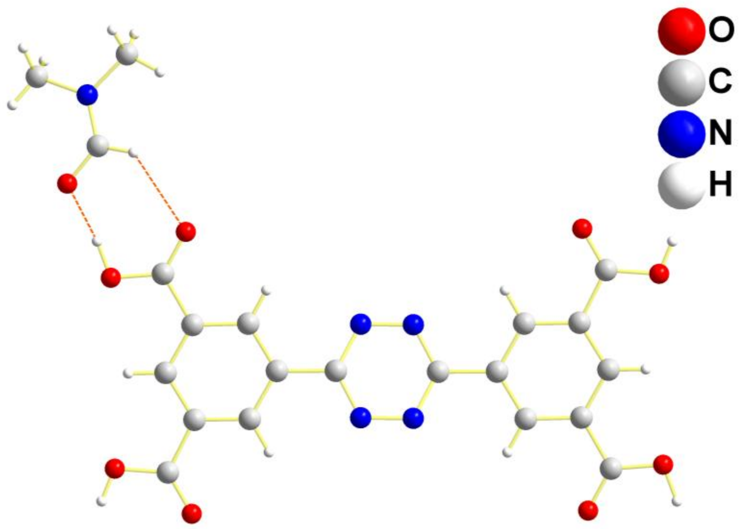

Figure S3. Structural visualization of the solvated (DMF) H_4tztc precursor ligand. Hydrogen bonds are marked with dashed lines in red.

Table S3. Structural data and parameters of JUK-21(Zn) and H_4tztc materials.

Identification code	JUK-21(Zn)	tztc-DMF
**CCDC**	2390929	2390930
**Empirical formula**	$C_{45}H_{51}N_{12}O_{21}Zn_3$	$C_{24}N_6O_{10}H_{24}$
**Formula weight**	1292.08	556.49
**Temperature/K**	100.00	100(2)
**Crystal system**	trigonal	monoclinic
**Space group**	R-3m	$P2_1/c$
**a/Å**	18.5797(6)	10.5314(3)
**b/Å**	18.5797(6)	8.8351(3)
**c/Å**	37.8495(18)	13.1177(4)
**α/°**	90	90
**β/°**	90	91.0990(10)
**γ/°**	120	90
**Volume/Å^3**	11315.4(9)	1220.32(7)
**Z**	6	2
**ρ_{calc}g/cm^3**	1.138	1.514
**μ/mm^{-1}**	1.010	0.120
**F(000)**	3978.0	580.0
**Crystal size/mm^3**	0.2 × 0.2 × 0.15	0.2 × 0.15 × 0.1
**Radiation**	MoKα (λ = 0.71073)	MoKα (λ = 0.71073)
**2θ range for data collection/°**	4.384 to 51.366	5.56 to 51.368
**Index ranges**	-22 ≤ h ≤ 21, -21 ≤ k ≤ 22, -45 ≤ l ≤ 46	-12 ≤ h ≤ 12, -10 ≤ k ≤ 10, -15 ≤ l ≤ 15
**Reflections collected**	34061	22601
**Independent reflections**	2633 [R_{int} = 0.0917, R_{sigma} = 0.0440]	2298 [R_{int} = 0.0338, R_{sigma} = 0.0262]
**Data/restraints/parameters**	2633/12/85	2298/0/229
**Goodness-of-fit on F^2**	1.083	1.070
**Final R indexes [I>=2σ (I)]**	R_1 = 0.0532, wR_2 = 0.1680	R_1 = 0.0339, wR_2 = 0.0866
**Final R indexes [all data]**	R_1 = 0.0655, wR_2 = 0.1790	R_1 = 0.0355, wR_2 = 0.0884
**Largest diff. peak/hole / e·Å^{-3}**	0.45/-0.40	0.17/-0.27

S4.3. JUK-21(Cu) crystal structure

Despite extensive efforts, optimization of the JUK-21(Cu) synthesis conditions did not yield crystallites of a size suitable for conventional single-crystal structural measurements. Numerous attempts were made to slow down crystallization, including lowering the synthesis temperature, changing the solvent to a less hydrolyzing alternative (DEF and DMA), reducing substrate concentration, adding an acid modulator, and employing an alternative synthesis method based on slow substrate diffusion. However, these modifications either led to a reduction in synthesis yield (while maintaining crystallite size) or resulted in a contaminated or degraded product. In several cases, the addition of an acid modulator yielded single crystals of the precursor, H_4tztc, instead of the desired material.

Preliminary studies suggest that JUK-21(Cu) is isostructural with its zinc analogue, the crystal structure of which is reported in Section S4.1. JUK-21(Zn) crystal structure. Furthermore, the secondary paddlewheel-type building unit is well-documented for copper-based structures but is relatively uncommon for zinc. Other investigations, particularly sorption studies, indicate that JUK-21(Cu) does not exhibit flexibility and retains its structure upon guest molecule exchange and removal. These findings strongly support the hypothesis that JUK-21(Cu) adopts a structure analogous to its zinc counterpart.

To further validate this assumption, the most probable structure of JUK-21(Cu) was modelled using isomorphic substitution and force-field optimization. The theoretical powder pattern generated for this model was compared with the experimental pattern of JUK-21(Cu) containing DMF (Figure S4), with background contributions from excess liquid and disordered guest molecules removed for clarity.

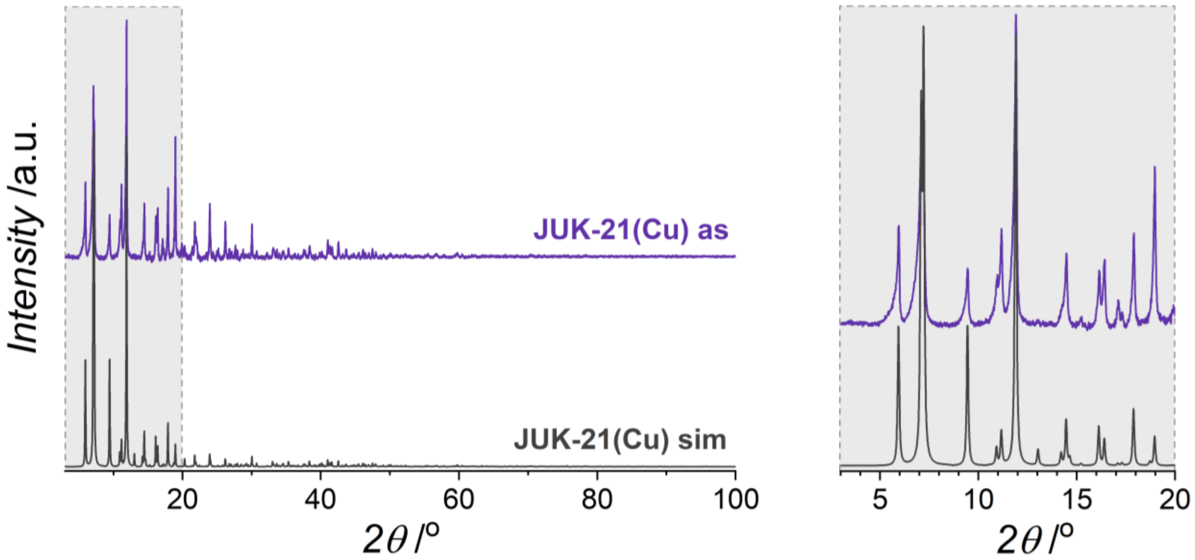

Figure S4. Powder X-Ray patterns for JUK-21(Cu) material: generated for the simulated and optimized structure (**sim**, in black) and registered for the original sample immersed in DMF (**as**, in purple). The gray highlighted area is enlarged and inserted on the right.

For a more precise structural determination, a Pawley refinement was performed to obtain unit cell parameters, followed by a Rietveld refinement to resolve the crystal structure. The derived unit cell parameters were consistent with the simulated JUK-21(Cu) structure. However, refinement of the crystal structure encountered difficulties due to the presence of DMF molecules, which were challenging to model because of the structure's high symmetry. Measuring an activated sample (devoid of guest molecules) to circumvent this issue was not feasible for technical reasons and due to the nature of the sample.

Nevertheless, the crystal structure of JUK-21(Cu) is further supported not only by the comparison of simulated and experimental powder patterns but also by additional findings. These include nitrogen and hydrogen adsorption profiles, which align with theoretical predictions, and molecular-level evidence obtained from NMR and IR spectroscopy (see Sections S5.1. NMR spectra of H_4tztc and JUK-21(Cu) and S6. IR spectra .

S4.4. JUK-21(Cu)-x modelled crystal structures

The structures of MOF materials prepared by modifying JUK-21(Zn) CIF structure by substituting Zn with Cu. Both structures were optimized for the method. For the reason that CIF structure of synthesized products are not available, in the next step a series of dienophiles were substituted in the JUK-21(Cu) structure according synthesized materials - with a series of dienophiles (sty, nor, tbn, phn, ode). Preservation of the structure skeleton was confirmed by X-ray diffraction, and the formation of modified ligand forms was confirmed by NMR and IR spectroscopy.

All the quantum-chemical calculations of geometries, energies, and electron properties including orbital energy, orbital contours, Fukui functions were performed at the periodic DFT level of theory with use of VASP[11,12], which utilizes the PAW method[13,14] to reconstruct the all–electron wave function. The Gaussian smearing (preferred over the Methfessel–Paxton smearing[15] for the description of insulators) with $\sigma = 0.01$ eV was used. The following optimization criteria were used: energy change of 10^{-6} eV between two successive steps for the SCF, gradient norm of 10^{-3} eV/Å for the geometry optimization. A basis set plane–wave cutoff energy of 500 eV and the PBE[16,17] (from the GGA family) correlation–exchange functional was used. The dispersion interactions were accounted for at the post-SCF, semiempirical method of Grimme[18–20]. Brillouin zone sampling was achieved using a standard Monkhorst–Pack grid[21] [13] with a 3 × 3 × 3 mesh.

Figure S5 compares the experimental powder patterns (as) of each obtained sample with the pattern generated from the simulated and optimized crystal structure (sim). This comparison confirms the preservation of crystallinity after each modification and demonstrates that the model accurately reflects the actual structure, at least in terms of unit cell parameters.

Figures Figure S6-Figure S11 provide a detailed comparison of the obtained structures from different perspectives. Figure S6 illustrates the basic building unit: the ligand and the coordination unit. Figure S7 and Figure S8 presents the structural packing along the [001] direction (corresponding to the [111] direction for primitive unit cells after modifications), highlighting the primary free-space channels and their size reduction after modification. Figure S9 shows the packing along the [-111] direction (corresponding to the [100] direction for primitive unit cells), emphasizing ligand modifications and the spatial arrangement of introduced functional groups within the MOF backbone. Figure S10 depicts the packing along the [100] direction (corresponding to the [1-10] direction for primitive unit cells), illustrating the alignment of introduced functional groups relative to the metal cluster. Lastly, Figure S11 presents the packing along the [241] direction (corresponding to the [-111] direction for primitive unit cells), highlighting the alignment of introduced functional groups with the free-space channel undergoing the greatest size reduction.

In each figure, all atoms (except the carbon atoms building the backbone of the introduced dienophile) are uniformly colored in gray for clarity.

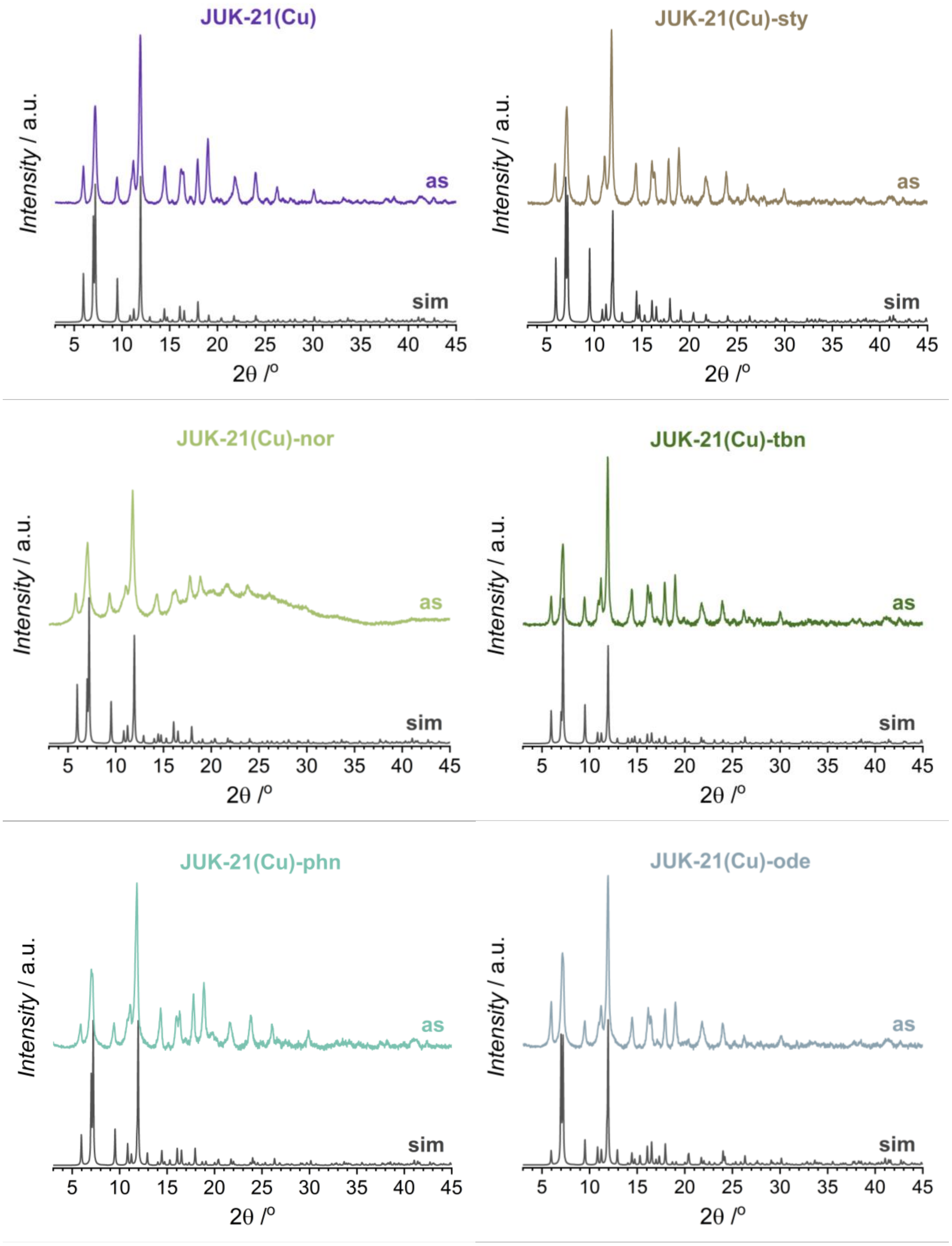

Figure S5. Experimental (as) vs simulated (sim) powder patterns for each material in JUK-21(Cu)-x series.

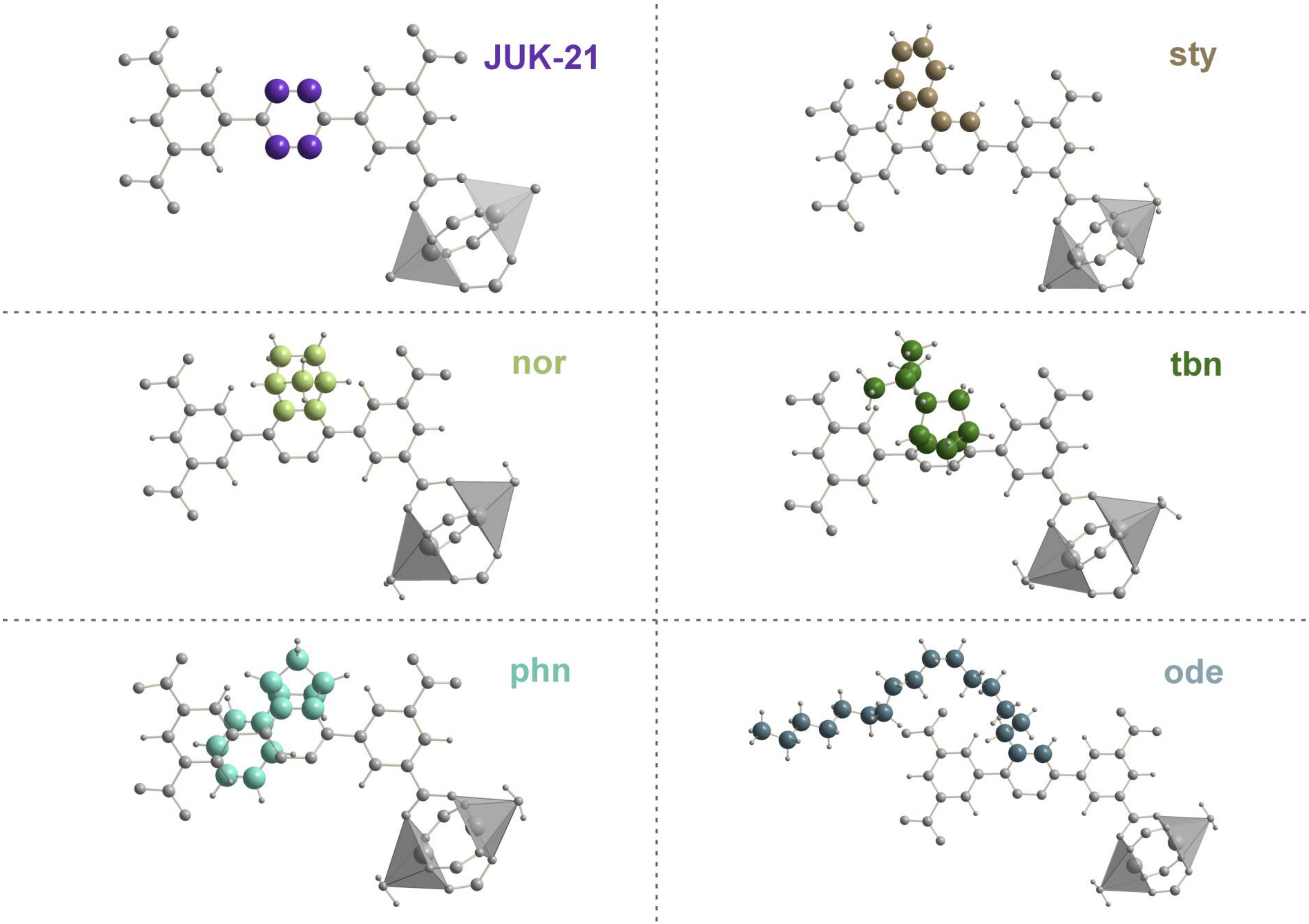

Figure S6. Basic building unit of JUK-21(Cu)-x series: the ligand and the coordination unit.

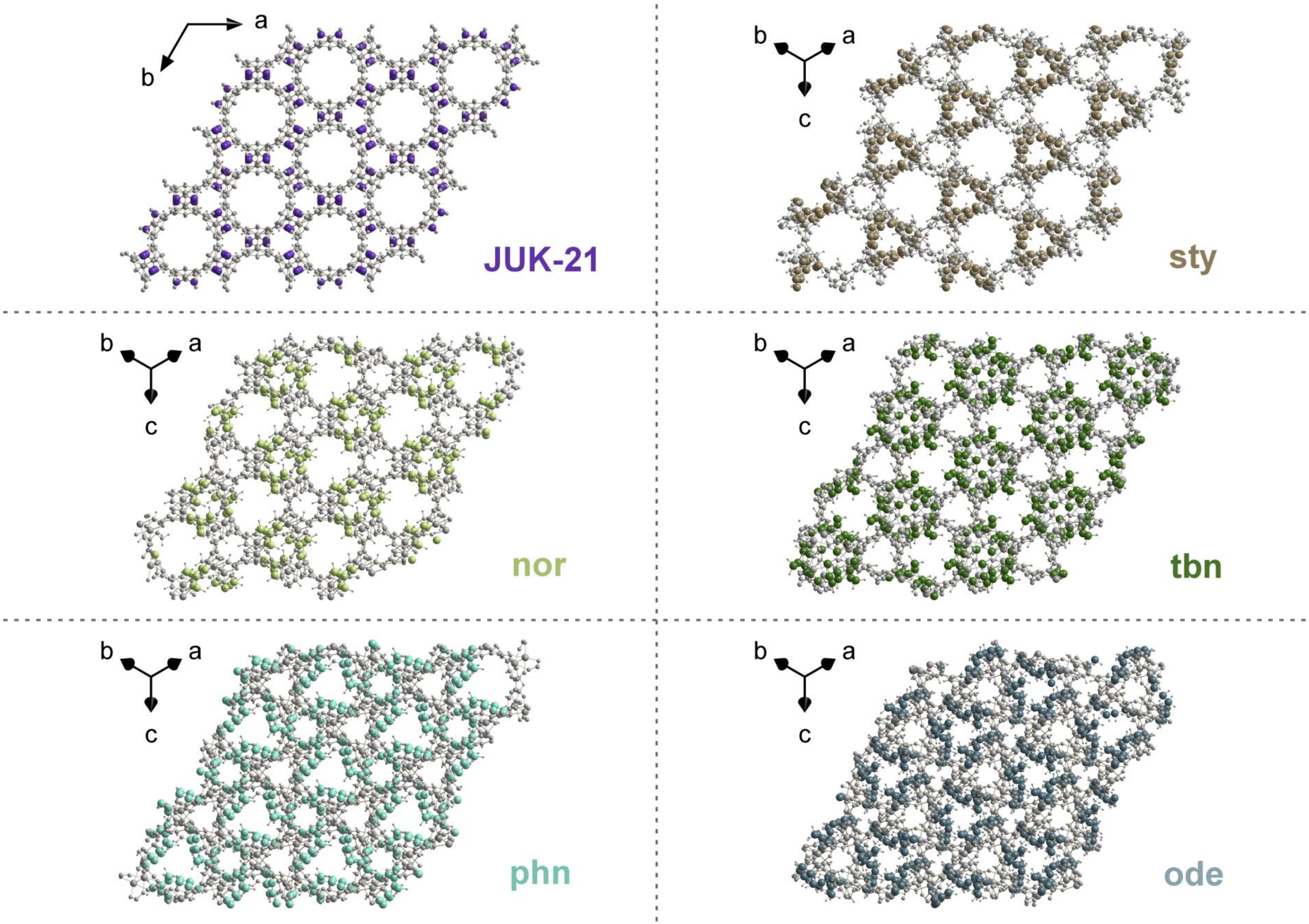

Figure S7. Structure packing along the [001] ([111]) direction, highlighting the primary free-space channels and their size reduction after modification.

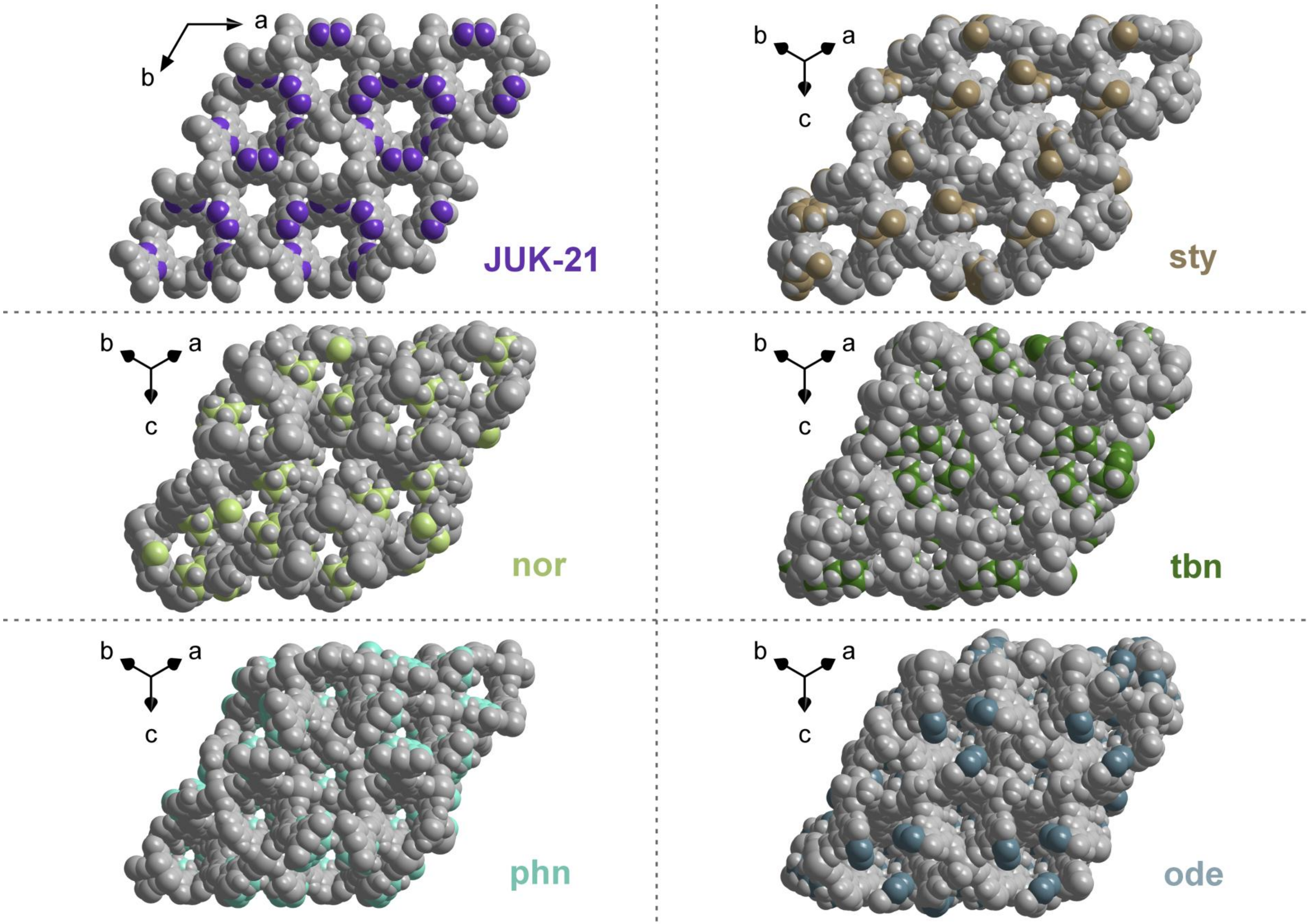

Figure S8. Structure packing along the [001] ([111]) direction, spacefill mode to address van der Waals radii of atoms.

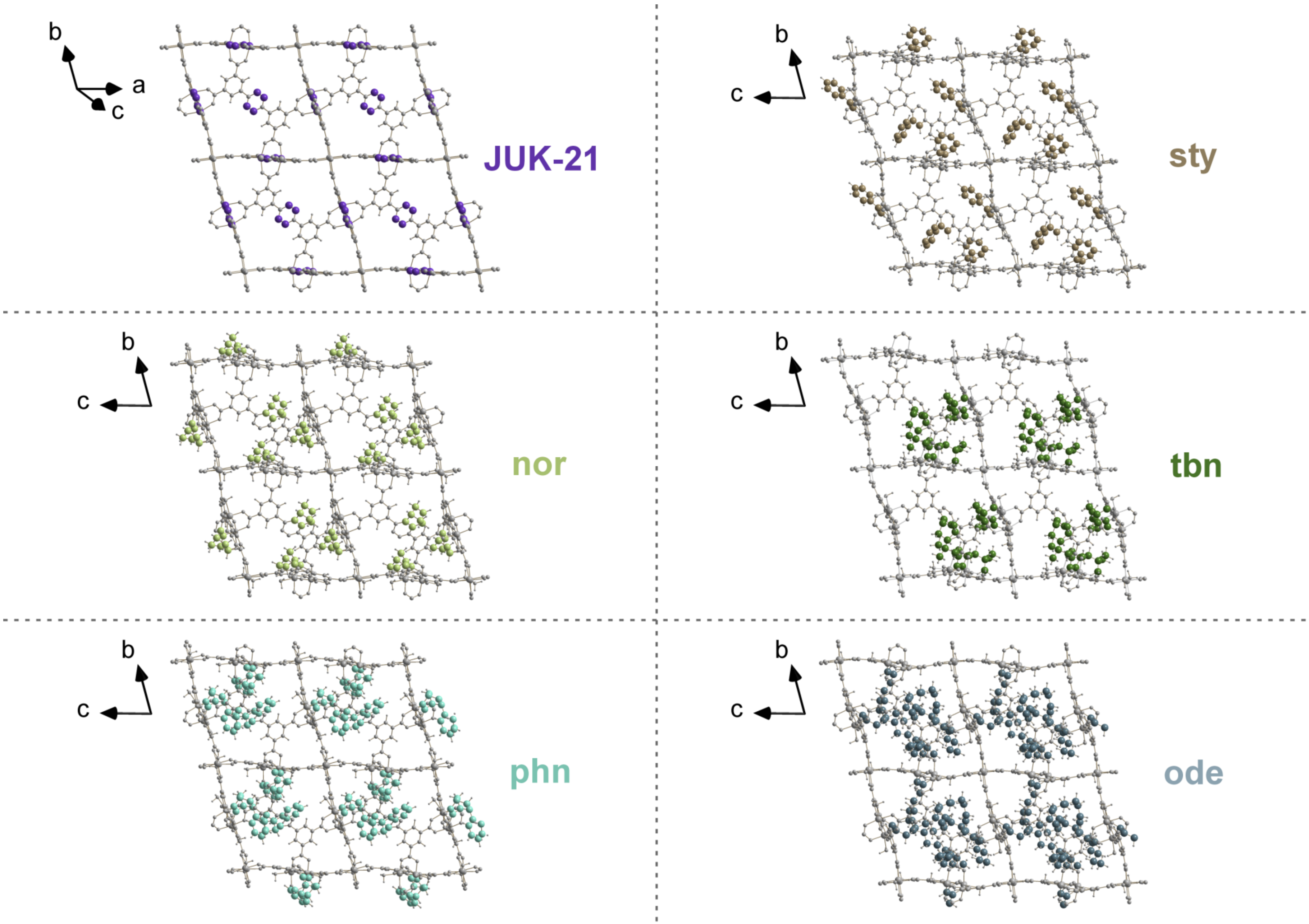

Figure S9. Structure packing along the [-111] ([100]) direction, emphasizing ligand modifications and the arrangement of introduced functional groups within the MOF backbone

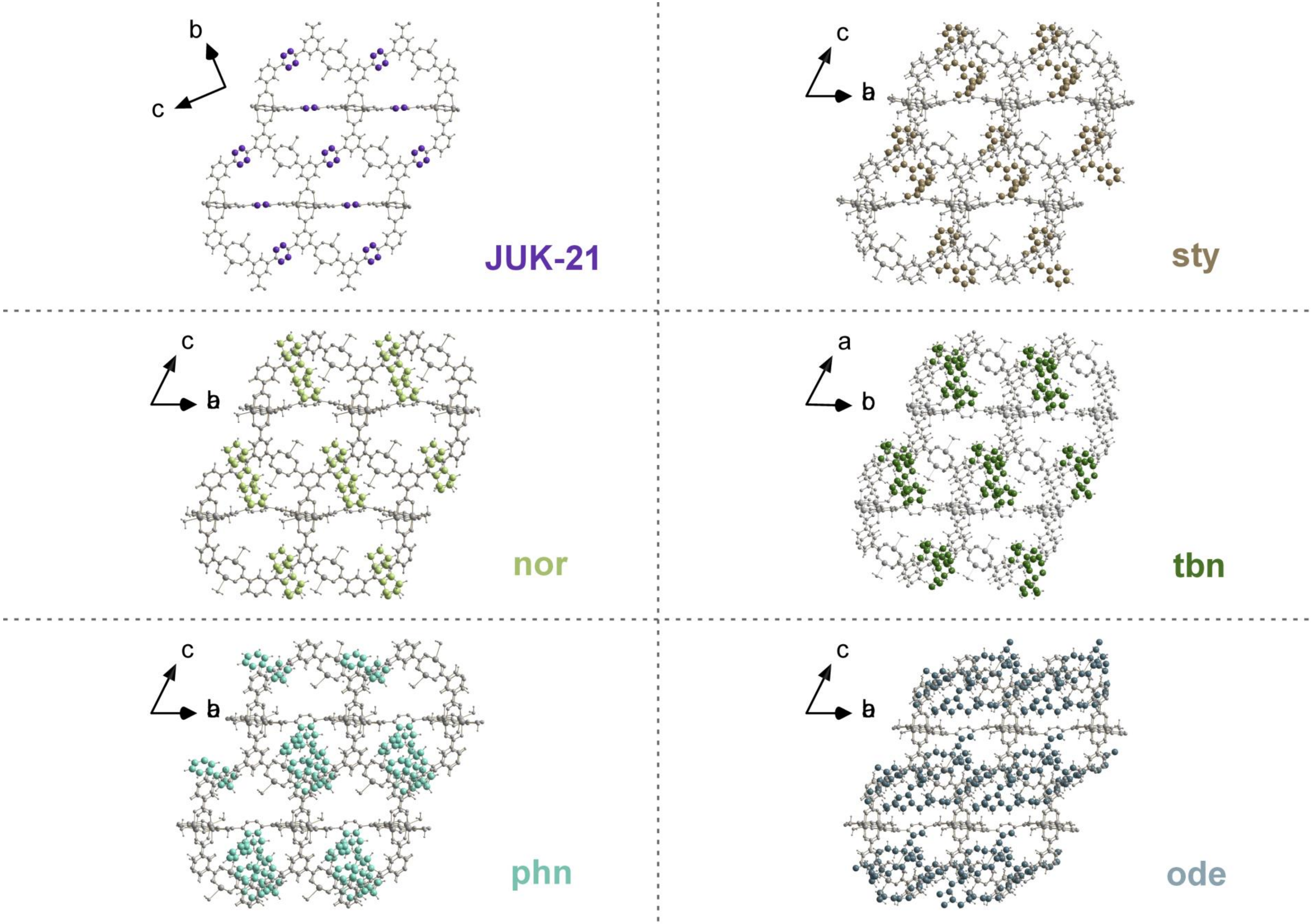

Figure S10. Structure packing along the [100] ([1-10]) direction, illustrating the alignment of introduced functional groups relative to the metal cluster

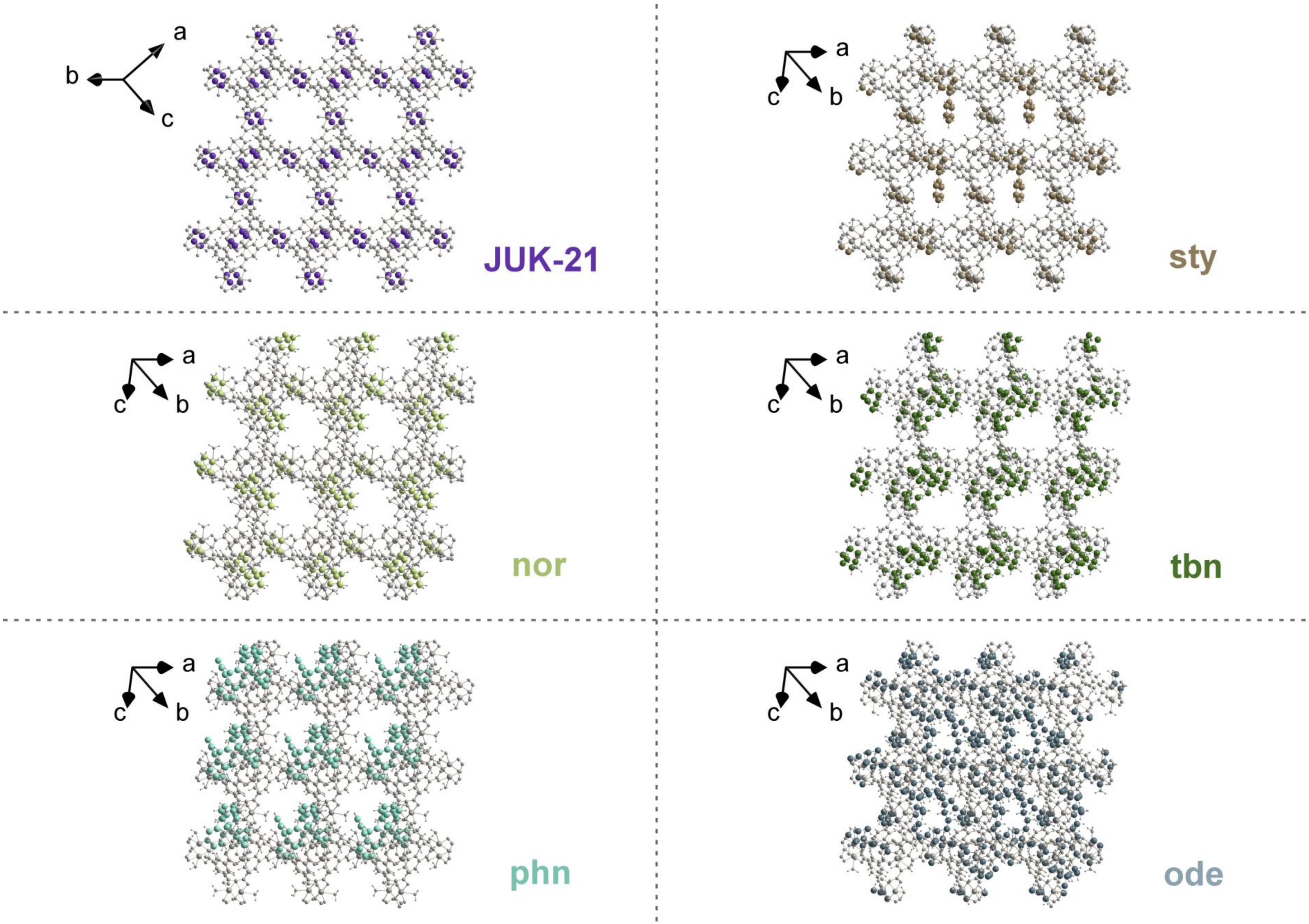

Figure S11. Structure packing along the [241] ([-111]) direction, highlighting the alignment of functional groups with the free-space channel undergoing the greatest size reduction.

S4.5. JUK-21(Cu)-x torsion angle analysis

An in-depth analysis of the modelled crystal structures of JUK-21(Cu)-x materials enables verification of the effect of the introduced functional group on specific structural parameters, providing insights into the degree of stabilization or destabilization following iEDDA modification.

The $tztc^{4-}$ ligand consists of three aromatic rings: two phenyl rings and a central tetrazine ring (cf. Figure S6). The coplanarity of these rings ensures periodic separation of the metal nodes, which defines the *nbo* topology of the material. Any deviation from this arrangement may contribute to additional stabilization or destabilization of the structure. To assess these effects, selected angles were analyzed in the optimized structural models of each JUK-21(Cu)-x representative.

Figure S12 presents two perspectives highlighting the analyzed angles. The first perspective projects the ligand along the Ar-Ar bond axis, illustrating the torsion between ring planes. The second perspective is aligned parallel to the plane of the first (arbitrarily chosen) phenyl ring, visualizing the overall bending of the ligand induced by the introduction of a functional group.

The torsion angles between the rings were measured by analyzing each set of four consecutively bonded atoms (two from the first ring, two from the second ring), yielding four combinations per rings pair. The mean value represents the dihedral angle between the planes of the rings, while the standard deviation indicates measurement uncertainty. The second pair of adjacent rings was analyzed analogously, yielding two values: the Ph_R-Tz angle (between the right phenyl ring and the middle tetrazine or diazine ring) and the Ph_L-Tz angle (between the left phenyl ring and the middle ring). Here, the right ring is conventionally defined as the one closest to the bulkier moiety of the introduced functional group (for non-symmetric dienophiles). Figure S13 presents the average torsional angles for each structure along with their uncertainties.

The ligand bending angle was determined by calculating the centroid of each of the three aromatic rings and measuring the angles between them. The resulting values are also provided in Figure S13.

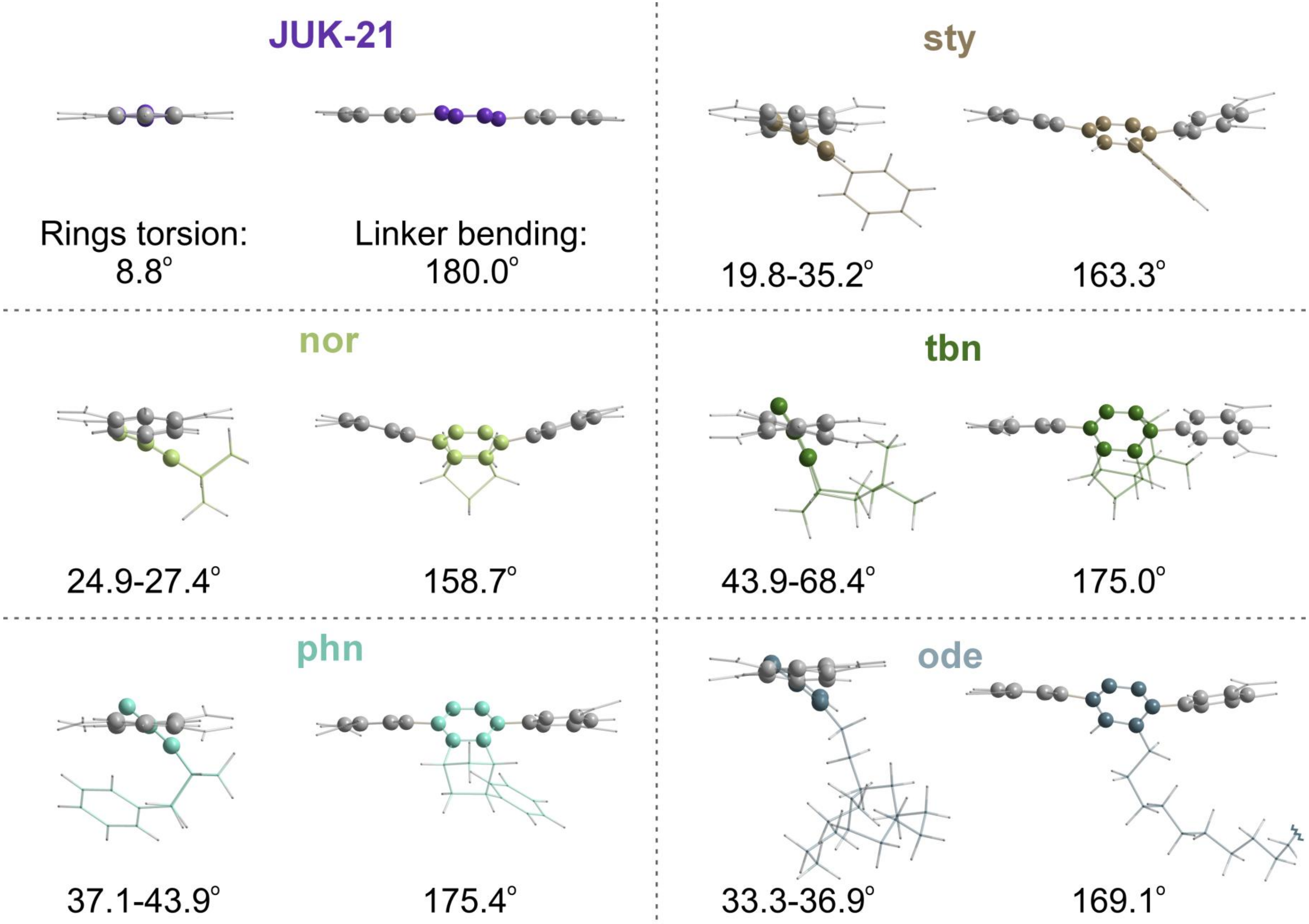

Figure S12. Structural analysis of torsion and bending angles in a series of JUK-21(Cu)-x materials.

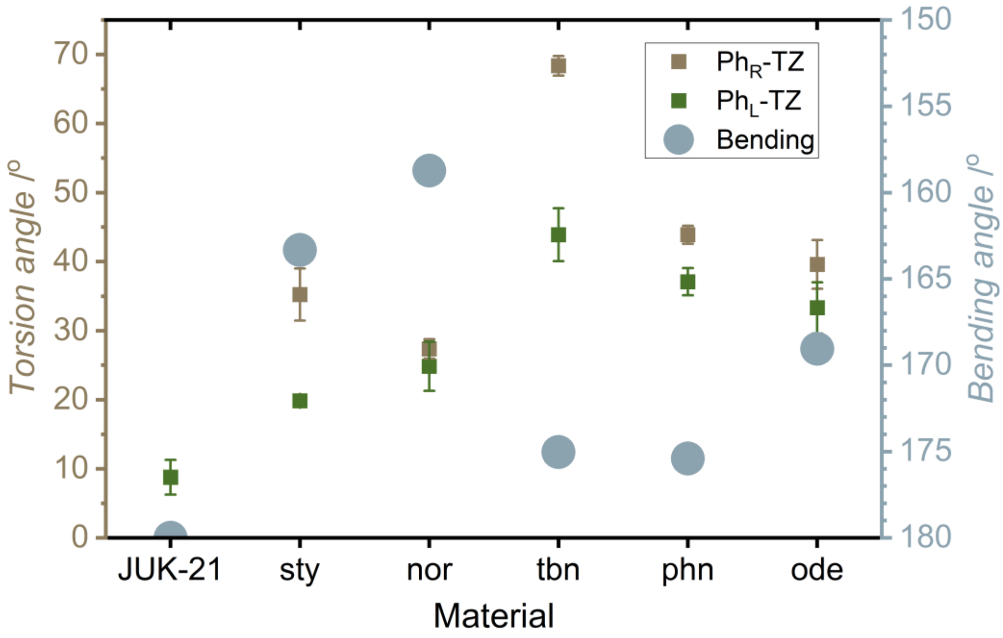

Figure S13. Visualisation of torsion (squares) and bending (circles) angles for each material. Lower part of graph represents least distorted molecule.

The analysis reveals that the stability of the material is influenced not only by the volume of the introduced functional group but, more importantly, by interactions between the nearest dienophile fragments and neighboring aromatic rings, as well as the asymmetry of the dienophile itself. Experimental studies indicate that **JUK-21(Cu)-sty** and **JUK-21(Cu)-nor** are less stable than the parent material, which corresponds well with the most pronounced ligand shape deformation (bending angle deviating furthest from 180°).

In **JUK-21(Cu)-sty**, instability manifests through the formation of additional missing-linker defects. This is attributed to the destabilizing effect of the additional ring, which induces twisting in the adjacent phenyl ring, ultimately disrupting coordination bonds and leading to partial ligand de-coordination.

In contrast, **JUK-21(Cu)-nor** features a symmetric ligand center, where the Ph_L-Tz twist is compensated by the Ph_R-Tz twist. However, the key factor is the cumulative effect of both twists, which results in a significant bending of the entire ligand to 159°. This induces maximum structural destabilization by elongating coordination bonds and introducing internal structural stress, making the material particularly unstable, especially upon guest molecule removal.

For **JUK-21(Cu)-tbn** and **JUK-21(Cu)-phn**, despite both containing a norbornene fragment, the structural effects differ significantly. Although these materials exhibit the largest torsional angles in the series, the destabilizing effect is mitigated by the fact that the bulky functional group is positioned further from the ligand's three-ring aromatic system. Consequently, ring twisting is compensated, and the overall ligand bending remains below 5°, preventing coordination bond shortening and internal stress buildup. Additionally, in **JUK-21(Cu)-phn**, the introduced functional group engages in intramolecular π–π interactions (4.21Å, Figure S14), further stabilizing the structure. As a result, both materials exhibit greater stability than the parent compound (cf. Figure S56).

Finally, **JUK-21(Cu)-ode** exhibits intermediate levels of torsional strain and ligand bending. Here, the destabilization remains minor, and the stability is primarily influenced by the large

functional group volume and the cumulative effect of weak interactions introduced by the alkyl chains.

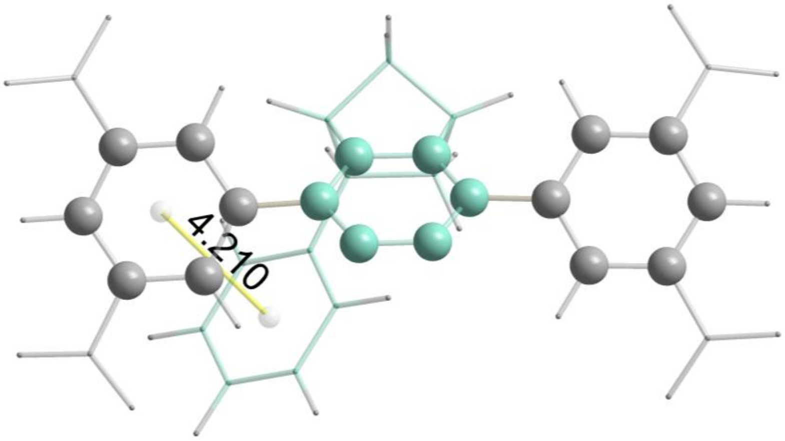

Figure S14. Visualisation of stabilizing intramolecular π-π interaction in JUK-21(Cu)-phn.

S5. NMR spectra analysis

S5.1. NMR spectra of H_4tztc and JUK-21(Cu)

The synthesis of the H_4tztc ligand is described in Section S3.1. Synthesis of organic linker, and a list of ^{1}H NMR and ^{13}C NMR peaks is provided there. Figure S15 shows the annotation of hydrogen and carbon atoms, along with the color code used in Figure S16 and Figure S17 to assign peaks from the corresponding atoms. The lower intensity of the H_A peak is associated with the rapid hydrogen-deuterium exchange in the solvent used.

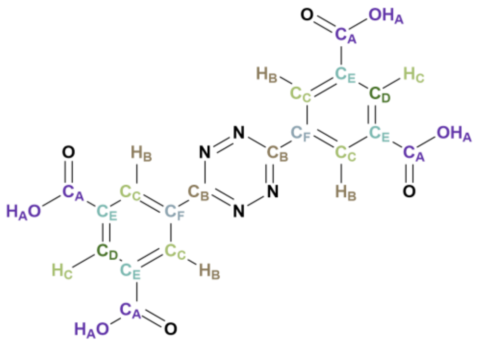

Figure S15. Structure of the H_4tztc ligand precoursor with assigned (subscripts and color code) hydrogen and carbon atoms, for interpretation of ^{1}H and ^{13}C NMR spectra.

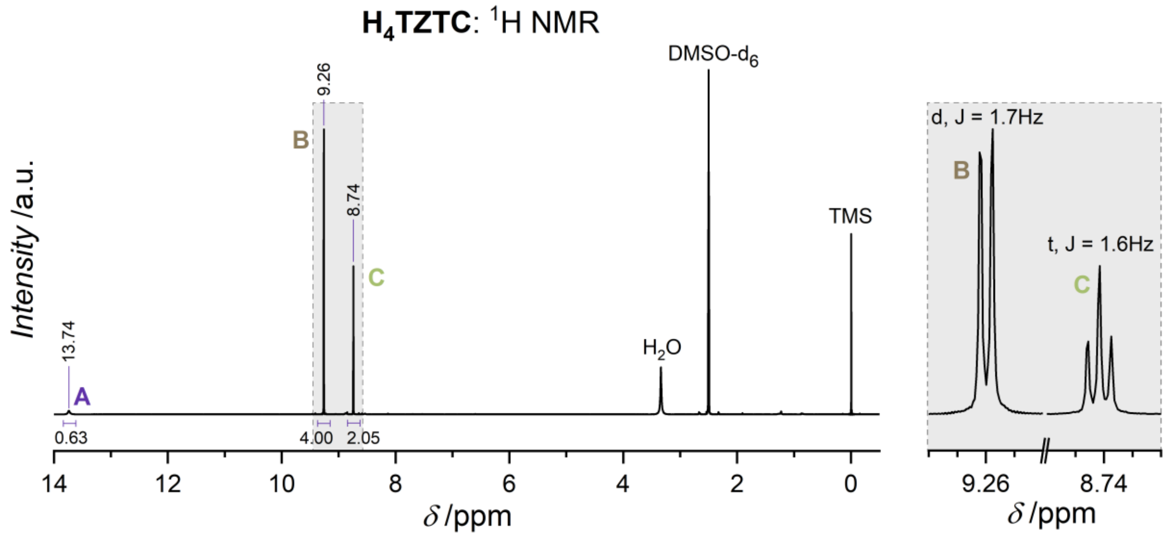

Figure S16. ^{1}H NMR spectrum of H_4tztc with assigned peaks (Figure S15). On the right, zoom in on the area of the aromatic peaks.

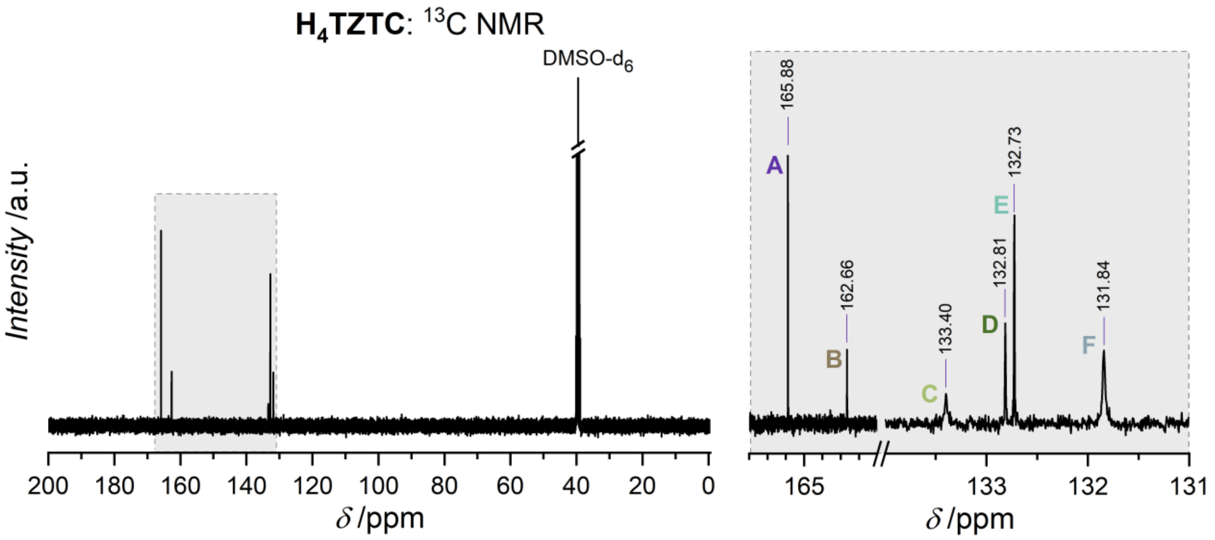

Figure S17. ^{13}C NMR spectrum of H_4tztc with assigned peaks (Figure S15). On the right, zoom in on the area of the carbonyl and aromatic peaks.

To verify the content of JUK-21(Cu), a sample of digested material was prepared with a mixture of D_2SO_4 and DMSO-d_6. The resulting spectrum is presented in Figure S18. Despite the interference associated with the presence of paramagnetic copper(II) ions, the peaks are not so broadened as to hinder their interpretation. The presence of additional peaks (DCM, H_2O) is associated with residual solvents remaining in the pores of the material or as solvent impurity. The peaks recorded are at positions corresponding to the protonated H_4tztc (compare with Figure S16) with a shift of 0.01-0.03 ppm toward the high field, due to the effect of D_2SO_4 addition.

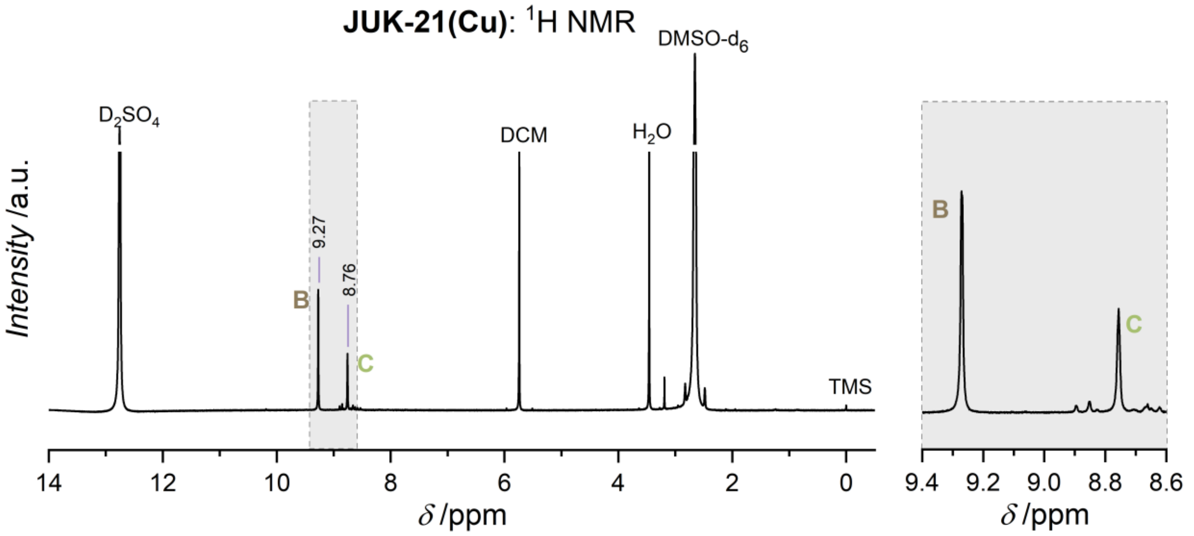

Figure S18. ^1H NMR spectrum of JUK-21(Cu) after digestion in D_2SO_4/DMSO-d_6, with assigned peaks (Figure S15). On the right, zoom in on the area of the aromatic peaks.

S5.2. NMR spectra of JUK-21(Cu) after iEDDA

For materials obtained with iEDDA reactions, interpretation of NMR spectra is considerably more complicated than for JUK-21(Cu). Most dienophiles generate molecular asymmetry (sty, tbn, phn, ode), and some were additionally used as a mixture of isomers (tbn, phn: in both the *endo* isomer dominates). Adding to this the broadening of peaks due to the presence of paramagnetic Cu(II), the situation becomes further complicated, and for this reason two different samples were prepared for each material. The first, by direct dilution of the material after solvent exchange with methanol, resulting in a ^{1}H NMR spectrum. To improve its readability and to be able to characterize the peaks more in-depth, an additional procedure was used for the digestion and extraction of the ligand itself.

Samples of JUK-21(Cu)-x materials were dissolved as follows: after a complete washing procedure with toluene and methanol (compare with Section S3.3. iEDDA post-synthetic modifications of JUK-21(Cu)), 50-100 mg of the dried sample was placed in a beaker, 5-10 mL of concentrated H_2SO_4 was added, stirred and sonicated for 1 minute, resulting in complete dissolution of the MOF. Then, while stirring vigorously, 50 mL of distilled water was slowly and carefully added. After the mixture cooled to room temperature, a precipitate appeared in several cases. The suspension was extracted with three 50 mL portions of ethyl acetate, the precipitate was filtered and dried, the organic layer was washed with brine, dried with anhydrous sodium sulfate and the solvent evaporated. The resulting precipitate was combined with the precipitate obtained at an earlier stage (ensured that they contained the same substances, as verified by ^{1}H NMR). ^{1}H and ^{13}C NMR spectra were recorded for the ligand thus obtained.

The following pages illustrate a series of 4 figures for each modified material, respectively: assignment of hydrogen and carbon atoms (Figure S19, Figure **S23**, Figure **S27**, Figure **S34**), ^{1}H NMR spectrum after direct digestion in D_2SO_4/DMSO-d_6 (Figure S20, Figure **S24**, Figure **S28**, Figure **S31**, Figure **S35**), ^{1}H NMR spectrum (Figure S21, Figure **S25**, Figure **S29**, Figure **S32**, Figure **S36**) and ^{13}C NMR spectrum (Figure S22, Figure **S26**, Figure **S30**, Figure **S33**, Figure **S37**) for ligands extracted from the digested MOF sample (JUK-21(Cu)-x_**ext**).

Each data series has been annotated with additional comments. The first spectrum for each material should be considered primarily as information on the degree of the tetrazine system's conversion (for this reason, grid lines corresponding to the peaks of the unreacted ligand have been drawn), the other two spectra present detailed information on the presence of various isomers/variants of the product and provide quantitative data.

JUK-21(Cu)-sty

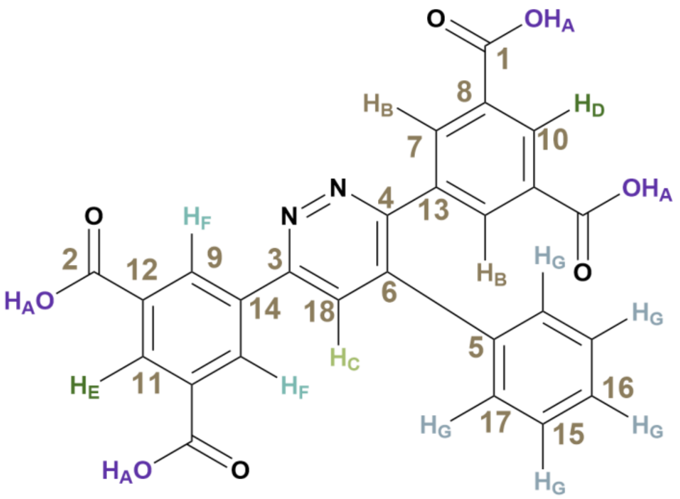

Figure S19. Tags assignment to hydrogen and carbon atoms for H_4tztc-sty ligand.

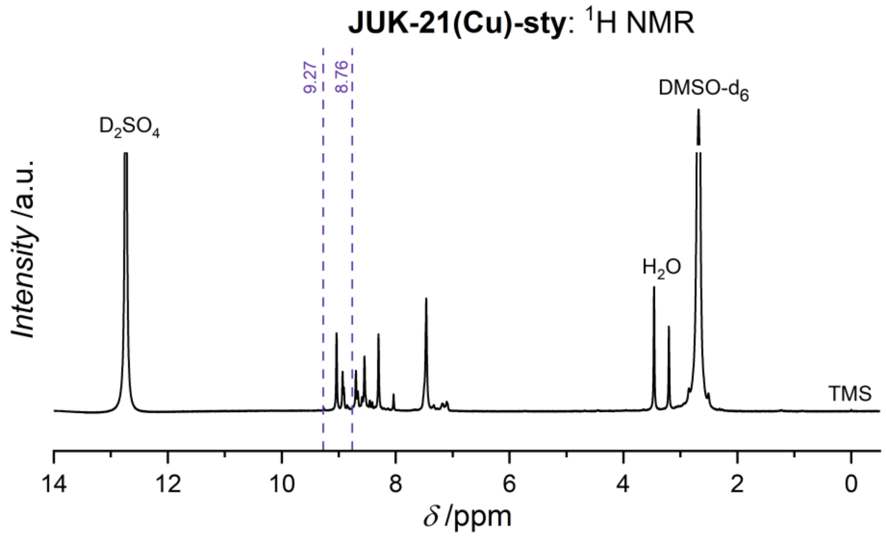

Figure S20. ^{1}H NMR spectra of JUK-21(Cu)-sty after direct digestion in D_2SO_4/DMSO-d_6.

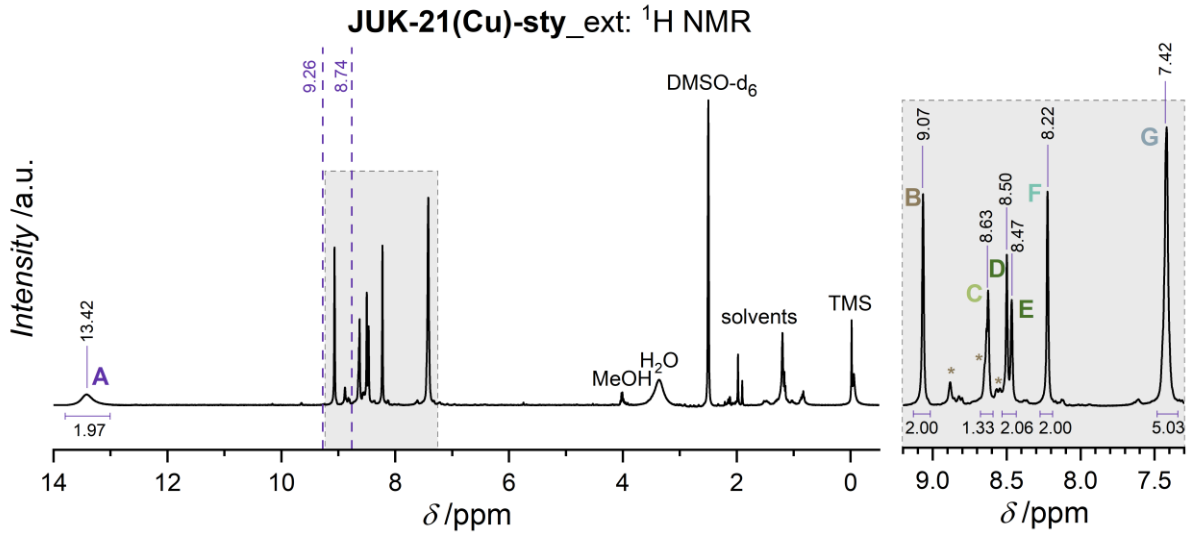

Figure S21. ^{1}H NMR spectra of H_4tztc-sty after digestion and extraction of JUK-21(Cu)-sty.

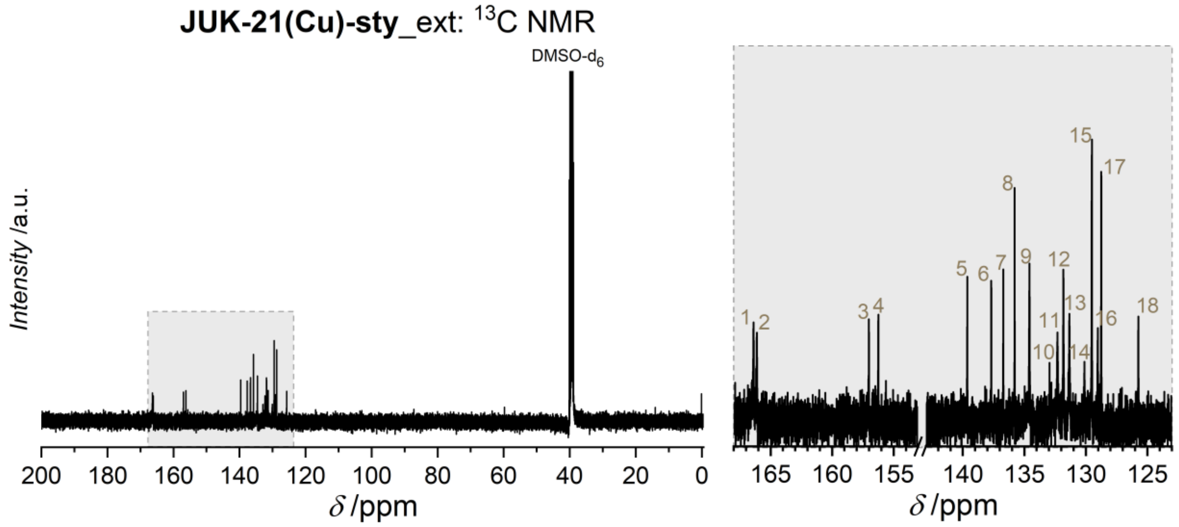

Figure S22. ^{13}C NMR spectra of H_4tztc-sty after digestion and extraction of JUK-21(Cu)-sty.

H_4tztc-sty ^{13}C NMR peaks (tags from Figure S19): **1**: 166.40, **2**: 166.12, **3**: 157.02, **4**: 156.25, **5**: 139.64, **6**: 137.69, **7**: 136.71, **8**: 135.79, **9**: 134.59, **10**: 132.95, **11**: 132.31, **12**: 131.82, **13**: 131.34, **14**: 130.11, **15**:129.51, **16**: 129.03, **17**:128.74, **18**: 125.73

Comments to spectra for JUK-21(Cu)-sty:

On both proton spectra (Figure S20 andFigure **S21**), peaks can be seen only in the aromatic part. In addition, there are no peaks corresponding to unreacted tetrazine, indicating full conversion to the product. On the close-up part of the spectrum in Figure S21, peaks of low intensity marked with an asterisk can be observed. They originate from a side product, dihydrodiazine (see comments to JUK-21(Cu)-nor), but it is trace in content and qualitatively does not affect the structure of the material whatsoever (it has a similar conformation and identical skeleton, differing only by two hydrogen atoms).

On the carbon spectrum (Figure S22) all 18 peaks are clearly visible, all in the aromatic part. Notably, the asymmetry of the molecule after the introduction of an additional functional group (e.g., peaks 1 and 2 from carboxyl groups on two sides of the molecule) is apparent on this spectrum.

JUK-21(Cu)-nor

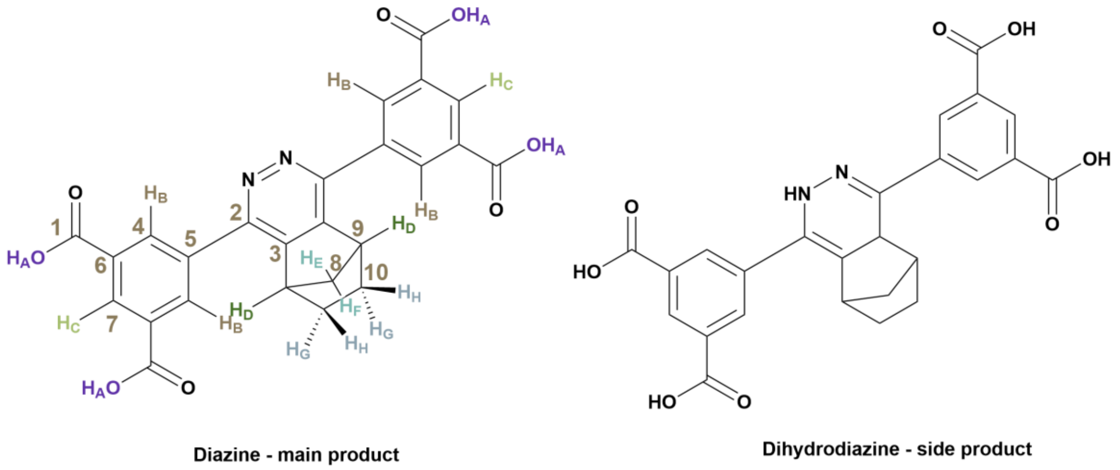

Figure S23. Tags assignment to hydrogen and carbon atoms for H_4tztc-nor ligand (left). The structure of the side product (dihydrodiazine, right), marked with asterisks on the following spectra.

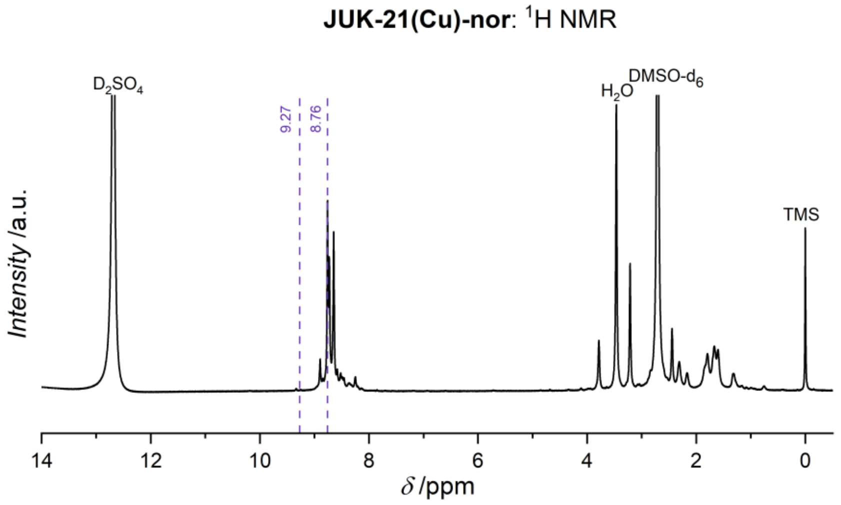

Figure S24. ^{1}H NMR spectra of JUK-21(Cu)-nor after direct digestion in D_2SO_4/DMSO-d_6.

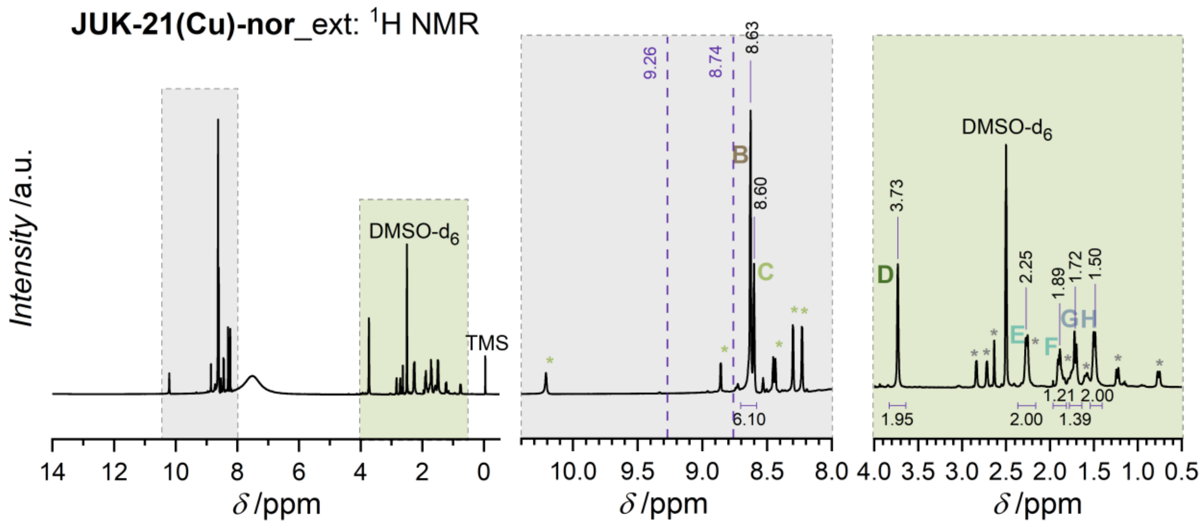

Figure S25. ^{1}H NMR spectra of H_4tztc-nor after digestion and extraction of JUK-21(Cu)-nor.

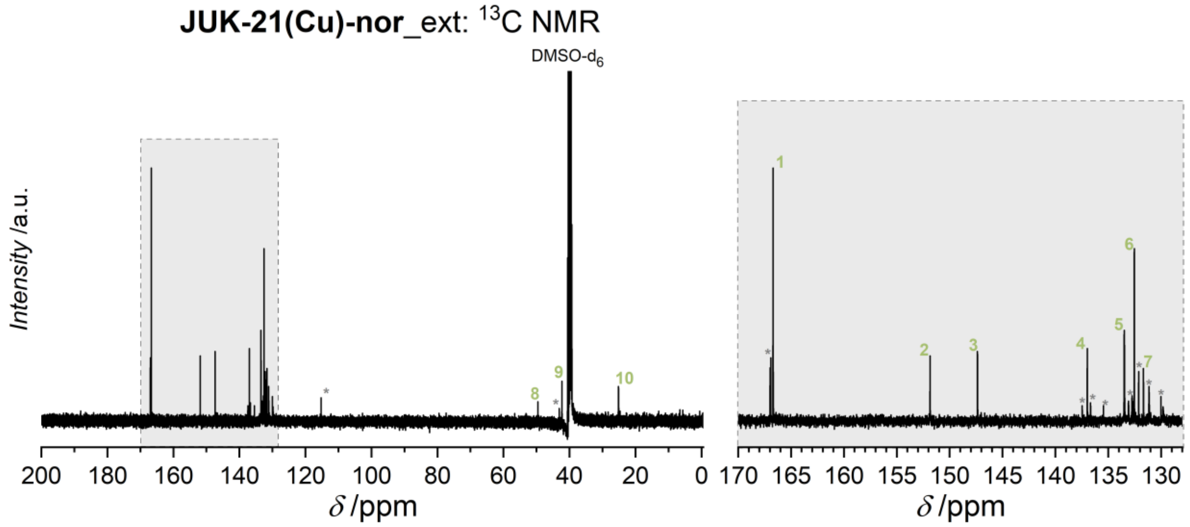

Figure S26. ^{13}C NMR spectra of H_4tztc-nor after digestion and extraction of JUK-21(Cu)-nor.

H_4tztc-nor ^{13}C NMR peaks (tags from Figure S23): **1**: 166.70, **2**: 151.85, **3**: 147.37, **4**: 136.98, **5**: 133.48, **6**: 132.54, **7**: 131.67, **8**: 49.69, **9**: 42.30, **10**: 25.15

Comments to spectra for JUK-21(Cu)-nor:

For both proton spectra (Figure S24 and Figure **S25**), it can be seen that the product has no unreacted tetrazine. Peaks in both the aromatic and aliphatic regions are visible, but for the directly digested sample (Figure S24) the broadening of the peaks makes accurate interpretation impossible. For the extracted sample (Figure S25), peaks from the side product, dihydrodiazine (cf. Figure S23), are clearly visible - peaks from this form are marked with asterisks on the spectra. Since H_4tztc-nor is the only one of the analyzed products that is symmetric relative to the central ring, the peaks on the ^{1}H and ^{13}C NMR spectra are low enough in intensity and are well enough separated that they can easily be assigned to both the main and side product. The presence of the side product does not affect the structure of the MOF backbone in any way, it only slightly differs in conformation from the aromatic ligand. In addition, its amount is so small that even negligible conformational effects cannot affect the whole material.

On the carbon spectrum (Figure S26) all 10 peaks are clearly visible, 3 of them in aliphatic part. Peaks of side product are also present, both in aromatic and aliphatic part.

JUK-21(Cu)-tbn

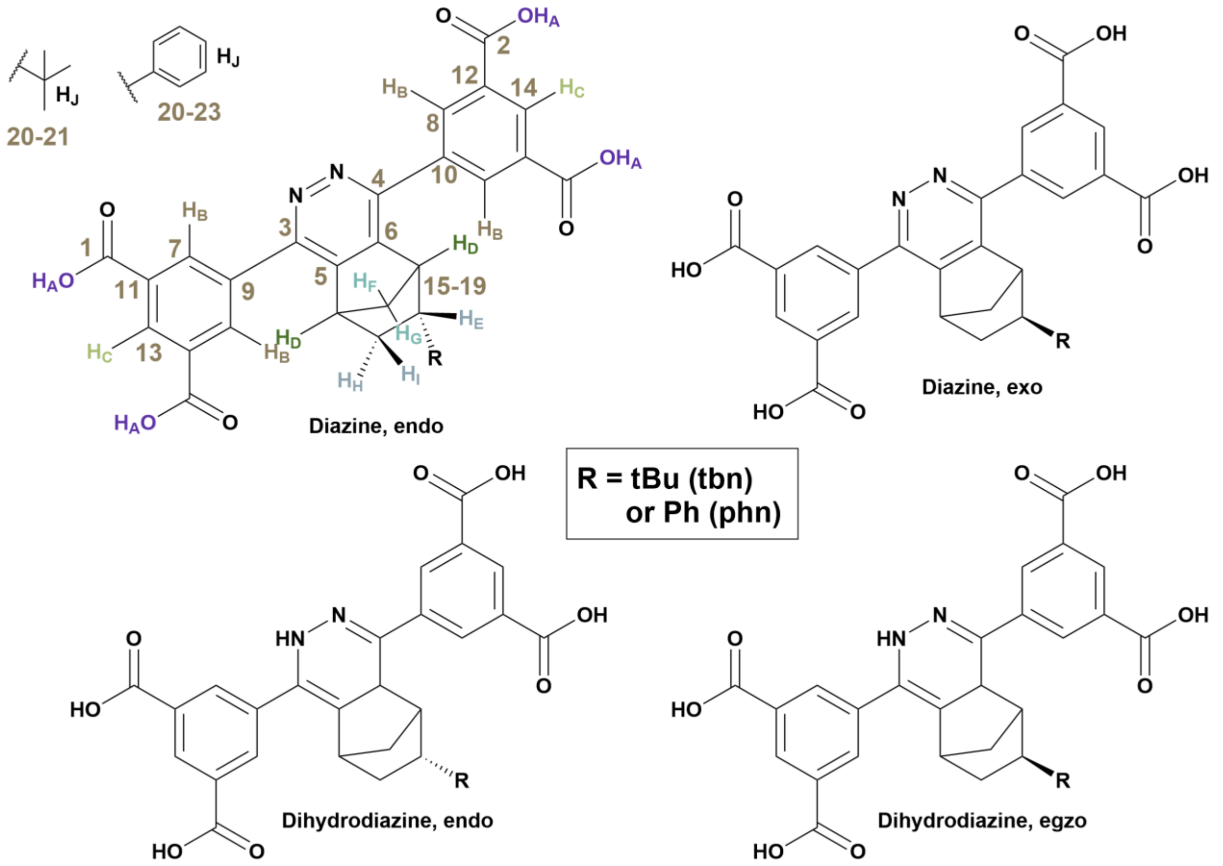

Figure S27. Tags assignment to hydrogen and carbon atoms for H_4tztc-tbn and -phn ligands (top, left). The structures of the side products (dihydrodiazine and *exo* isomers), marked with asterisks on the following spectra.

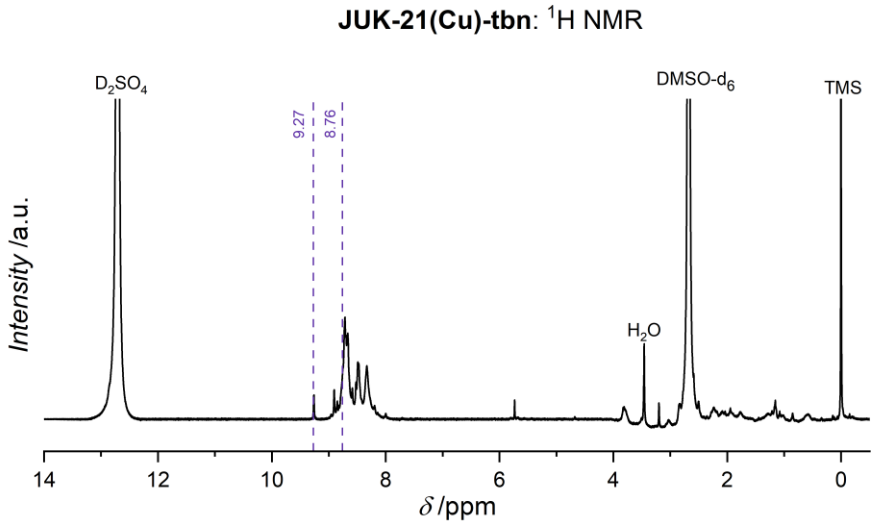

Figure S28. ^{1}H NMR spectra of JUK-21(Cu)-tbn after direct digestion in D_2SO_4/DMSO-d_6.

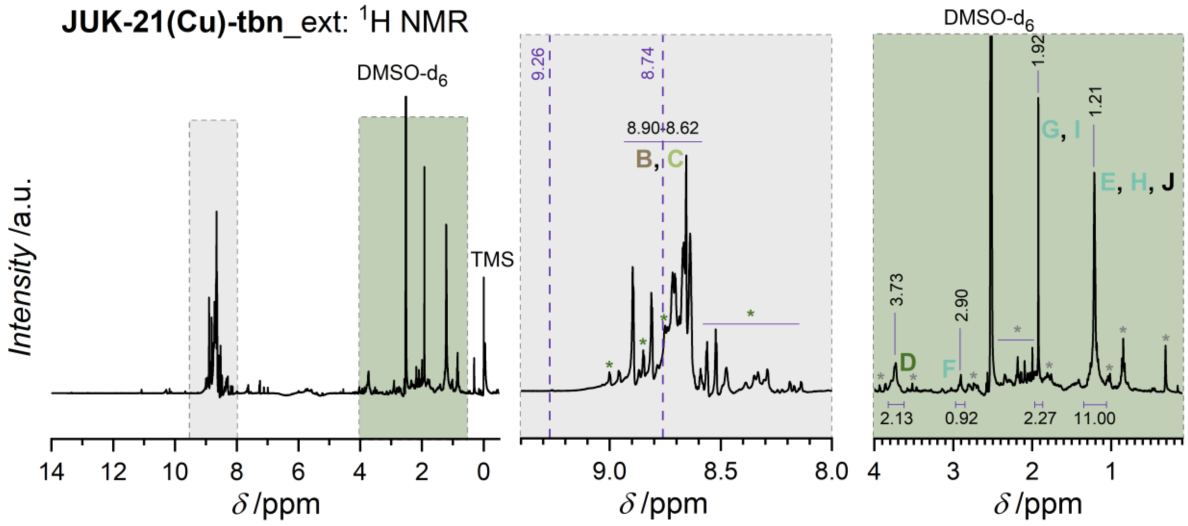

Figure S29. ^{1}H NMR spectra of H_4tztc-tbn after digestion and extraction of JUK-21(Cu)-tbn.

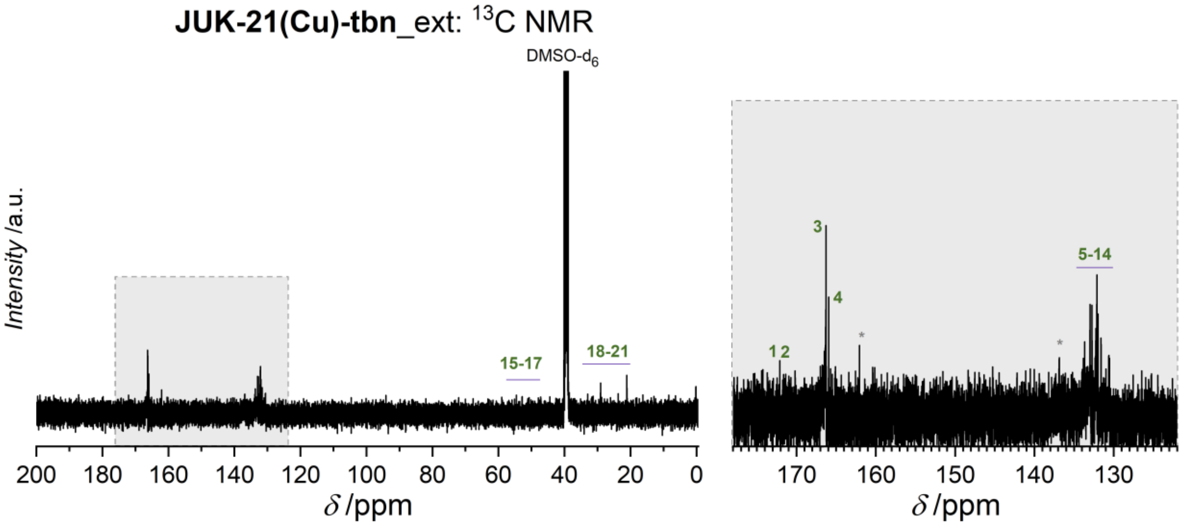

Figure S30. ^{13}C NMR spectra of H_4tztc-tbn after digestion and extraction of JUK-21(Cu)-tbn.

H_4tztc-tbn ^{13}C NMR peaks (tags from Figure S27): **1**: 172.10, **2**: 171.65, **3**: 166.28, **4**: 165.94, **5**: 133.83, **6**: 133.66, **7**: 132.99, **8**: 132.74, **9**: 132.12, **10**: 131.98, **11**: 131.60, **12**: 131.29, **13**: 130.64, **14**: 130.55, **15**: 54.57, **16**: 52.81, **17**: 50.73, **18**: 32.97, **19**: 29.07, **20**: 23.70, **21**: 21.12.

Comments to spectra for JUK-21(Cu)-tbn:

For both H_4tztc-tbn and -phn, the difficulty in interpreting the spectra is caused by the presence of at least 4 different product forms (Figure S27). In each case, aromatic-*endo* is the main product, but the presence of the other three makes interpretation of the spectra difficult (marked with asterisks). For the spectrum in Figure S28, small residue of unreacted ligand can be seen (quantitative analysis in Section S5.3. Calculation of iEDDA reaction yield). For the spectrum in Figure S29, the dominant peaks of the main product can be seen (assignment in the figure).

The aliphatic peaks are poorly visible on the ^{13}C NMR spectrum, as many of these carbon atoms are tertiary and are “diluted” by the presence of other forms.

JUK-21(Cu)-phn

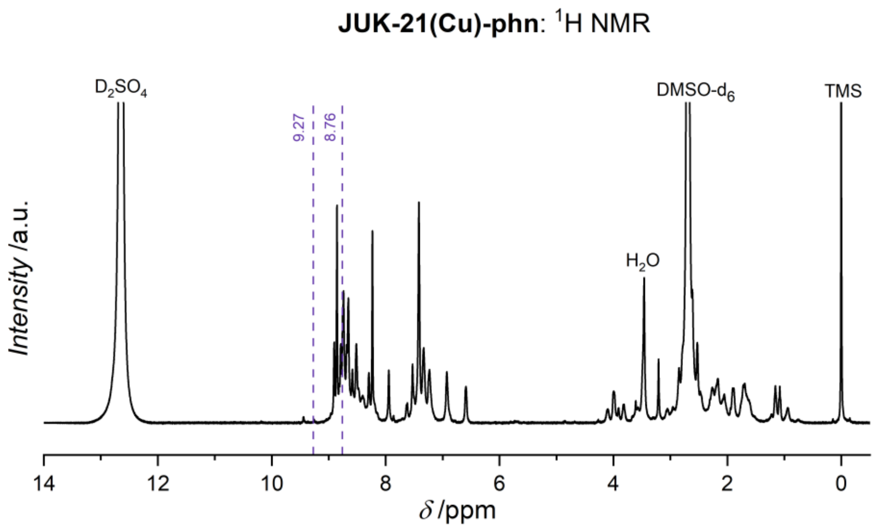

Figure S31. ^{1}H NMR spectra of JUK-21(Cu)-phn after direct digestion in D_2SO_4/DMSO-d_6.

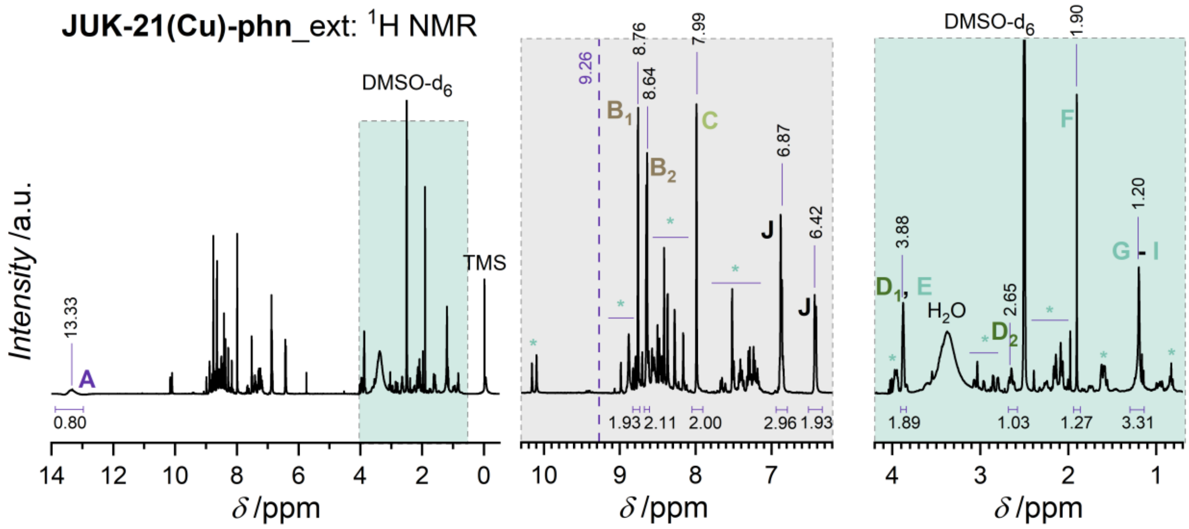

Figure S32. ^{1}H NMR spectra of H_4tztc-phn after digestion and extraction of JUK-21(Cu)-phn.

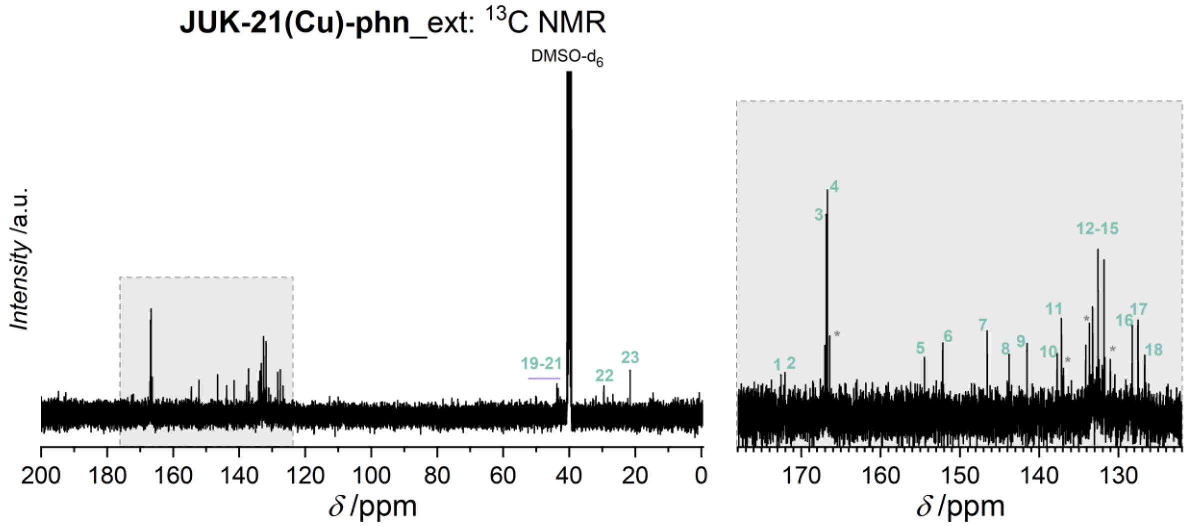

Figure S33. ^{13}C NMR spectra of H_4tztc-phn after digestion and extraction of JUK-21(Cu)-phn.

H_4tztc-phn ^{13}C NMR peaks (tags from Figure S27):**1**: 172.56, **2**: 172.05, **3**: 166.85, **4**: 166.71, **5**: 154.48, **6**: 152.16, **7**: 146.55, **8**: 143.80, **9**: 141.53, **10**: 137.74, **11**: 137.19, **12**: 134.12, **13**: 133.67, **14**: 133.27, **15**: 132.58, **16**: 131.83, **17**: 128.27, **18**: 127.53, **19**: 43.74, **20**: 43.37, **21**: 42.72, **22**: 29.52, **23**: 21.58.

Comments to spectra for JUK-21(Cu)-phn:

For both H_4tztc-tbn and -phn, the difficulty in interpreting the spectra is caused by the presence of at least 4 different product forms (Figure S27). In each case, aromatic-*endo* is the main product, but the presence of the other three makes interpretation of the spectra difficult. For both proton H_4tztc-phn spectra (Figure S24 and Figure **S32**), it can be seen that the product has no unreacted tetrazine. Peaks in both the aromatic and aliphatic regions are visible, but for the directly digested sample (Figure S31) the broadening of the peaks makes accurate interpretation impossible. For the spectrum registered for the sample after solution and extraction (Figure S32), the signals are much narrower, and the signals of the main product (annotation in the figure) are prominent; moreover, the integrations of these signals correspond to the respective proton groups. A multitude of other signals, marked with asterisks, are apparent, each from different side product forms.

On the carbon spectrum (Figure S33) all 23 peaks are clearly visible, 5 of them in aliphatic part. Peaks of side products are also present, both in aromatic and aliphatic part (marked with asterisks).

JUK-21(Cu)-ode

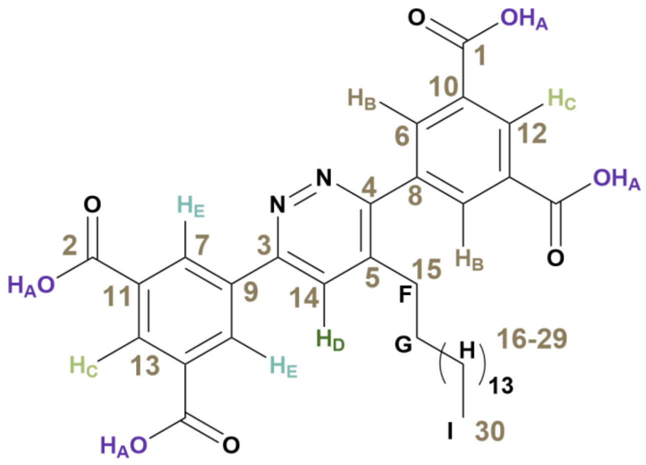

Figure S34. Tags assignment to hydrogen and carbon atoms for H_4tztc-ode ligand.

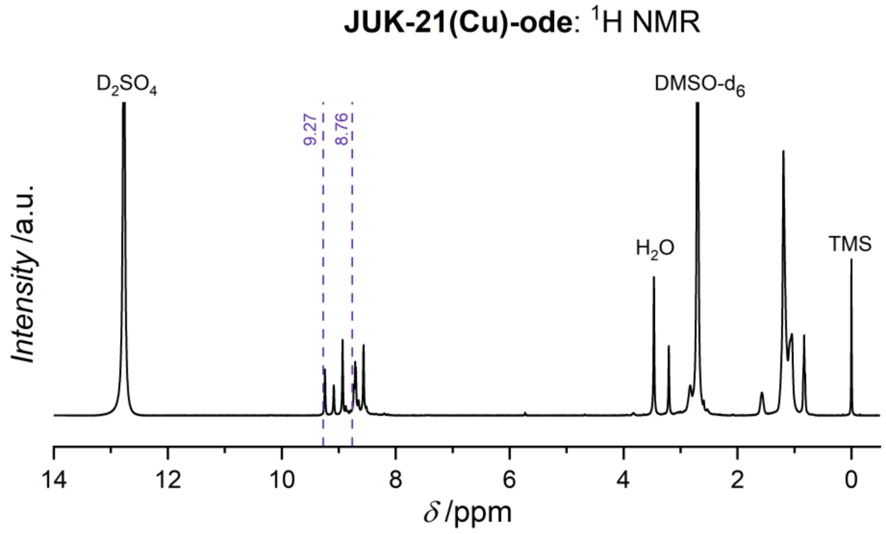

Figure S35. ^{1}H NMR spectra of JUK-21(Cu)-ode after direct digestion in D_2SO_4/DMSO-d_6.

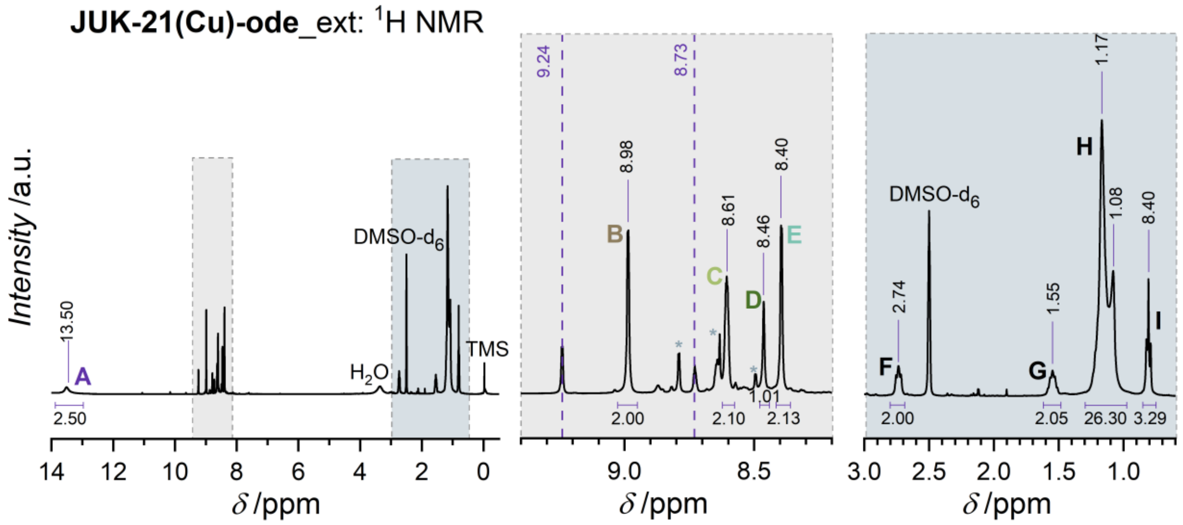

Figure S36. ^{1}H NMR spectra of H_4tztc-ode after digestion and extraction of JUK-21(Cu)-ode.

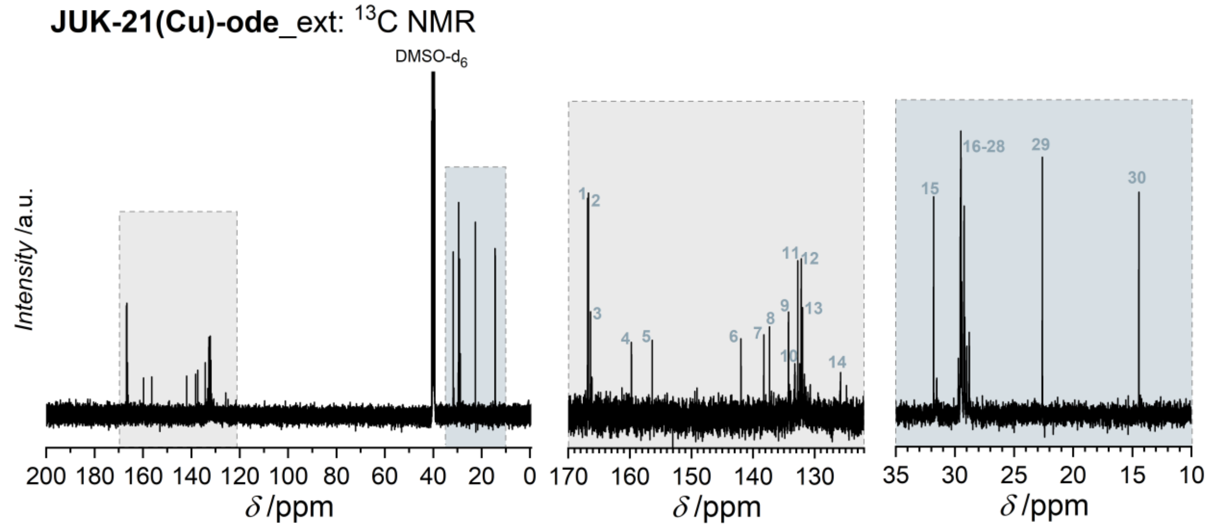

Figure S37. ^{13}C NMR spectra of H_4tztc-ode after digestion and extraction of JUK-21(Cu)-ode.

H_4tztc-ode ^{13}C NMR peaks (tags from Figure S34): **1**: 166.86, **2**: 166.73, **3**: 166.39, **4**: 159.76, **5**: 156.37, **6**: 141.98, **7**: 138.26, **8**: 137.33, **9**: 134.26, **10**: 133.24, **11**: 132.75, **12**: 132.19, **13**: 131.98, **14**: 125.80, **15**: 29.71, **16-22**: 29.53-29.49, **23**: 29.42, **24**: 29.39, **25**: 29.23, **26**: 29.16, **27**: 28.99, **28**: 28.79, **29**: 22.61, **30**: 14.45.

Comments to spectra for JUK-21(Cu)-ode:

For H_4tztc-ode, an excess of unreacted tetrazine is visible on both proton spectra (Figures Figure **S35** and Figure **S36**). On the basis of the peaks integration for the directly digested sample (Figure S35) in the subsequent Section, the degree of material conversion was calculated. The ^{1}H NMR spectrum obtained for the sample after extraction (Figure S36) clearly shows signals from the main product, with peaks from trace amounts of dihydrodiazine product marked with asterisks. Peaks originating from the aliphatic part group into four multiples, clearly visible.

On the carbon spectrum (Figure S37) all 30 peaks are clearly visible, 16 of them in aliphatic part. Peaks of side products are also virtually invisible.

S5.3. Calculation of iEDDA reaction yield

Direct digestion of JUK-21(Cu)-x samples enables verification of the degree of JUK-21(Cu) material conversion in particular iEDDA reactions. The most accurate estimate is therefore based on a careful analysis of the spectra shown in Figure S20, Figure **S24**, Figure **S28**, Figure **S31** and Figure **S35**.

No peaks are visible for JUK-21(Cu)-sty, -nor and -phn at the 9.27 and 8.76 ppm regions. When combined with the other indications (infrared spectra: Section S6. IR spectra and UV-vis-NIR spectra: Section S7. UV-vis-NIR electron spectroscopy), this gives comprehensive proof of the 100% yield of the iEDDA reaction in these three cases.

For the reactions with tbn and ode dienophiles, peaks of low intensity can be seen at the respective spectra (Figure S28, Figure **S35**) at the 9.27 and 8.76 ppm positions. This indicates incomplete conversion and is consistent with other indications (UV-vis-NIR and IR spectra) and the fact that these reactions require the most severe reaction conditions (cf. Table S1). The conversion rate was calculated for these materials as follows.

For the JUK-21(Cu)-tbn material (Figure S28 data was used), the peaks in the aromatic range are closely spaced and only the peak at 9.27 ppm is well separated. Its integration was taken to be equal to 4.00 (I_{ZT}, corresponds to the four protons of the H_4tztc ligand, compare with Figure S16). To determine the integration originating from the reaction product (considering all its isomers and forms), the integration of the entire 9.0-8.0 ppm range was determined, 2.00 was subtracted from this value (corresponding to the integration of the second H_4tztc peak, which overlaps with the product peak), and a value of 216.83 (I_{tbn}) was thus obtained. Considering that the integration of I_{tbn} includes all the aromatic protons of the H_4tztc-tbn molecule, of which there are 6 (regardless of the form/isomer of the product), the percentage of the product (and at the same time the yield of the reaction) can be calculated using the following formula:

$$\%tbn = \frac{\frac{I_{tbn}}{6}}{\frac{I_{tbn}}{6} + \frac{I_{TZ}}{4}} \cdot 100\% = \frac{\frac{216.83}{6}}{\frac{216.83}{6} + \frac{4.00}{4}} \cdot 100\% \approx \mathbf{97}\%$$

The calculations given above indicate that the conversion rate in this case is very high, and the method for its determination is very sensitive.

For JUK-21(Cu)-ode (Figure S35 data used), the peak originating from H_4tztc is also well separated from the rest (9.27 ppm, I_{TZ}), it is possible to indicate at the same time two separated peaks originating from protons B (8.98 ppm) and E (8.40 ppm) are also well separated from the rest, so the average integration can be determined. Thus, taking into account the number of protons from how many the peak originates, the percentage of the product (and at the same time the yield of the reaction) can be calculated using the following formula:

$$\%ode = \frac{\frac{1}{2} \cdot \left(\frac{I_B}{2} + \frac{I_E}{2}\right)}{\frac{I_{TZ}}{4} + \frac{1}{2} \cdot \left(\frac{I_B}{2} + \frac{I_E}{2}\right)} \cdot 100\% = \frac{\frac{1}{2} \cdot \left(\frac{8.77}{2} + \frac{9.06}{2}\right)}{\frac{4.00}{4} + \frac{1}{2} \cdot \left(\frac{8.77}{2} + \frac{9.06}{2}\right)} \approx \mathbf{82}\%$$

The result given above indicates that only ode did not reach full conversion, which is related to the lowest reactivity of this dienophile, its volume and diffusion limits.

S6. IR spectra

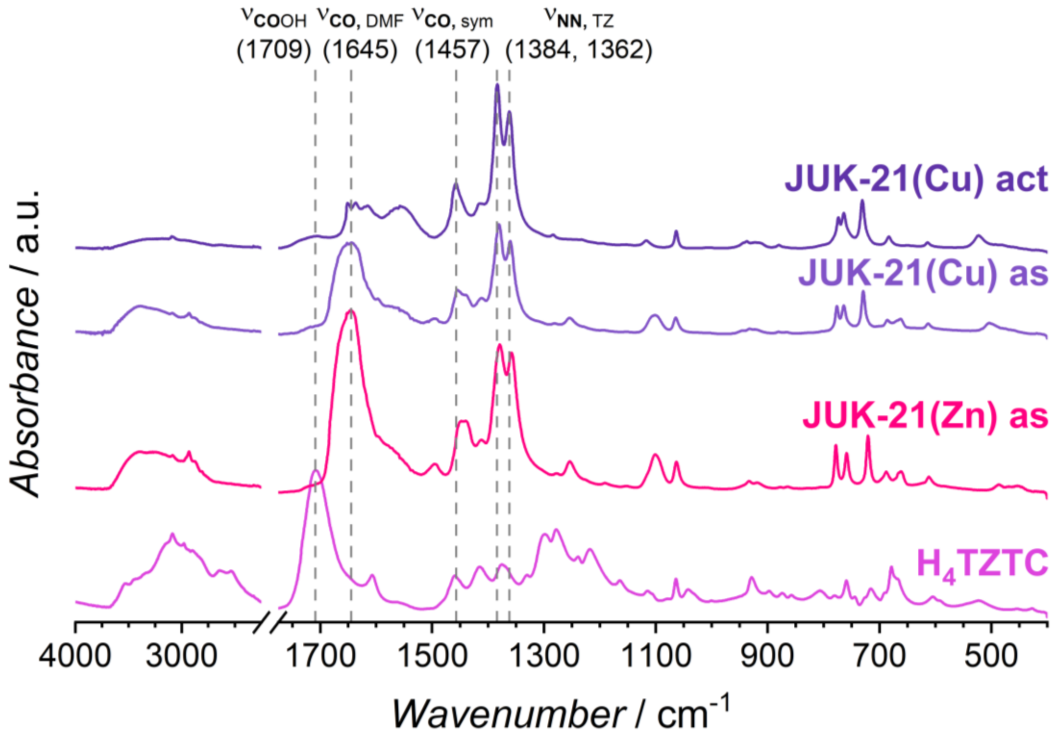

Figure S38. ATR-IR spectra for the H_4tztc ligand precursor, as synthesized (*as*) JUK-21(Zn) and (Cu) materials, and JUK-21(Cu) with guest molecules evacuated (*act*). The band from C=O stretching vibrations (1709 cm^{-1}) indicative of ligand deprotonation, the 1645 cm^{-1} DMF-derived band present for as materials, the 1457 cm^{-1} C-O symmetric stretching band, and the double (1384 and 1362 cm^{-1}) bands from N-N stretching vibrations in the tetrazine ring are marked.

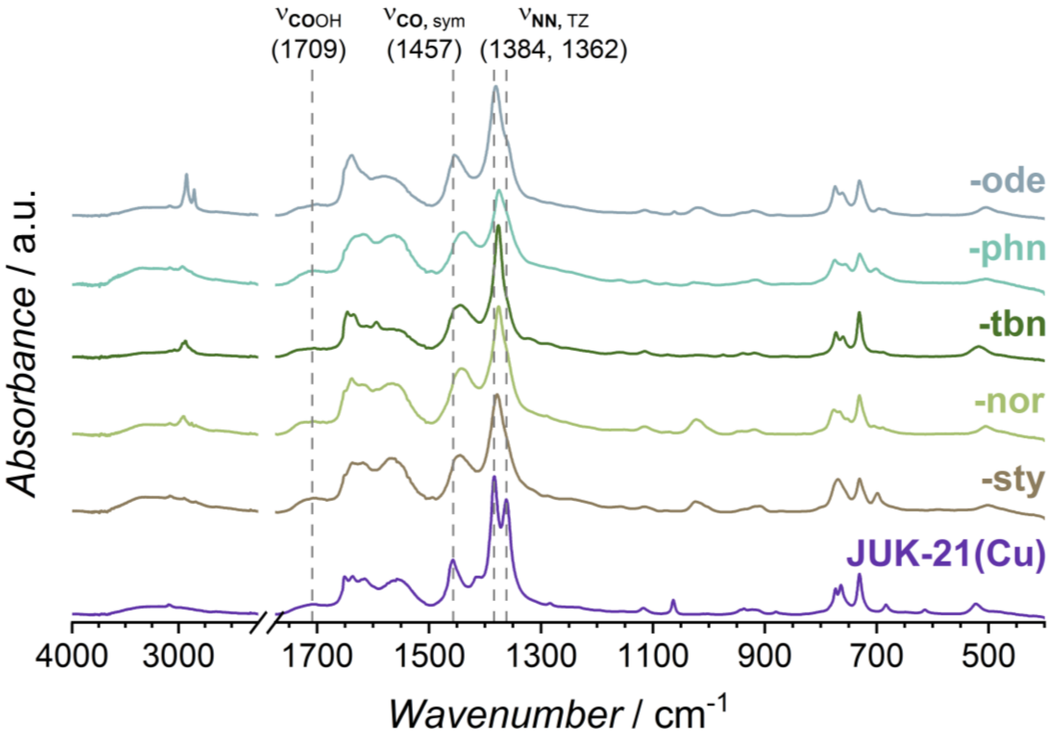

Figure S39. ATR-IR spectra for the series of activated JUK-21(Cu)-x materials. The position where the band from unbound COOH groups would be present (1709 cm^{-1}), the band from symmetric stretching vibrations of the carboxyl group (1457 cm^{-1}), and the 1384-1362 cm^{-1} area where bands from N-N stretching vibrations in the tetrazine ring (JUK-21(Cu)) or diazine rings (iEDDA reaction products) are present are marked - the analysis shown in the next figure.

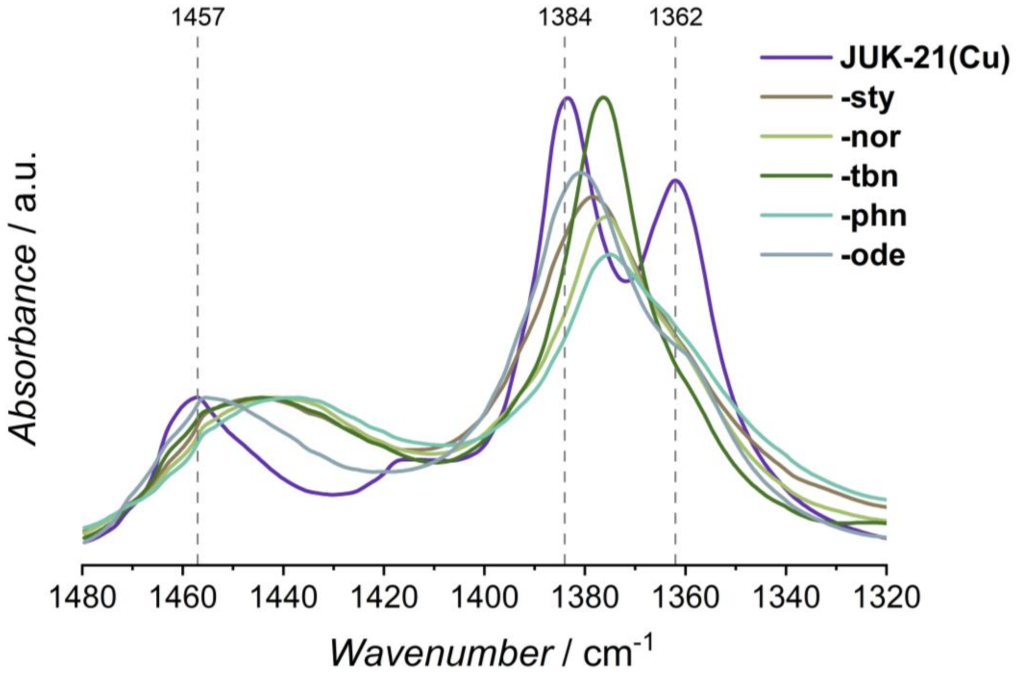

Figure S40. Analysis of 1384-1362 cm^{-1} range of occurrence of bands derived from N-N vibrations in aromatic systems. The series of spectra was normalized to the 1460-1430 cm^{-1} band, which originates from C-O symmetric vibrations, the same across the series. The original doublet band transforms into a single band after each iEDDA reaction, close to the 1375 cm^{-1} value (the 1362 cm^{-1} band disappears entirely).

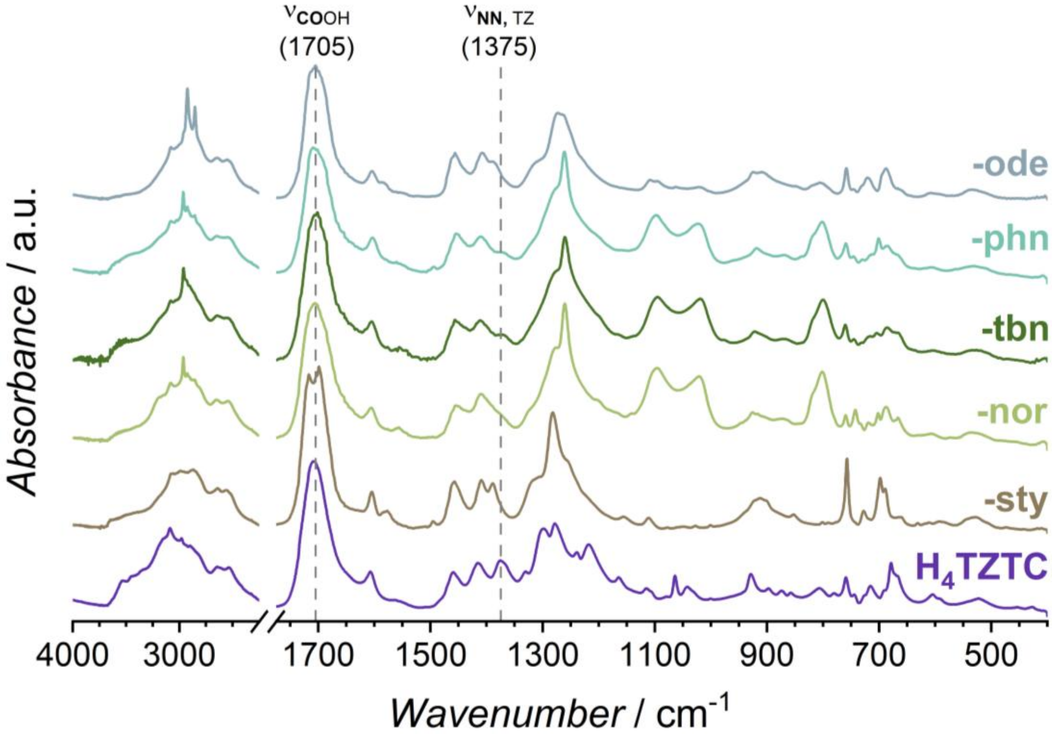

Figure S41. ATR-IR spectra of the H_4tztc ligand precursor and ligands obtained by digestion of each representative from the JUK-21(Cu)-x series (procedure described in Section S5.2. NMR spectra of JUK-21(Cu) after iEDDA). The retention of the band derived from the carboxylic acid group (1705 cm^{-1}) and the disappearance of the band derived from the tetrazine system vibration (1375 cm^{-1}) are marked.

S7. UV-vis-NIR electron spectroscopy

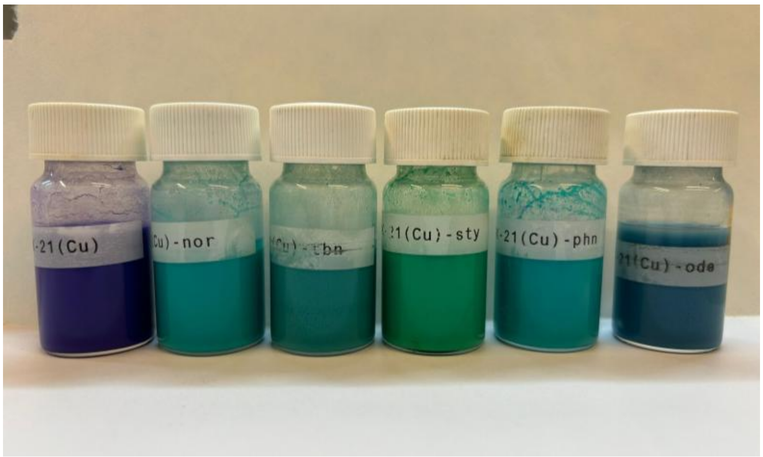

Figure S42. Picture presenting vials containing suspensions of MOF materials from the JUK-21(Cu)-x series as dispersed in methanol. Starting from left: JUK-21(Cu), -nor, -tbn, -sty, -phn and -ode.

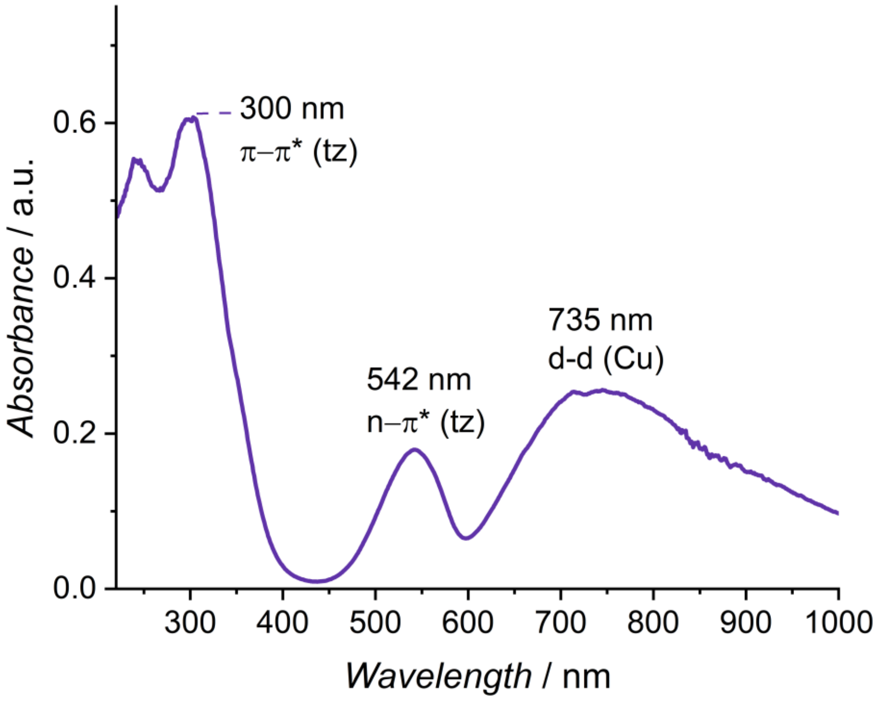

Figure S43. UV-vis-NIR electron spectrum measured for JUK-21(Cu) in solid state after washing with methanol. Three main absorption bands and their origins are indicated, in reference to typical electron transition energies for aryl-substituted s-tetrazine.[22]

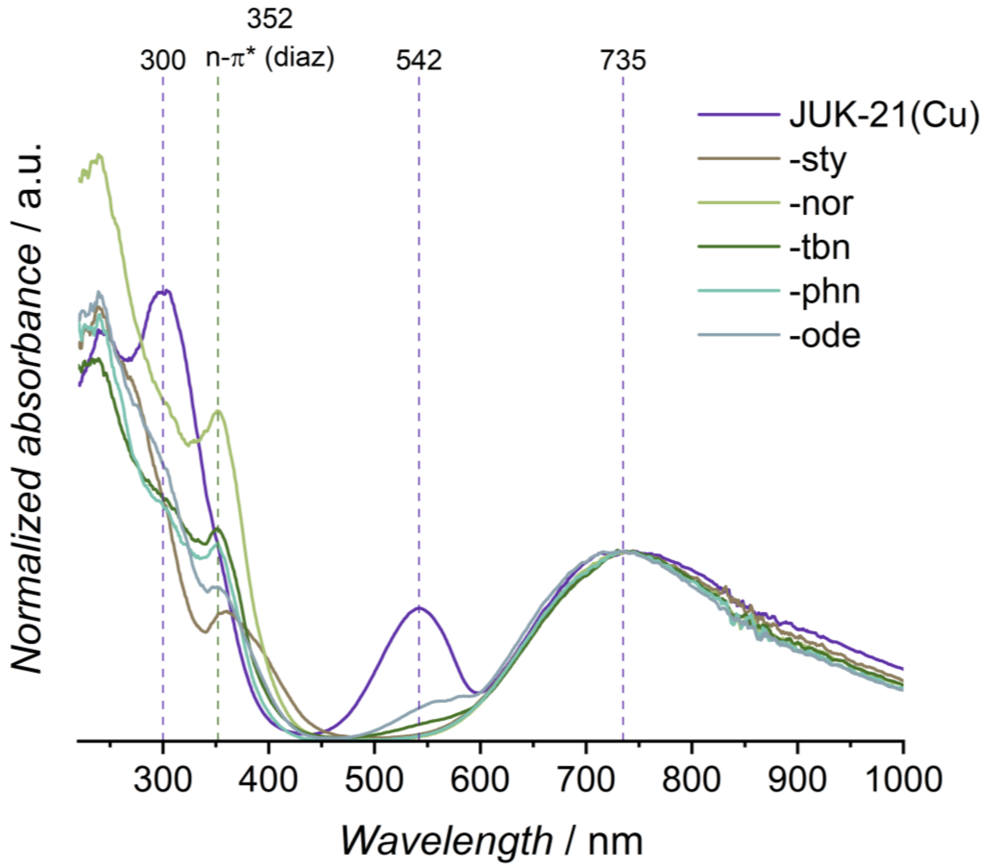

Figure S44. UV-vis-NIR spectra of the JUK-21(Cu)-x series measured in the solid state after methanol washing. Given the reflectance technique used and for direct comparison, the spectra were normalized to the 735 nm absorption band corresponding to the d-d electron transition, originally present in each material at the same intensity. The dashed purple lines indicate the absorption bands of the JUK-21(Cu) material (assignments in Figure S43), while the green highlights the new band that appears exclusively in the materials after the iEDDA reaction. Note that for the JUK-21(Cu)-ode material, a weak yet distinct band from unreacted tetrazine is visible (less than 20%, cf. Section S5.3. Calculation of iEDDA reaction yield).

S8. TG and defect analysis

A series of JUK-21(Cu)-x materials were examined by thermogravimetric analysis. Each sample was studied after washing with methanol, thus the only guest molecules present in the materials' pores were methanol and water. The first series of measurements (Figure S45) was performed in a protective atmosphere (argon) in the range of 25-600 °C to verify the thermal stability of the materials. The second series (Figure S46) was performed in an oxidizing atmosphere (air) in the range of 25-1000 °C to carry out defect analysis (Figure S47).

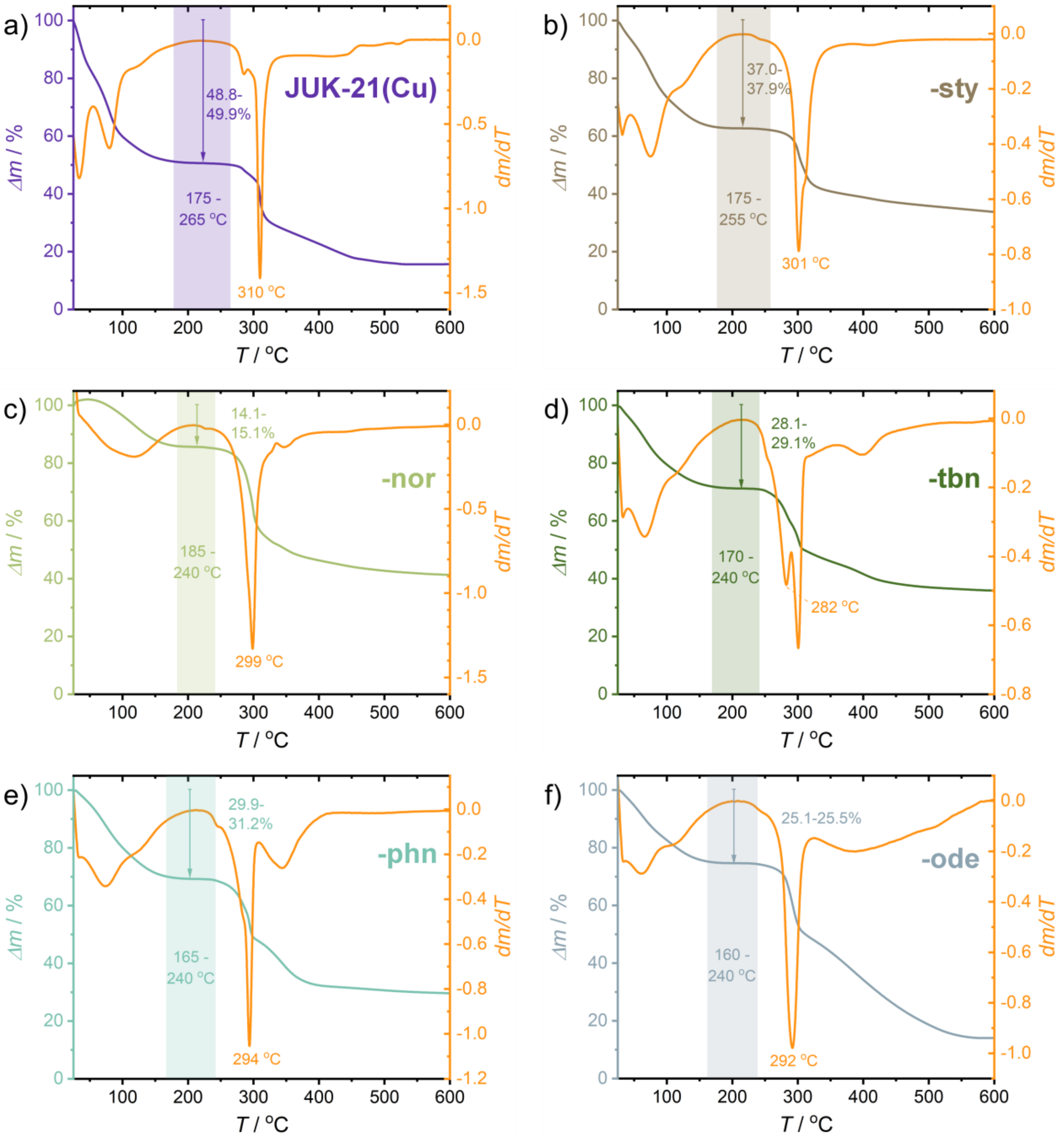

Figure S45. Thermogravimetric curves registered for each tested material under argon flow. The first derivative of *dm/dT* is plotted in orange, allowing analysis of the dynamics of mass loss and indicating the temperature at which full degradation of the material occurs (indicated in the plots). The temperature range at which the material is stable and devoid of guest molecules is shaded on each graph. The experimental mass loss associated with methanol and water molecules desorption is also indicated.

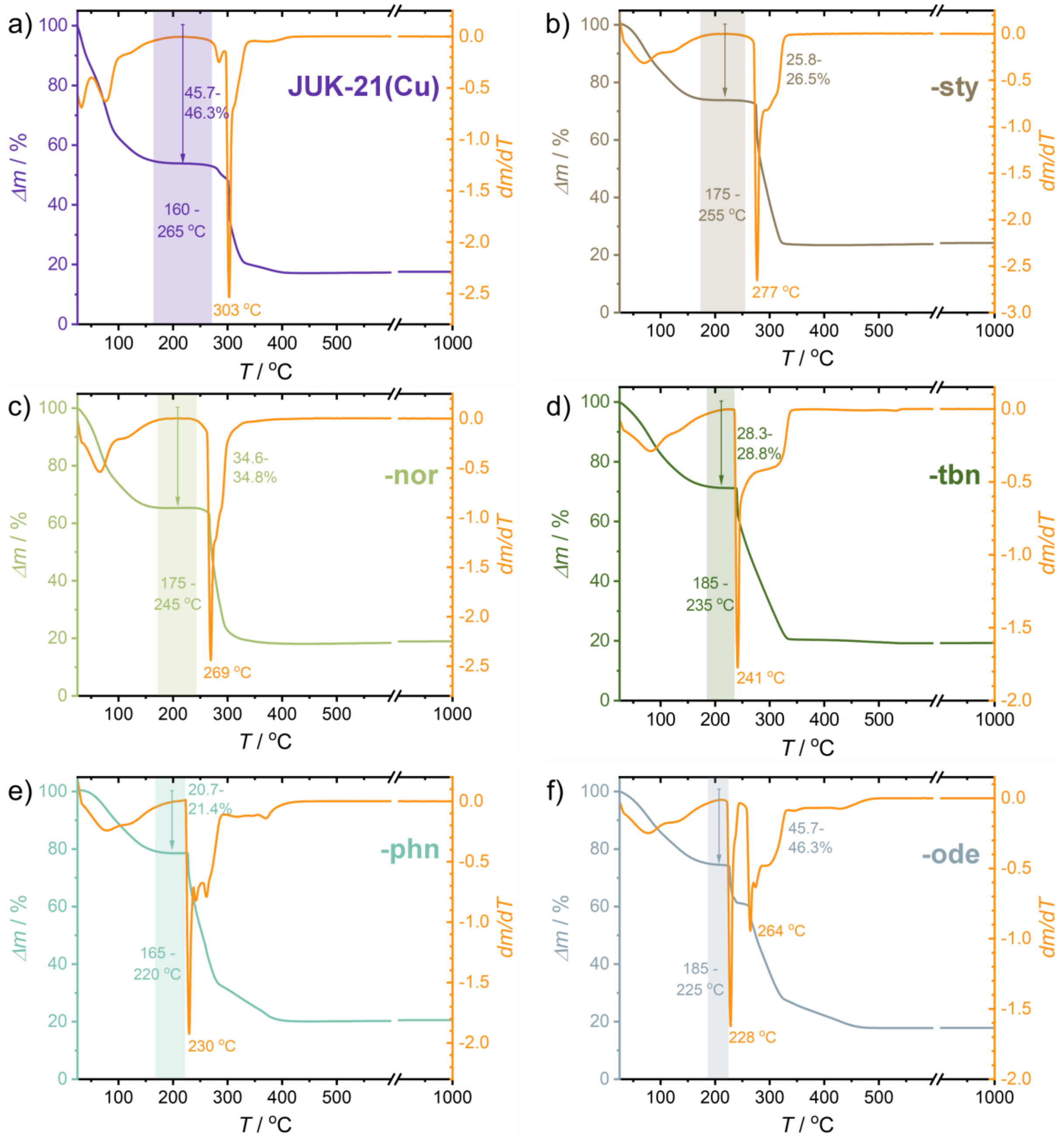

Figure S46. Thermogravimetric curves registered for each tested material under air flow. The first derivative of *dm/dT* is plotted in orange. The temperature range at which the material is stable and devoid of guest molecules is shaded on each graph. The experimental mass loss associated with methanol and water molecules desorption is also indicated.

To calculate the defect concentration of each material, the thermogravimetric curves in Figure S46 were presented in a different way. The final mass of the sample (in the air flow should be pure CuO) was taken as 100%, the entire curve was normalized to this value and presented as Figure S47.

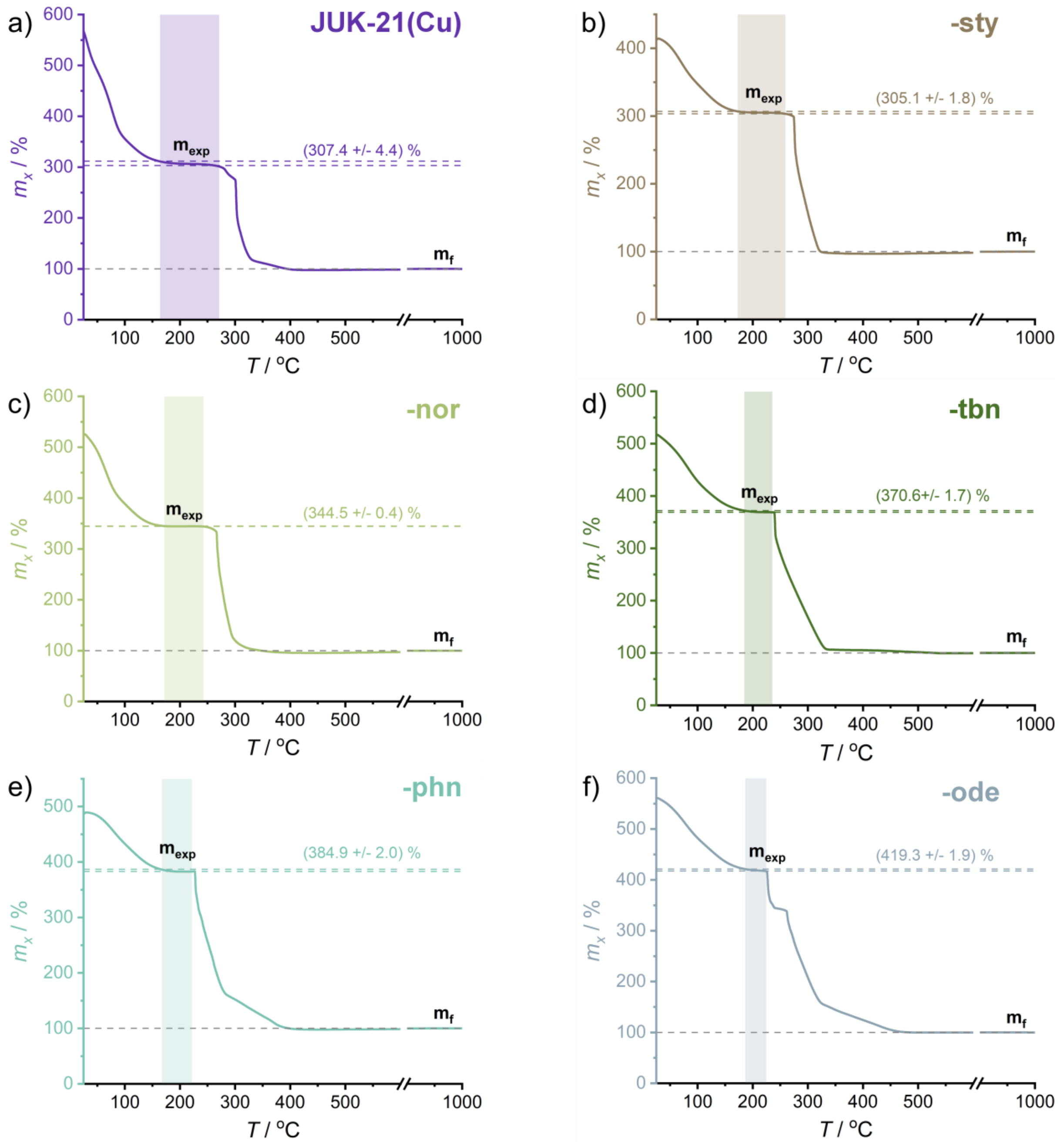

Figure S47. Thermogravimetric curves registered in the air flow, determined by normalizing to the final mass (**m_f**) of the sample, defined as 100%. The range in which the experimental mass of activated MOF (**m_{exp}**) was determined was obtained from Figure S46, the mass was expressed as an averaged percentage of the final mass including the deviation, and marked on the graph.

The theoretical molar mass of the activated MOF material was determined by assuming an undefected composition of [Cu_2(tztc-x)]. Taking into account the incomplete conversion of the starting material with the ode dienophile, the composition of this material was determined using the results presented in Section S5.3. Calculation of iEDDA reaction yield as [Cu_2(tztc-ode)$_{0.82}$(tztc)$_{0.18}$] and based on this, the molar mass was determined. The molar masses calculated in this way were gathered in Table S4.

Taking the molar mass of CuO to be equal to 79.55 g/mol, the molar mass of each activated MOF was calculated using the equation:

$$M_{exp} = 2 \cdot M_{CuO} \cdot \frac{m_{exp}}{m_f} = \left(159.1 \cdot \frac{m_{exp}}{100\%}\right)\frac{g}{mol}$$

The experimental molar masses of the activated MOF materials calculated in this way are listed in Table S4.

To determine the percentage defect in the ligand content for each material, the difference between the theoretical molar mass and the experimental molar mass was divided by the molar mass of the missing ligand (Table S4 lists these respective masses). For the JUK-21(Cu)-ode material, the weighted average mass of $tztc^{4-}$ and tztc-ode^{4-} was taken. The defect degree (%D) calculated in this way (equation below), assuming only missing linker defects, is placed in Table S4.

$$\%D = \frac{M_t - M_{exp}}{M_{(TZTC-x)^{4-}}} \cdot 100\%$$

Table S4. Calculations of the defect degree of each JUK-21(Cu)-x material.

Material	Assumed ideal composition		M_t (g/mol)	M_{exp} (g/mol)	$M_{(tztc-x)}^{4-}$	%D (%)
**JUK-21(Cu)**	[Cu_2(tztc)]	$Cu_2C_{18}N_4O_8H_6$	533.4	489.1 ± 7.0	406.3	**10.9** ± **1.7**
**JUK-21(Cu)-sty**	[Cu_2(tztc-sty)]	$Cu_2C_{26}N_2O_8H_{12}$	607.5	485.4 ± 2.9	480.4	**25.4** ± **0.6**
**JUK-21(Cu)-nor**	[Cu_2(tztc-nor)]	$Cu_2C_{25}N_2O_8H_{14}$	597.5	548.1 ± 0.6	470.4	**10.5** ± **0.1**
**JUK-21(Cu)-tbn**	[Cu_2(tztc-tbn)]	$Cu_2C_{29}N_2O_8H_{22}$	653.6	589.6 ± 2.7	526.5	**12.2** ± **0.5**
**JUK-21(Cu)-phn**	[Cu_2(tztc-phn)]	$Cu_2C_{31}N_2O_8H_{18}$	673.6	612.4 ± 3.2	546.5	**11.2** ± **0.6**
**JUK-21(Cu)-ode**	[Cu_2(tztc-ode)$_{0.82}$ (tztc)$_{0.18}$]	$Cu_2C_{32.7}N_{2.4}O_8H_{33.9}$	715.8	667.1 ± 3.0	588.7	**8.3** ± **0.5**

S9. SEM images

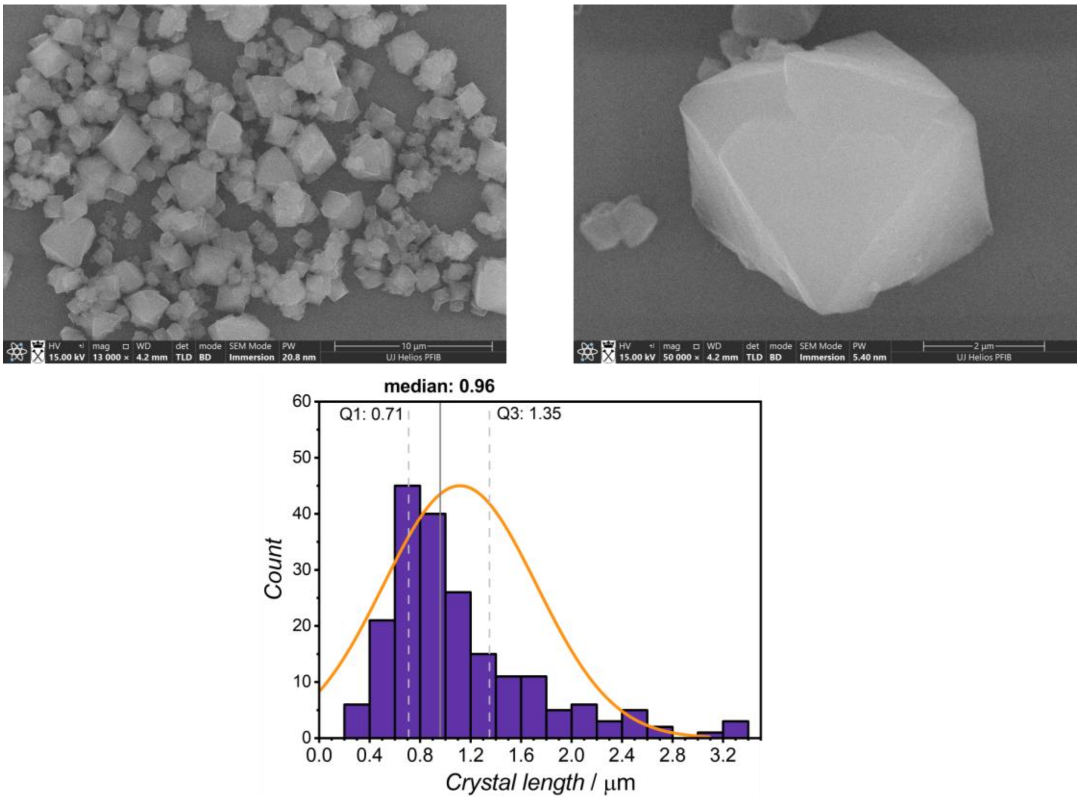

Figure S48. SEM images for JUK-21(Cu) material: A region containing multiple crystallites (top left) and the morphology of a single crystallite (top right). The crystallite size distribution (bottom) is shown with the median, first, and third quartiles indicated. The distribution was analyzed based on a sample of 200 crystallites.

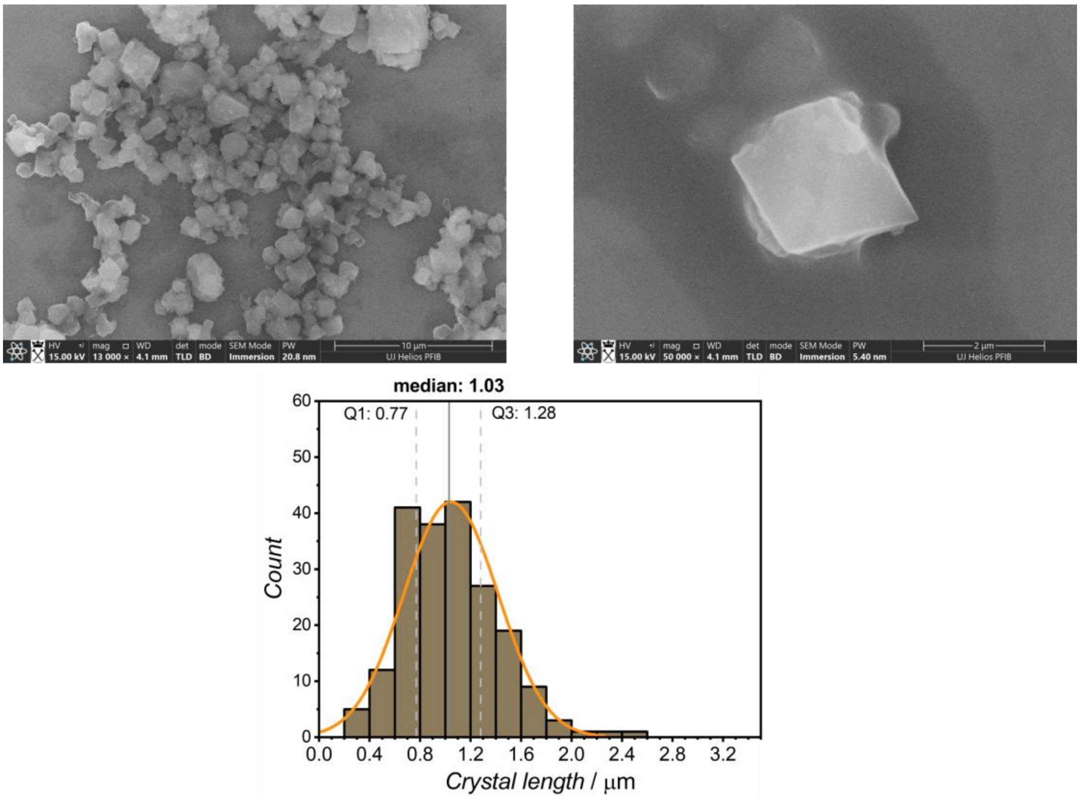

Figure S49. SEM images for JUK-21(Cu)-sty material: A region containing multiple crystallites (top left) and the morphology of a single crystallite (top right). The crystallite size distribution (bottom) is shown with the median, first, and third quartiles indicated. The distribution was analyzed based on a sample of 200 crystallites.

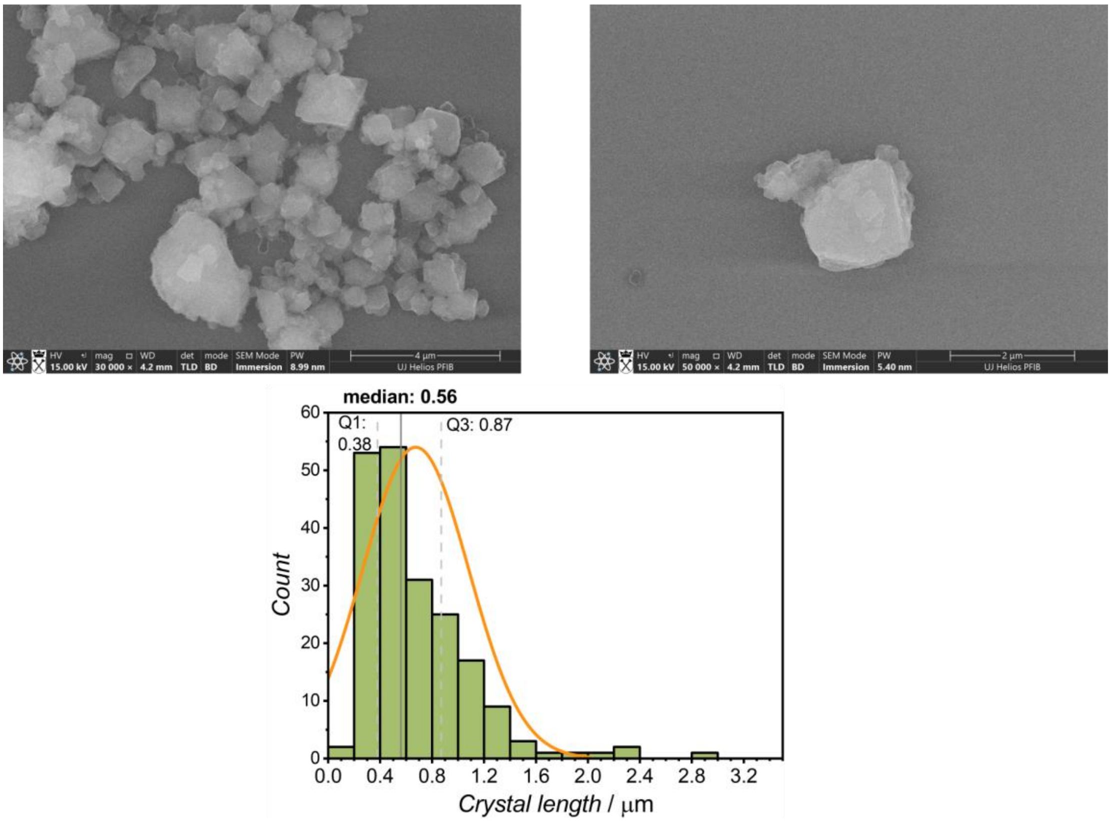

Figure S50. SEM images of the JUK-21(Cu)-nor material: A region containing multiple crystallites (top left) and the morphology of a single crystallite (top right). The crystallite size distribution (bottom) is shown with the median, first, and third quartiles indicated. The distribution was analyzed based on a sample of 200 crystallites.

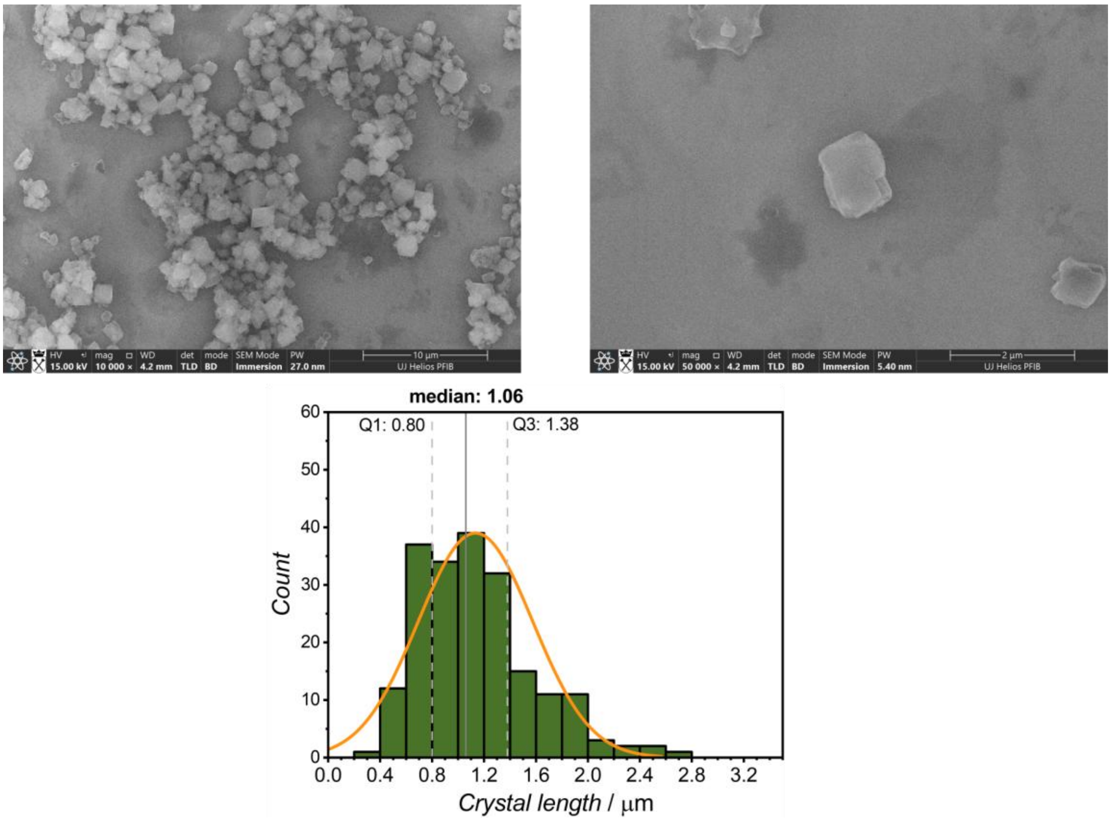

Figure S51. SEM images of the JUK-21(Cu)-tbn material: A region containing multiple crystallites (top left) and the morphology of a single crystallite (top right). The crystallite size distribution (bottom) is shown with the median, first, and third quartiles indicated. The distribution was analyzed based on a sample of 200 crystallites.

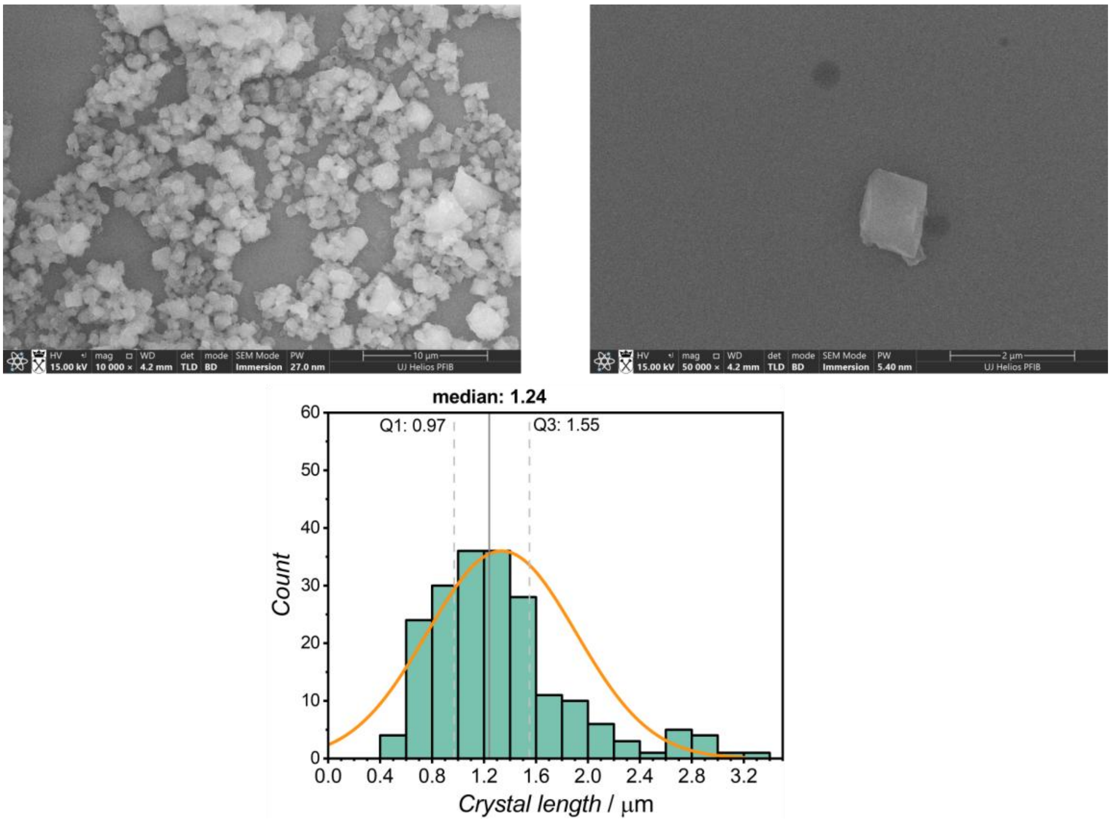

Figure S52. SEM images of the JUK-21(Cu)-phn material: A region containing multiple crystallites (top left) and the morphology of a single crystallite (top right). The crystallite size distribution (bottom) is shown with the median, first, and third quartiles indicated. The distribution was analyzed based on a sample of 200 crystallites.

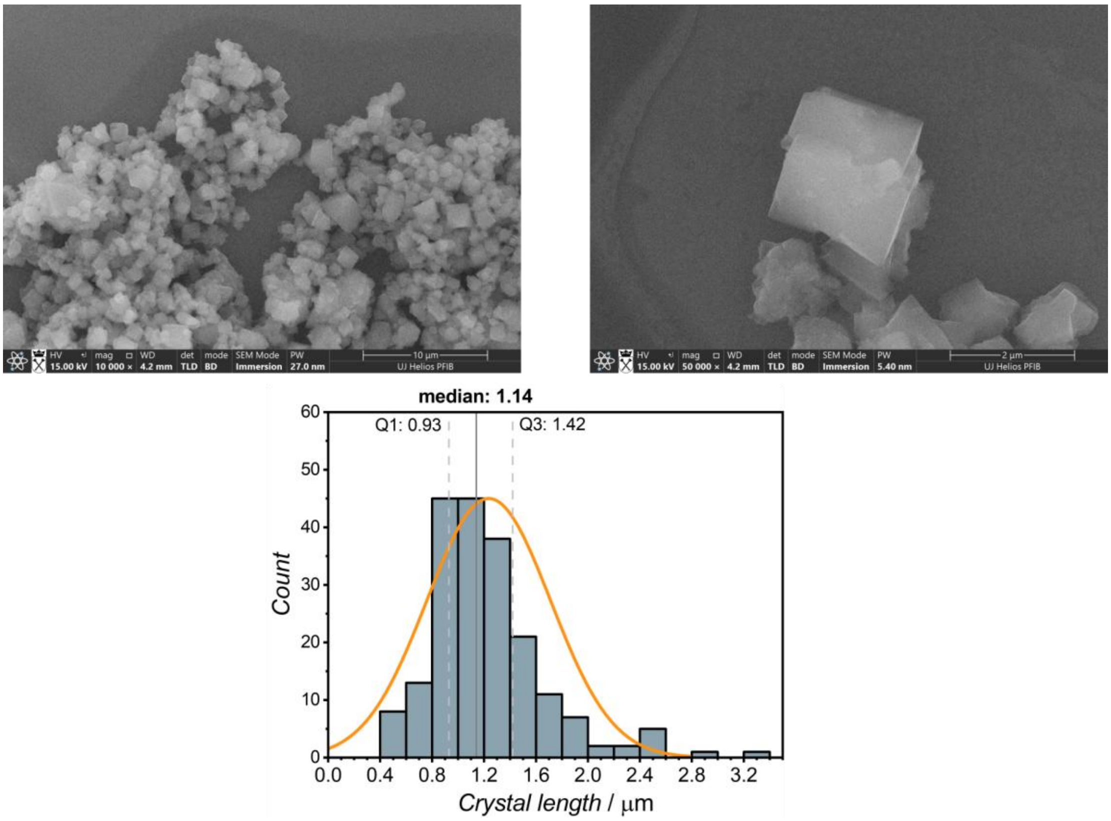

Figure S53. SEM images of the JUK-21(Cu)-ode material: A region containing multiple crystallites (top left) and the morphology of a single crystallite (top right). The crystallite size distribution (bottom) is shown with the median, first, and third quartiles indicated. The distribution was analyzed based on a sample of 200 crystallites.

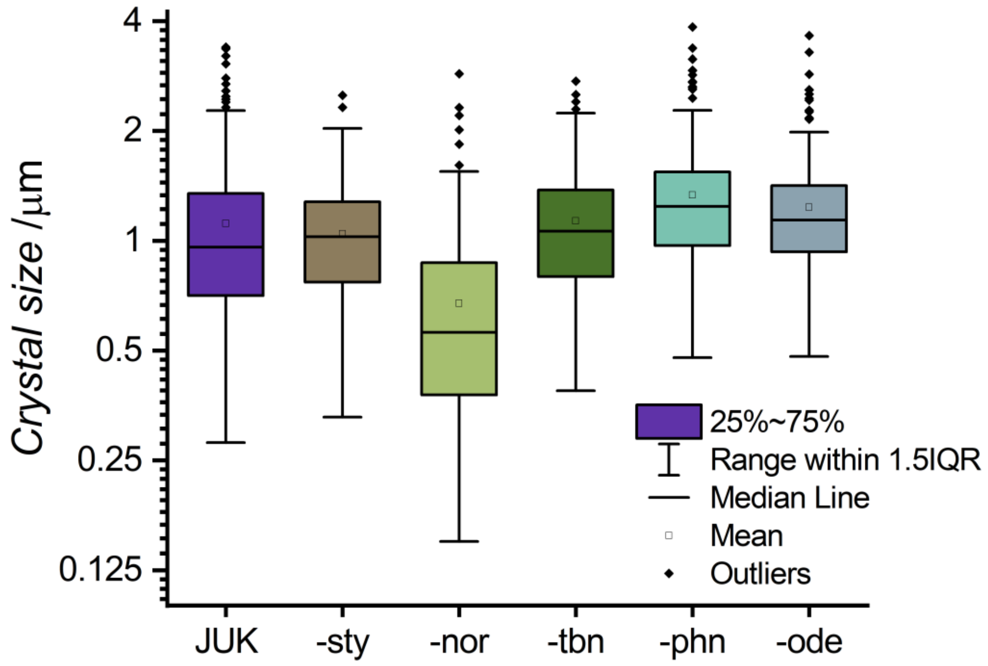

Figure S54. Box plot comparing the crystallite size distribution of each material. The vertical axis is presented on a logarithmic scale. The diagram indicates that JUK-21(Cu)-nor is the only material with a significantly different distribution, which is attributed to crystallite degradation during the iEDDA reaction and material activation.

S10. Nitrogen and carbon dioxide sorption isotherms studies and BET surface area calculations

Brunauer-Emmett-Teller (BET) theory was used to calculate the specific surface area of the materials obtained, using BET Surface Identification (BETSI) software.[6] Extended Roquerol criteria[7] were used, the pressure range optimized to get at least 10 points of linear fit in the BET equation. The pore size distribution (PSD, Figure S55c and Figure S57c) plots were derived from sorption data by NLDFT calculations using a silica cylindrical pore model.

Table S5. BET parameters and sorption data for JUK-21(Cu)-x materials.

Sample	C[a]	Q_m (cm^3/g STP)[a]	S_{BET} (m^2/g)[a]	V_{pore} (cm^3/g)[b]
JUK-21(Cu)	3705	564.5	2458	1.10
JUK-21(Cu)-sty	4394	495.6	2158	0.94
JUK-21(Cu)-nor	5874	366.6	1596	0.66
JUK-21(Cu)-tbn	5181	406.4	1769	0.75
JUK-21(Cu)-phn	5491	368.7	1605	0.69
JUK-21(Cu)-ode	3554	198.9	865.7	0.40

[a] Pressure range used for BET calculation was p/p_0 = 0.002-0.03 or 0.001-0.03 (for JUK-21(Cu)-nor).

[b] Total pore volume calculated from single-point adsorbate uptake at the relative pressure, p/p_0 = 0.95.

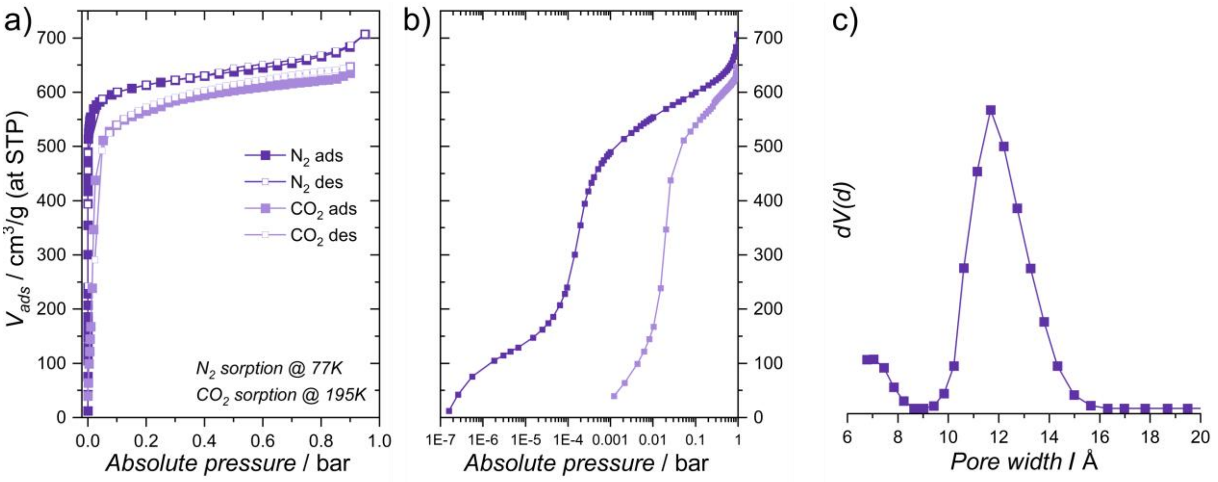

Figure S55. Nitrogen and carbon dioxide sorption isotherms measured at 77K and 195K, respectively for JUK-21(Cu) presented in linear scale (a) and logarithmic scale (b, only adsorption curve plotted for clarity). The pore size distribution plot for nitrogen adsorption on JUK-21(Cu) (c).

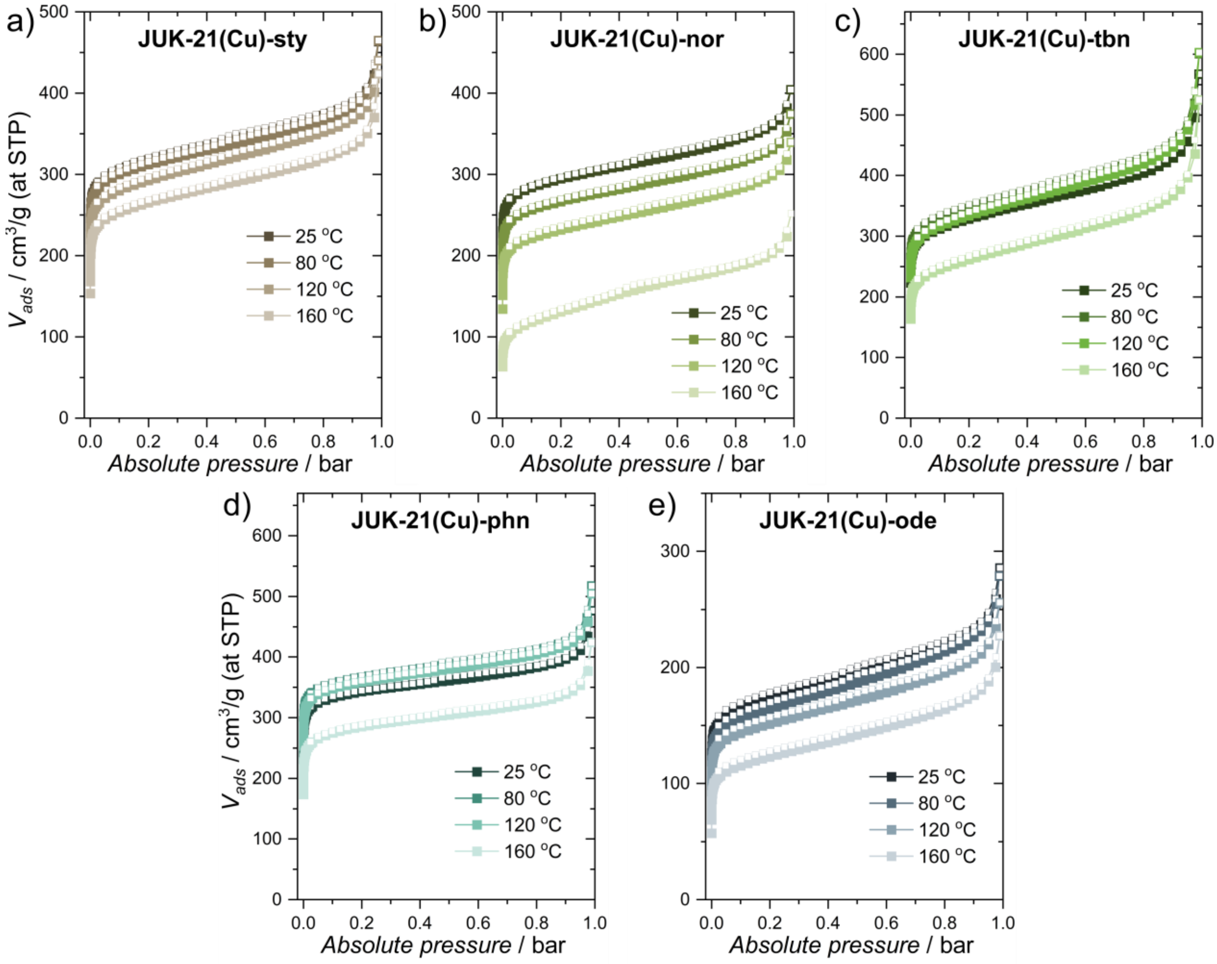

Figure S56. N_2 sorption isotherms for JUK-21(Cu)-x materials obtained by activating these materials at temperatures of 25, 80, 120 and 160°C. The isotherms were measured for one sample, activating it at progressively higher temperatures.

Figure S56 summarizes the result of the study on the optimal activation temperature for each of the modified materials. It should be noted that for the most stable materials, i.e. JUK-21(Cu)-tbn and -phn, the maximum value of sorption capacity occurs for materials activated at 80°C, and raising the temperature to 120°C does not significantly affect the capacity. JUK-21(Cu)-sty and -ode materials have a negligibly small decrease in capacity when the activation temperature is raised from 25 to 80°C, while the effect of temperature is greatest for JUK-21(Cu)-nor, and the initial capacity (with the most mild activation method) is already lower than expected. It should be taken into account that the sorption capacities differ slightly from the values presented in Figure 2 (manuscript) and Figure S54, since the above procedure was carried out without controlling the influence of air at the stage of sample preparation for activation.

In order to obtain adsorption isotherms as close as possible to the real characteristics of the materials, the samples were stored only under solution or in a protective atmosphere. Activation was carried out at the optimal temperature (25°C for JUK-21(Cu)-nor, 80°C for the other materials). Isotherms were recorded from a pressure of 10-7bar, and are illustrated on a linear and logarithmic scale in Figure S57.

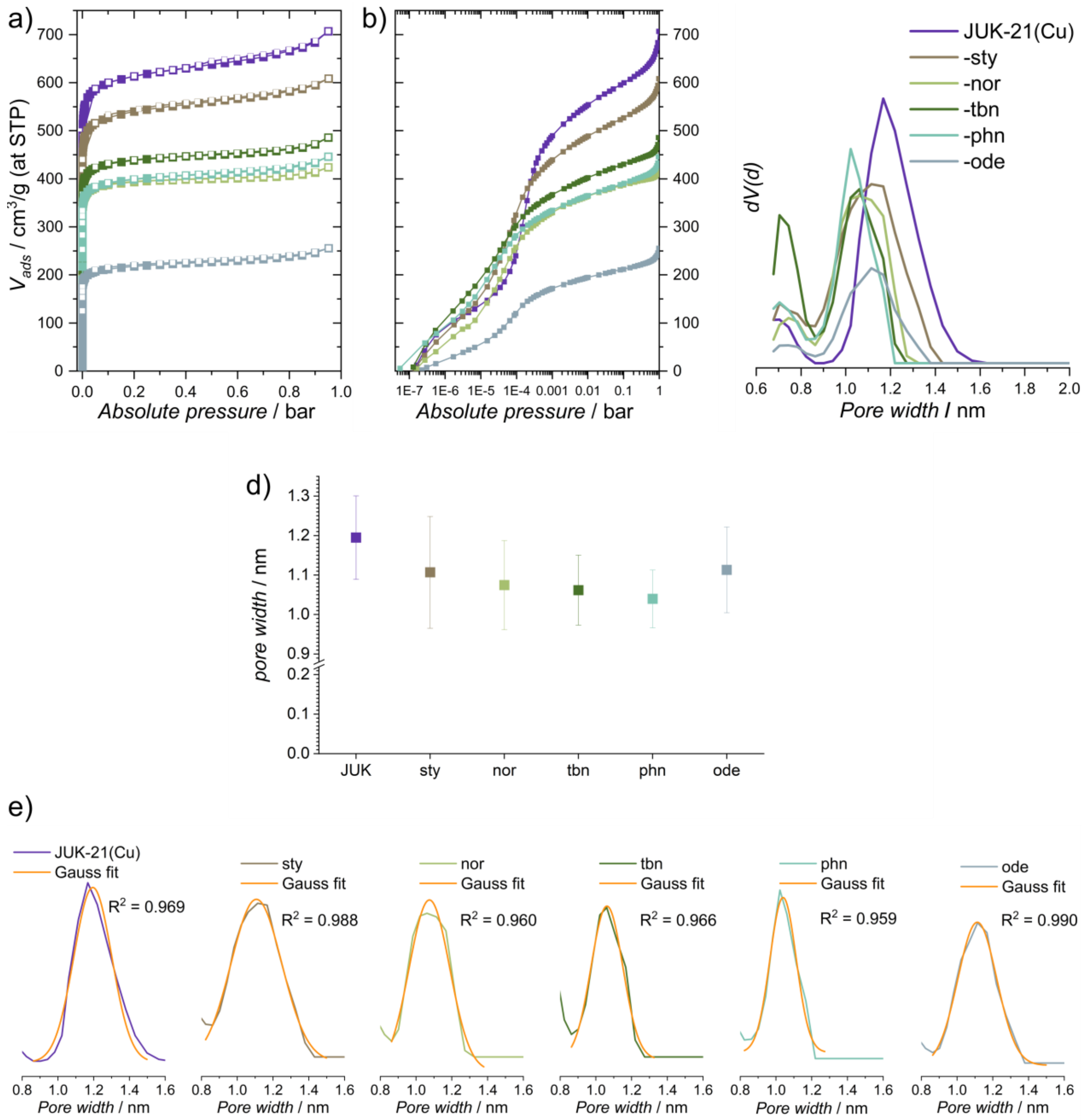

Figure S57. Nitrogen sorption isotherms measured at 77K for JUK-21(Cu)-x materials series presented in linear scale (a) and logarithmic scale (b, only adsorption curve plotted for clarity). The pore size distribution plot for JUK-21(Cu)-x (c). The mean size of the main pore with standard deviation (d) determined by fitting a normal distribution (e) to the main part of the pore size distribution curve in Figure c.

S11. High pressure hydrogen sorption isotherms studies

High-pressure measurements were carried out on a Belsorp HP apparatus in the pressure range of 0-100 bar at 77K. Sample masses in the range of 130-230 mg were used for measurement. Each measurement was associated with the determination of the dead volume of measuring cell, which was determined using measurements of the volume occupied by helium at temperatures of 77 and 298K (empty cell) and 298K for the sample cell.

Figure S58. illustrates the excess isotherms for all materials tested. The adsorbed amount of hydrogen is shown on the left axis as volume per mass of sample, while on the right axis it is shown as the mass of hydrogen in mg per mass of sample. The excess isotherm represents the isotherm that shows the difference between the amount of gas in the cell under the given conditions in the presence and without the adsorbent. Using the conversion through to the molar mass of MOF material, the same isotherms are shown in the Figure S59., presenting the amount adsorbed per mole of Cu_2 unit.

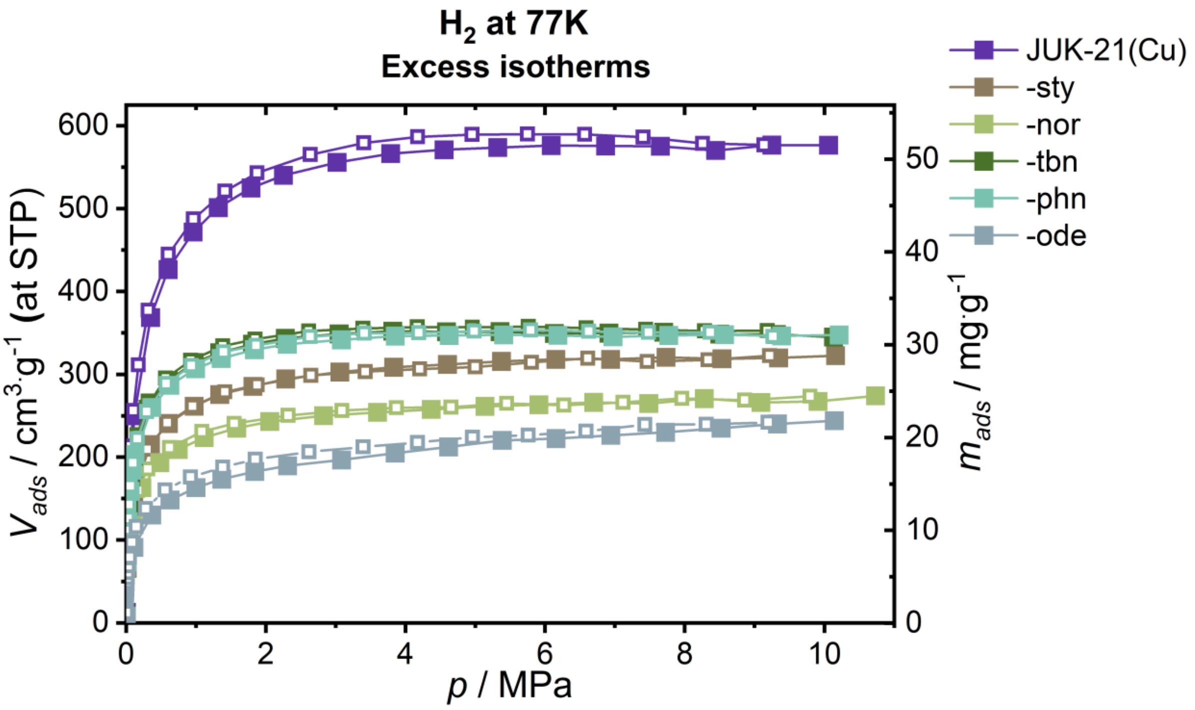

Figure S58. Excess adsorption (closed symbols) and desorption (open symbols) isotherms of hydrogen in the high pressure range (up to 100 bar) at 77K.

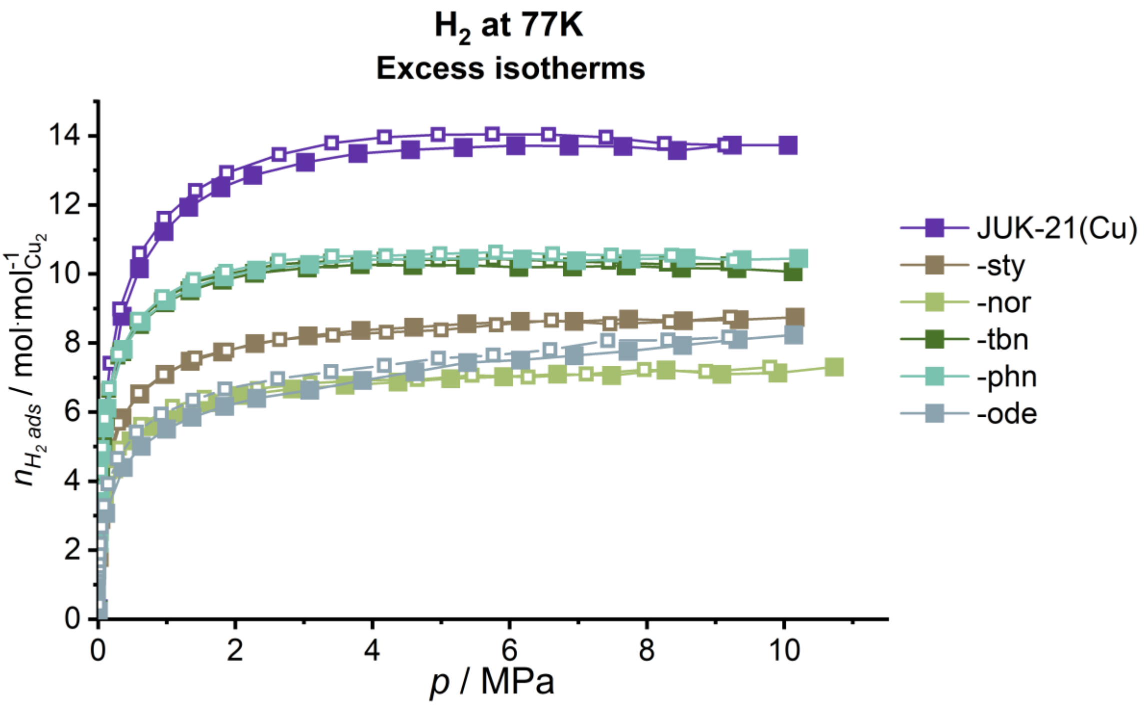

Figure S59. Excess adsorption (closed symbols) and desorption (open symbols) isotherms of hydrogen in the high pressure range (up to 100 bar) at 77K. The amount of gas expressed as moles per mole of Cu_2 unit.

In the range studied, there is no full saturation of the free space (analogy of a liquid in a pore) and the gas present in the pores is incompressible. For this reason, it is necessary to calculate total isotherms, i.e. isotherms with the adsorbed mass increased by an additional mass of hydrogen, which at elevated pressure would occupy the space of the material pores only.[23] This additional factor is marginally small in the low pressure range (1 bar), while above 10 bar its contribution is significant. The comparison of total isotherms in terms of the mass of adsorbed gas and the number of moles per mole of Cu_2 are presented in Figure S60**.** and Figure **S61.**, respectively.

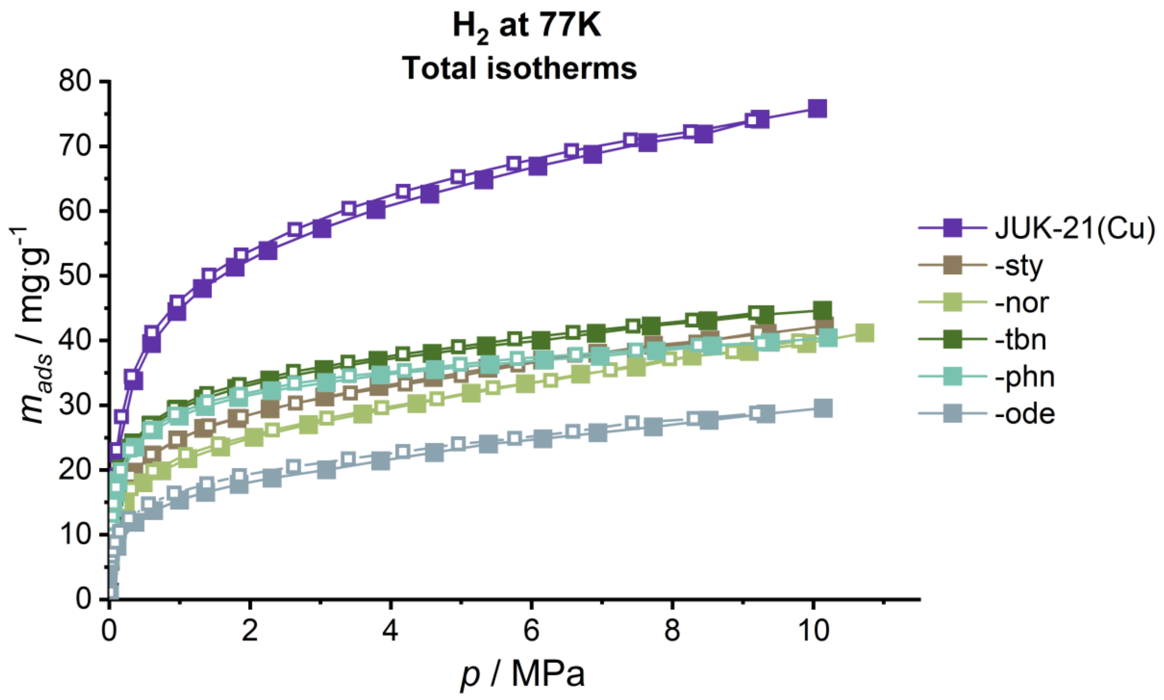

Figure S60. Total adsorption (closed symbols) and desorption (open symbols) isotherms of hydrogen in the high pressure range (up to 100 bar) at 77K. Isotherms present the mass of adsorbed gas per mass of sample.

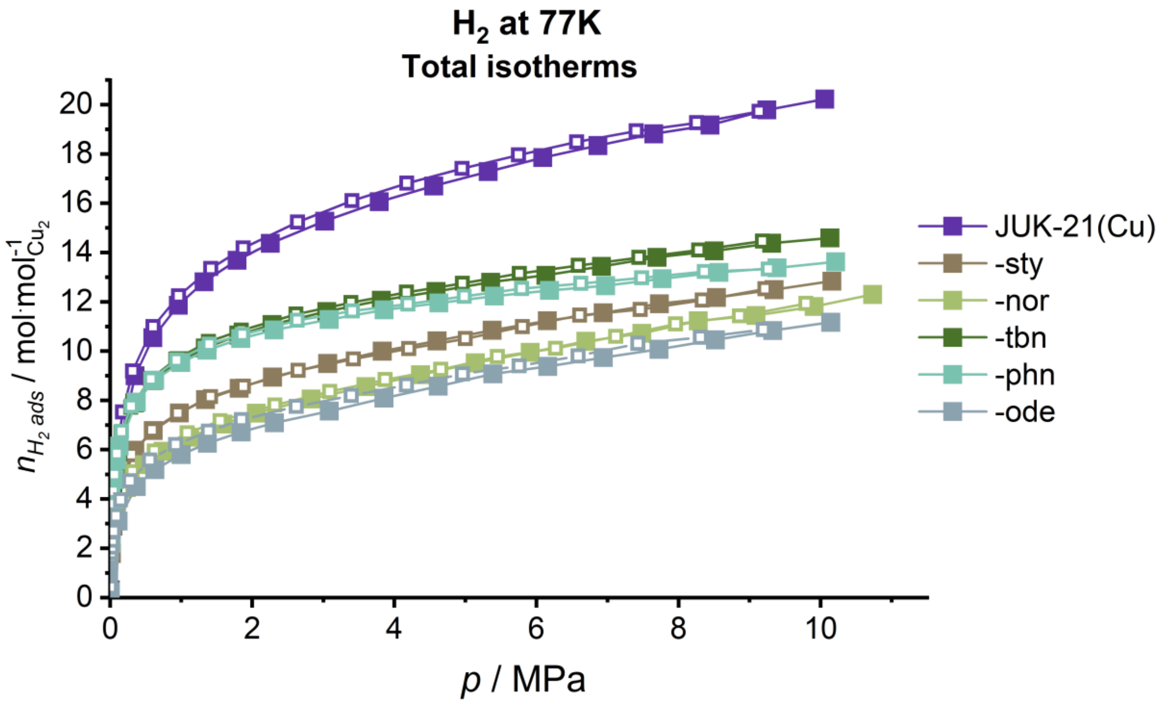

Figure S61. Total adsorption (closed symbols) and desorption (open symbols) isotherms of hydrogen in the high pressure range (up to 100 bar) at 77K. Isotherms present the amount of gas expressed as moles per mole of Cu_2 unit.

S12. Low pressure hydrogen and deuterium sorption isotherms studies

S12.1. Low pressure hydrogen and deuterium adsorption and selectivity calculations by JUK-21(Cu)

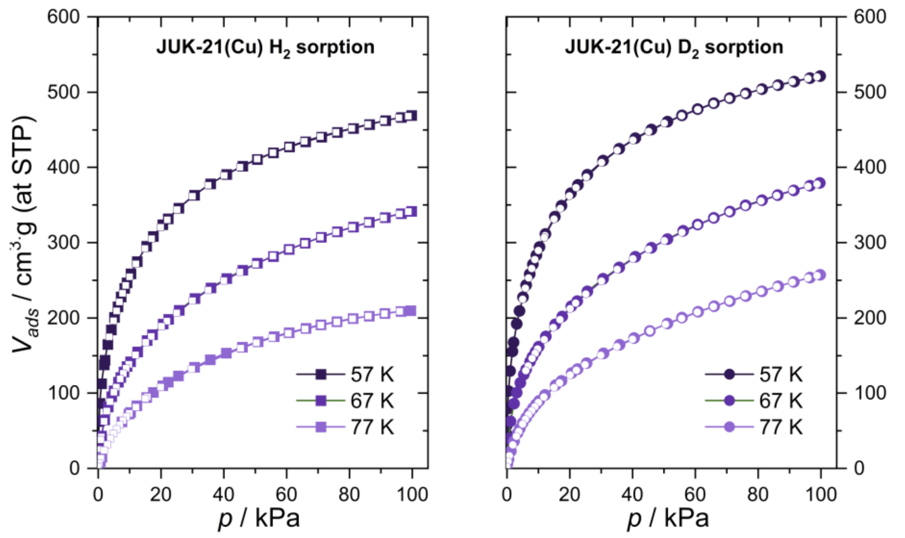

Figure S62. Adsorption (closed symbols) and desorption (open symbols) isotherms of hydrogen (left, squares) and deuterium (right, circles) in the low pressure range at 77, 67 and 57K.

To estimate the selectivity of deuterium adsorption to hydrogen by JUK-21(Cu), the adsorption isotherms of these gases were compared at 77K and the ratio of adsorbed deuterium to hydrogen was plotted (Figure S63**.**).

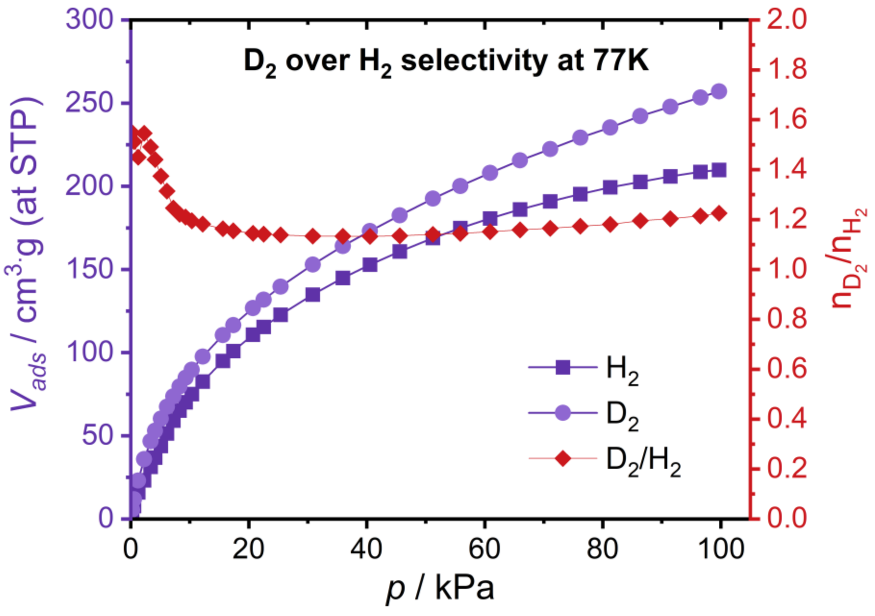

Figure S63. Comparison of hydrogen (squares) and deuterium (circles) adsorption isotherms at 77K (squares) and the ratio of adsorbed deuterium to hydrogen (diamonds).

Subsequently, the isosteric heat of adsorption for hydrogen and deuterium in the JUK-21(Cu) material was estimated. Due to the difficulty of fitting an appropriate model to the collected isotherm data, linear interpolation between experimental points was applied. [24] Based on the resulting plots, the pressures corresponding to a given adsorbed amount of hydrogen or deuterium were extracted. These values were then used to calculate the isosteric heat of adsorption at specific loadings using the Clausius–Clapeyron equation. The associated uncertainty was estimated through the law of uncertainty propagation, taking into account the uncertainty in the slope of the linear interpolation. Figure S64**.** presents the calculated isosteric heat of adsorption per millimole of adsorbate per gram of material.

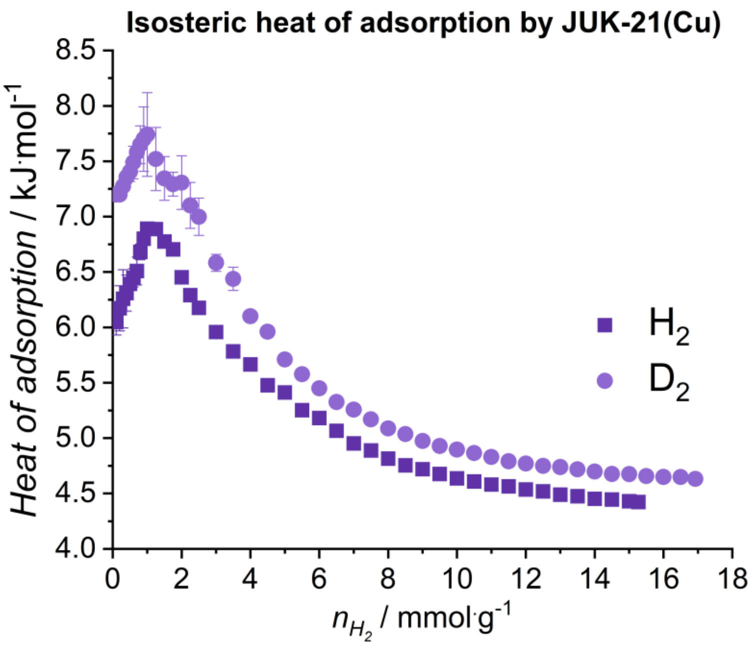

Figure S64. Isosteric heat of adsorption of hydrogen and deuterium for JUK-21(Cu) material.

S12.2. Low pressure hydrogen adsorption by JUK-21(Cu)-x

To analyze the heat of hydrogen adsorption by each of the materials under study, a set of three isotherms of hydrogen adsorption and desorption at 57, 67 and 77 K was collected for the entire series of materials. Figure S65 shows a comparison of isotherms between materials at the two temperatures, while Figure S66 depicts a compilation of a series of three isotherms for each material separately.

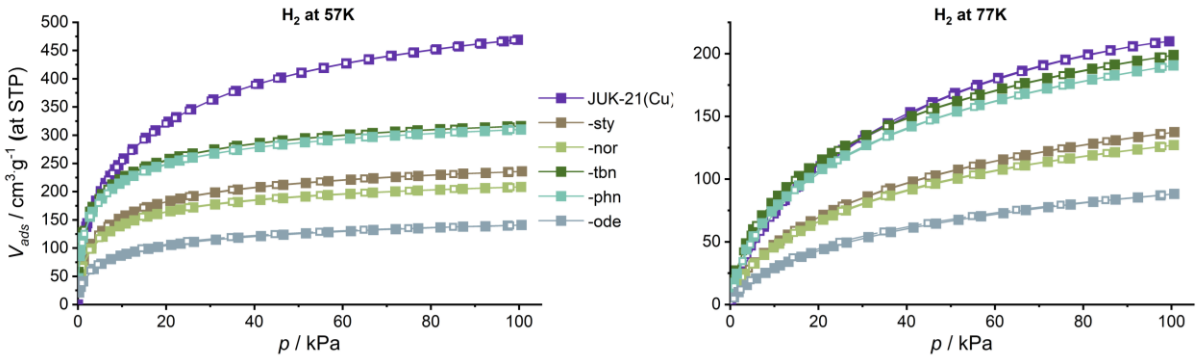

Figure S65. Comparison of adsorption (closed symbols) and desorption (open symbols) isotherms of hydrogen in the low-pressure range at 57K (left) and 77K (right) for all materials studied.

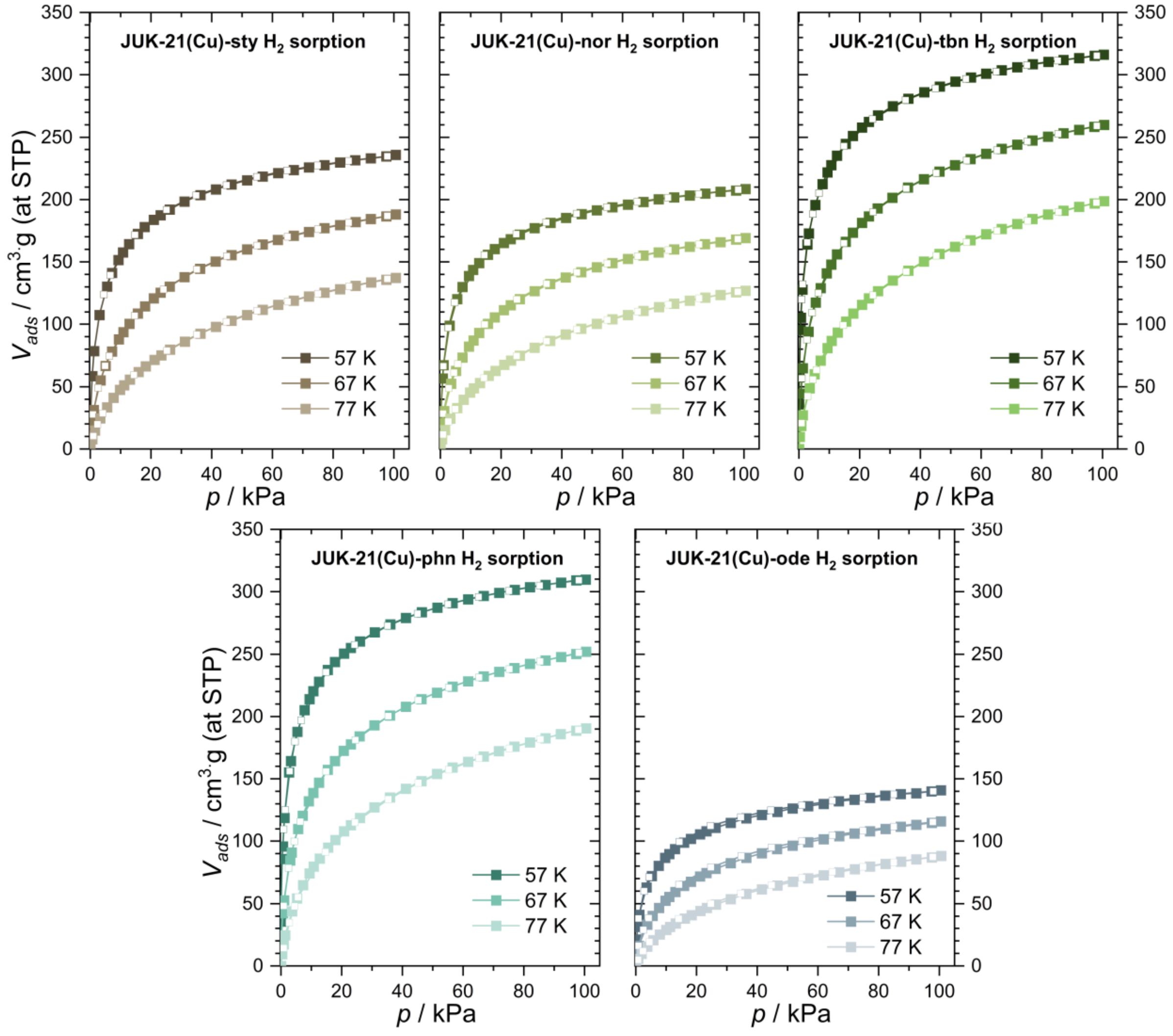

Figure S66. Adsorption (closed symbols) and desorption (open symbols) isotherms of hydrogen in the low-pressure range at 57, 67 and 77K for all tested materials.

S12.3. Isosteric heat of hydrogen adsorption by JUK-21(Cu)-x

Based on the isotherms presented in Figure S66, the isosteric heat of hydrogen adsorption was determined following the same procedure as described for JUK-21(Cu). Due to the lower number of adsorption points collected, the uncertainties – particularly at very low sorption capacities – are relatively high and should be considered in the interpretation. Figure S67 illustrates the relationship between the heat of adsorption and the amount of adsorbed hydrogen for each material, including the associated uncertainties. Figure S68, in turn, presents a combined plot of all datasets (including JUK-21(Cu) from Figure S64) on a single graph, with error bars omitted for clarity.

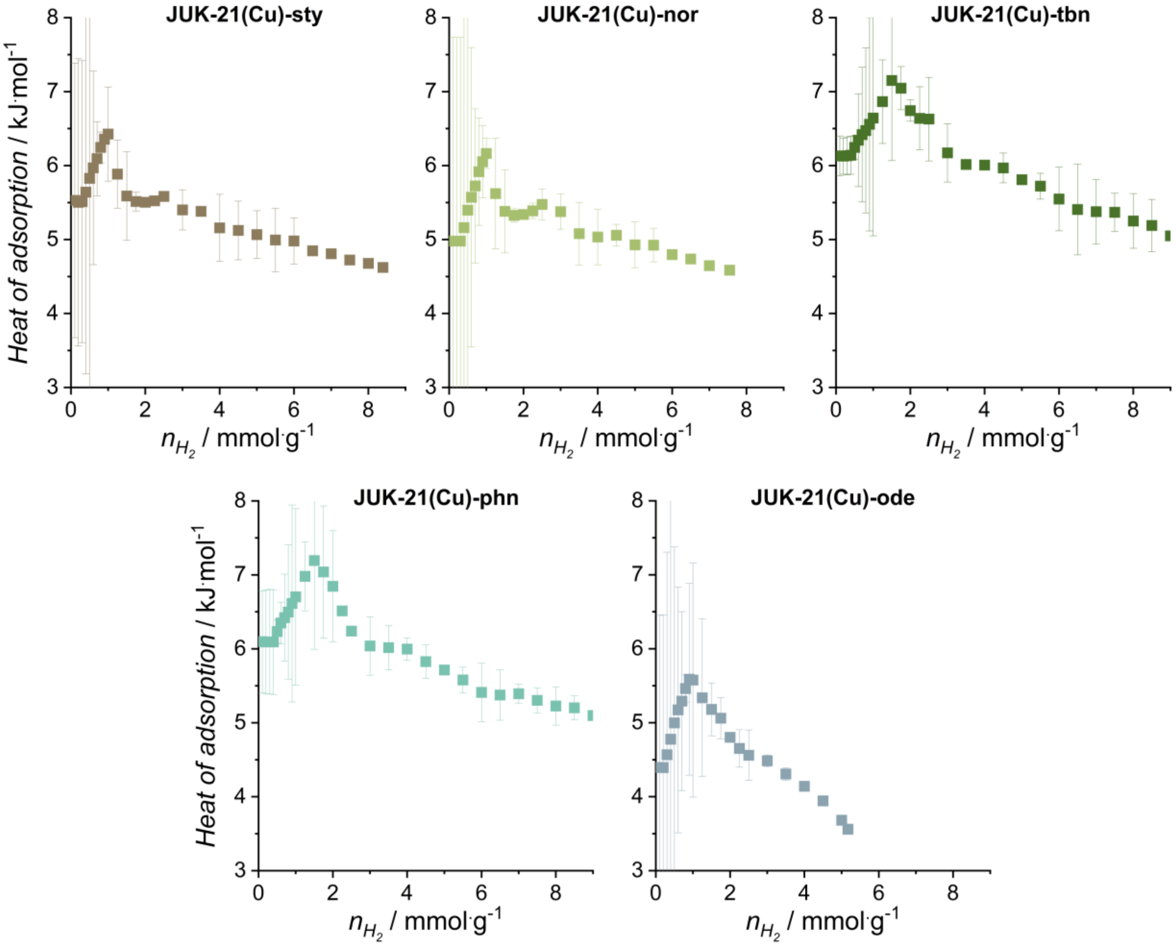

Figure S67. Isosteric heat of adsorption calculated from the data in Figure S66 for all tested materials with error bars indicated.

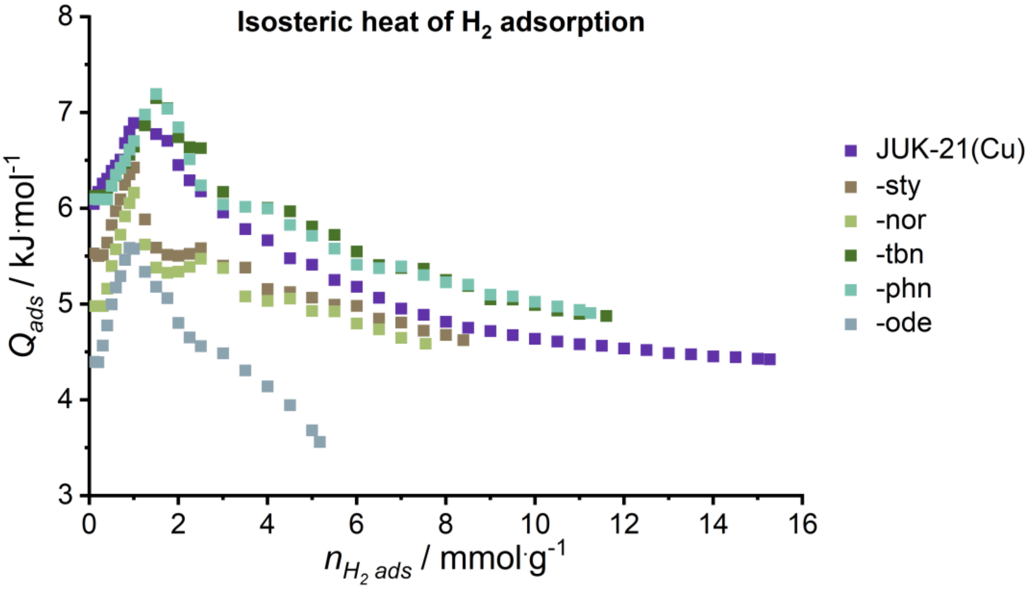

Figure S68. Comparison of isosteric adsorption heats for each of the materials studied. Error bars are omitted for clarity.

For better comparison of the isosteric heat of adsorption patterns between materials, the amount of adsorbed hydrogen (horizontal axis) was converted to the number of moles per mole of Cu_2 unit (Figure S69). Additionally, the graph marks the 2 mol/mol level, which corresponds to the hypothetical stoichiometric chemisorption of two hydrogen molecules on two copper ions. The increase in the heat of hydrogen adsorption for 0-1 mol/mol capacities is, according to our hypothesis, related to a very small effect of changing the affinity of open metal sites to interact with H_2 molecules during the adsorption of the first molecule to the paddlewheel unit, while for higher capacities (above 1 mmol/g) the trend is already decreasing, according to theory (Section S14.6. Low pressure hydrogen adsorption – heat of adsorption).

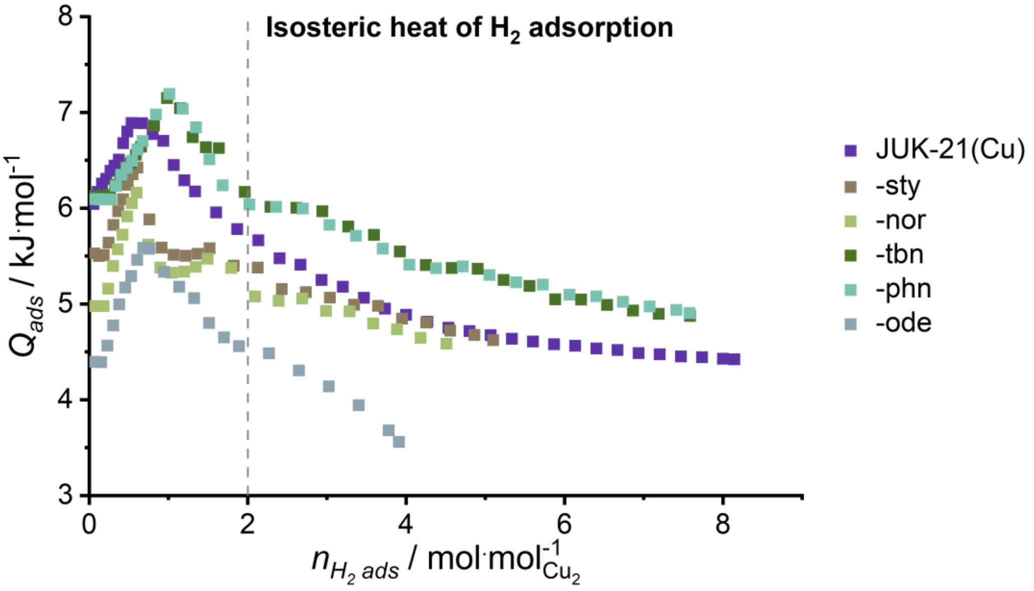

Figure S69. Comparison of isosteric adsorption heats for each of the materials tested, expressed as a function of the amount of adsorbed hydrogen per Cu_2 unit. Error bars are omitted for clarity.

S13. Theoretical studies on iEDDA reaction

Energies (eV) of frontiers orbitals and hardness for dienes (received from calculations according to procedure described in S4.4.) are provided in Table S6.

Table S6. Calculated energy of HOMO and LUMO levels for H_4tztc, JUK-21(Zn) and JUK-21(Cu) dienes and calculated absolute hardness values.

	**H_4tztc**	**JUK-21(Zn)**	**JUK-21(Cu)**
**LUMO (eV)**	-3.6366	-1.857	-1.8416 *
**HOMO (eV)**	-5.1409	-3.28	-2.934
**Hardness (η, eV)**	0.75215	0.7115	0.5462

* – LUMO+3 is taken into account (orbital mainly localized at the tetrazine ring)

The absolute hardness, η, was calculated using following equation:

$$\eta = \frac{LUMO - HOMO}{2}$$

For dienes, the lower hardness the better electrophile as unoccupied orbital has relatively low energy.

Energies (eV) of frontiers orbitals and hardness for dienophiles (received from calculations according to procedure described in S4.4.) are provided in Table S7.

Table S7. Calculated energy of HOMO and LUMO levels for sty, nor, tbn, phn and ode dienophiles and calculated absolute hardness values.

	**sty**	**nor**	**tbn**	**phn**	**ode**
**LUMO (eV)**	-1.4307	-0.2951	-0.1009	-0.1203	-0.2627
**HOMO (eV)**	-5.0523	-5.0773	-4.602	-4.5332	-5.5732
**Hardness (η, eV)**	1.8108	2.3911	2.25055	2.20645	2.65525

For dienophiles, the lower hardness the better donor as occupied orbital has relatively high energy.

Figure S70 and Figure **S71** illustrate electrophilic and nucleophilic Fukui functions, respectively, for Dq = ±0.1e, isovalue 4×10^{-5}. Yellow parts correspond to donating area, while blue parts correspond to accepting area.

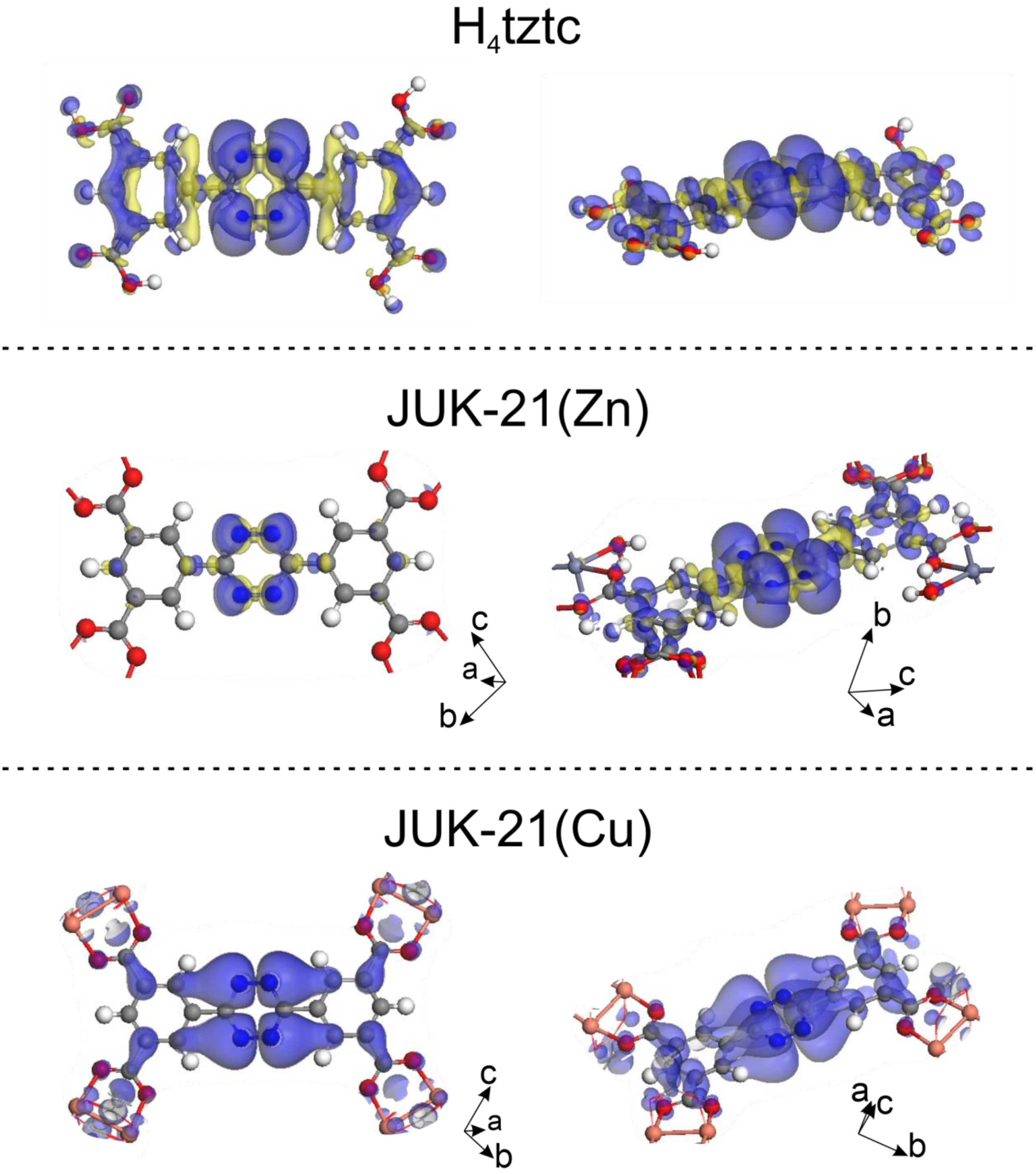

Figure S70. Figures of electrophilic isosurfaces of the Fukui function (shown in blue) for dienes, which correspond to LUMO orbitals (for JUK-21(Cu): LUMO+3). Projections in two directions are presented; for polymeric materials (MOFs), the direction of projection in the triclinic system is indicated.

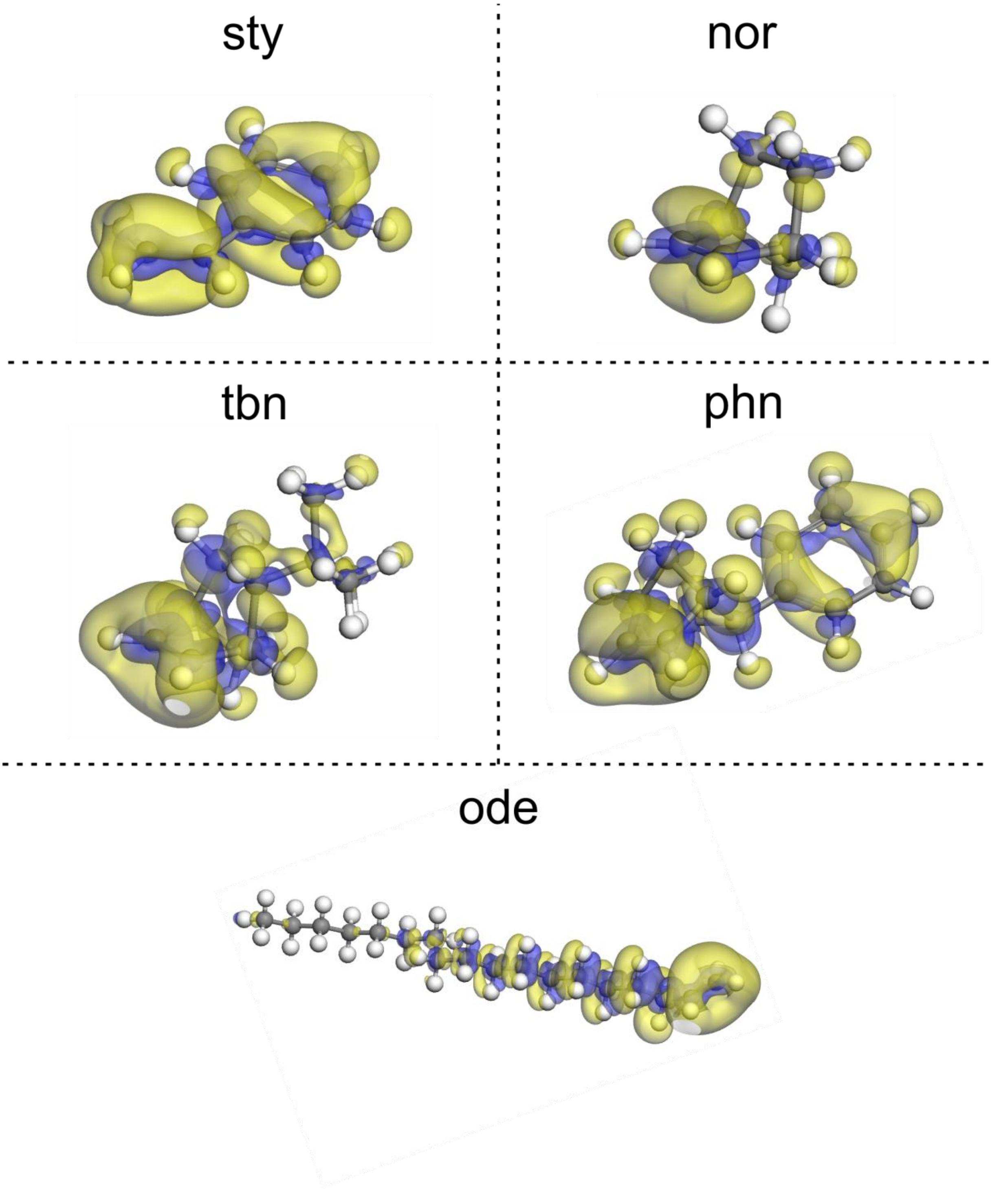

Figure S71. Figures of nucleophilic isosurfaces of the Fukui function (shown in yellow) for dienophiles, which correspond to HOMO orbitals.

S14. Nitrogen and hydrogen adsorption isotherms simulations

S14.1. Structures models and force field optimization

Monte Carlo simulations were conducted for all structures within a 2×2×2 unit cell simulation box, applying periodic boundary conditions.[25] During these calculations, the crystal lattice was considered rigid. Lennard-Jones (LJ) parameters for the frameworks were taken from the DREIDING[26] force field for all the atoms, except for copper, which was taken from the UFF[27] (Table S8). Point charges for the framework were obtained using the EQeq method.[28] Coulombic interactions were calculated with the Ewald summation method with a relative precision of 10^{-6}.

Table S8. Lennard-Jones parameters for the framework atoms.

Framework atoms	ε/k_B [K]	σ[Å]
**Cu**	2.5161	3.1137
**N**	38.9492	3.2626
**C**	47.8586	3.4730
**O**	48.1581	3.0332
**H**	7.6489	2.8464

Adsorption isotherms were calculated using the Grand Canonical Monte Carlo (GCMC) method, maintaining constant chemical potential, volume, and temperature.[29] Pressure was linked to chemical potential via fugacity, following the Peng-Robinson equation of state. Absolute adsorption was converted to excess adsorption using Frenkel et al.'s equation:[30]

$$\theta_{excess} = \theta_{absolute} - \frac{pV}{zRT}$$

where p, V, and T represent the system's pressure, volume, and temperature, R is the gas constant, and z is the gas compressibility. Each adsorption isotherm data point was obtained through $5 \cdot 10^4$ initialization cycles followed by $5 \cdot 10^5$ production cycles, incorporating equally probable trial moves such as translation, rotation, swap, and reinsertion. All simulations were executed using the RASPA code.[31,32]

The TraPPE force field was used to describe the hydrogen molecule[33], while nitrogen was modeled using the approach and LJ parameters developed by Martín-Calvo et al.[34] To account for nitrogen's and hydrogen's quadrupole moments, negative/positive charges were assigned to the nitrogen/hydrogen atoms, with a double-positive/double-negative charge placed at the center of mass (pseudo-atom M), ensuring a net zero molecular charge.[35] These models and LJ parameter sets have been extensively validated.[36] Further details of the models are provided in Table S9.

Table S9. Models of molecules used for the Monte Carlo simulation.

**HYDROGEN**			
**Nonbonded interactions***			
**(pseudo)atom**	ε/k_B [K]	σ [Å]	q [e^-]
**H**	-	-	0.468
**M**	36.7	2.958	-0.936
**Bond lengths**			
**r_{H-H} [Å]**		r_{H-M} [Å]	
**0.74**		0.37	
**Bond angles**			
**Θ_{H-M-H} [deg]**			
**180**			
**NITROGEN**			
**Nonbonded interactions**			
**(pseudo)atom**	ε/k_B [K]	σ [Å]	q [e^-]
**N**	38.298	3.306	-0.40484
**M**	-	-	0.8096
**Bond lengths**			
**$r_{N\equiv N}$ [Å]**		r_{N-M} [Å]	
**1.1**		0.55	
**Bond angles**			
**Θ_{N-M-N} [deg]**			
**180**			

* Unmodified parameters. The developed and used van der Waals interactions are listed in Table S10.

Nonbonded interactions between guest molecules and the framework were modelled using a Lennard-Jones potential:

$$U^{L-J}(r_{ij}) = 4\varepsilon_{ij}\left[\left(\frac{\sigma_{ij}}{r_{ij}}\right)^{12} - \left(\frac{\sigma_{ij}}{r_{ij}}\right)^{6}\right] + \frac{q_i q_j}{4\pi\varepsilon_0 r_{ij}}$$

where r_{ij} is distance between *i* and *j* atoms. Effective potentials were cut and shifted at a cut-off distance of 12 Å. For each JUK-20-X framework atom and adsorbates (pseudo)atoms ε and σ values from Table S8 and Table **S9** were used, which were mixed using Lorentz-Berthelot rules:

$$\varepsilon_{ij} = \sqrt{\varepsilon_i \cdot \varepsilon_j}$$

$$\sigma_{ij} = \frac{\sigma_i + \sigma_j}{2}$$

In addition to nonbonded interactions, intramolecular bonded interactions were incorporated:

1) Harmonic bonding potential:

$$U^{bond}(r_{ij}) = \frac{1}{2}(r_{ij} - r_{ij}^0)^2$$

2) Harmonic bending potential:

$$U^{bend}(\theta_{ijk}) = \frac{1}{2}(\theta_{ijk} - \theta_{ijk}^0)^2$$

3) Torsional potential using the TraPPE cosine series:

$$U^{torsion}(\phi_{ijkl}) = p_0 + p_1[1 + \cos\phi_{ijkl}] + p_2[1 - \cos 2\phi_{ijkl}] + p_3[1 + \cos 3\phi_{ijkl}]$$

where r_{ij}/r_{ij}^0 represent the actual and equilibrium bond lengths, $\theta_{ijk}/\theta_{ijk}^0$ are the actual and equilibrium bond angles, and ϕ_{ijkl} denotes the dihedral angle.

Considering the underestimation of the interactions for hydrogen adsorption, the interactions between (pseudo)atoms and atoms of the JUK-21-X frameworks were appropriately modified using the Lorentz-Berthelot mixing rules. The modified parameters are summarized in Table S10.

Table S10. Modified Lorentz-Berthelot mixing rules for hydrogen adsorption.

**Hydrogen (pseudo)atom**	**JUK-21(Cu)-x atoms**	**ε/k_B [K]**	**σ[Å]**
**M**	C	41.9085	3.3865
**M**	H	16.7546	3.0732
**M**	O	42.0405	3.1666
**M**	N	37.8079	3.2813
**M**	Cu	116.666	3.267

S14.2. Structural properties of computational models – helium void fractions, pore volumes and pore size distributions

The Widom particle-insertion method[37] was used to determine heats of adsorption and helium void fractions (HVF). Pore volumes were derived from HVF and framework density, with system characteristics summarized in Table S11.

Table S11. Characteristics of the structures used in this work, where PV is pore volume and HVF is helium void fraction. All parameters are given for 2 × 2 × 2 supercells.

	**JUK-21(Cu)**	**-sty**	**-nor**	**-tbn**	**-phn**	**-ode**
**Cell lengths (Å) (a = b = c)**	33.1206	33.1206	33.1206	33.1206	33.1206	33.1206
**Cell angles (°) (α = β = γ)**	68.2458	68.2458	68.2458	68.2458	68.2458	68.2458
**Framework density ($kg \cdot m^{-3}$)**	704.4371	802.3252	789.1316	865.0992	889.6288	998.2322
**PV ($cm^3 \cdot g^{-1}$)**	1.1095	0.8221	0.8374	0.7020	0.6519	0.4027
**HVF**	0.7816	0.6596	0.6608	0.6073	0.5799	0.4020

S14.3. High pressure hydrogen adsorption – isotherm simulation

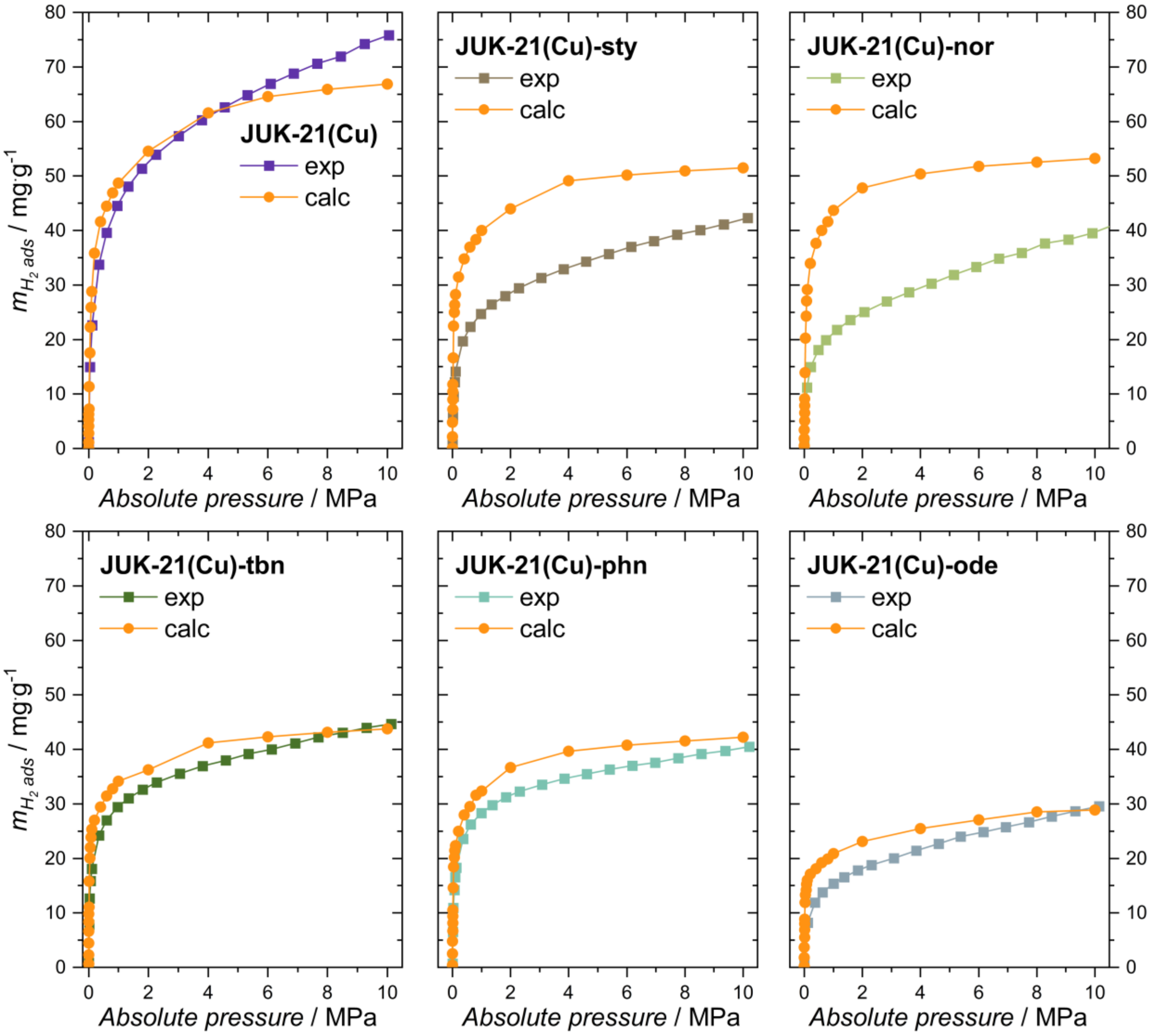

Figure S72. Hydrogen adsorption isotherms (desorption omitted for clarity) in the high-pressure range registered experimentally (exp, total isotherms, Figure S60) and calculated theoretically using GCMC simulations (calc) for a series of JUK-21(Cu)-x materials.

S14.4. High pressure hydrogen adsorption – average occupation profiles

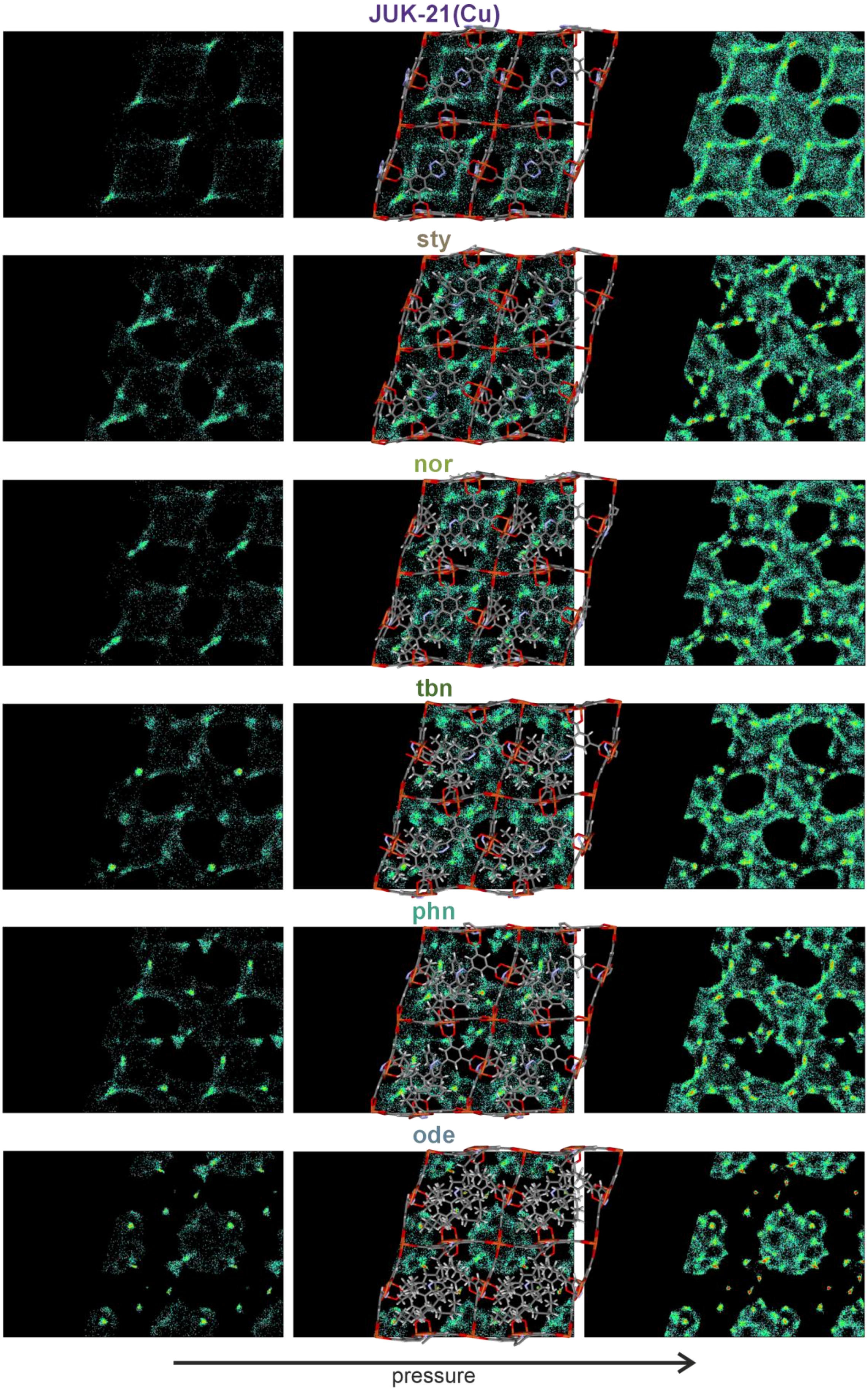

Figure S73. Average occupation profiles for H_2 adsorption by JUK-21(Cu)-x materials at three pressures (from left): 6 kPa, 60 kPa, 6000 kPa, in the projection along [100] (triclinic cell).

S14.5. High pressure hydrogen adsorption – radial distribution function

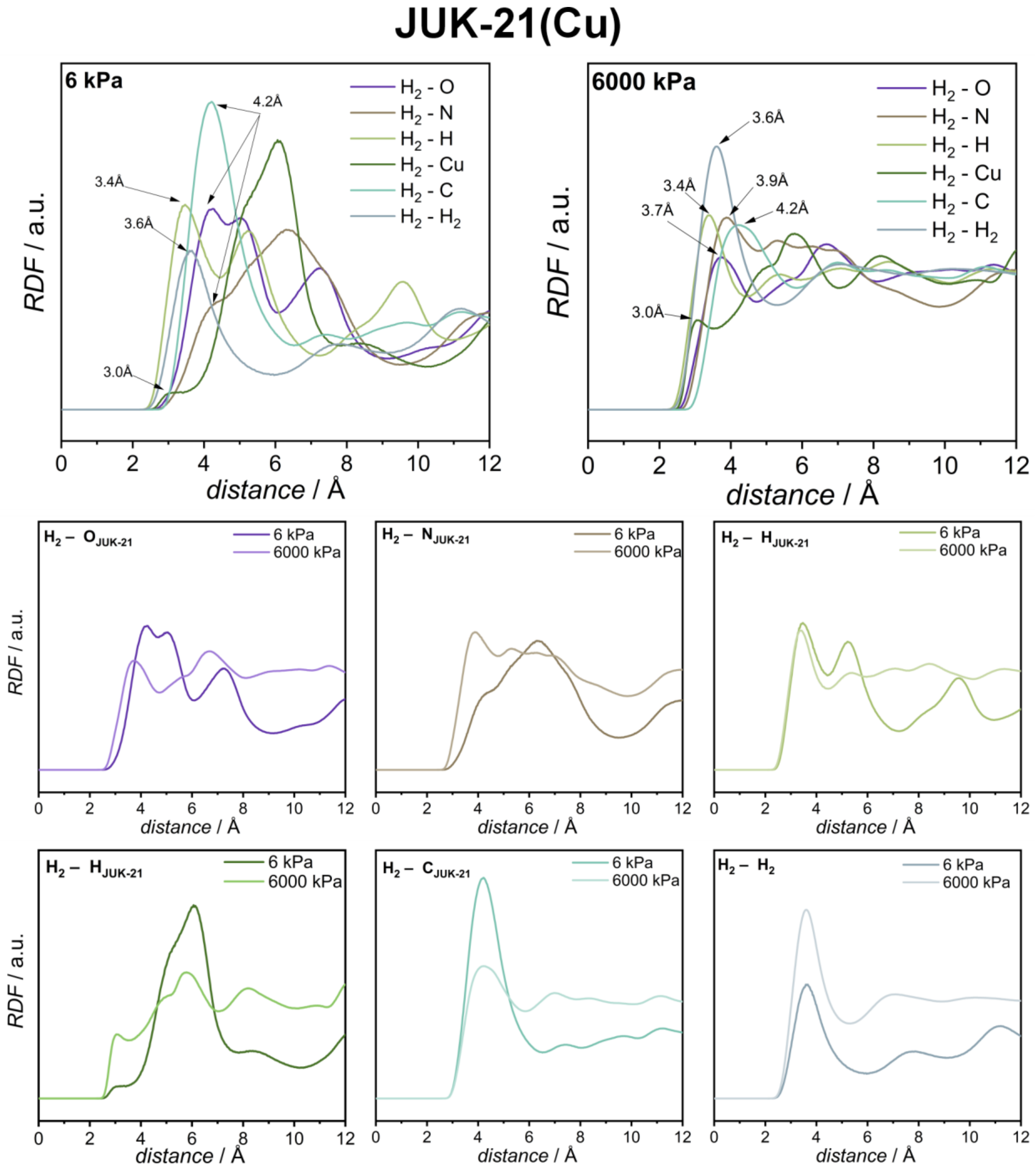

Figure S74. Radial distribution function (RDF) determined at two pressures (6 and 6000 kPa) of hydrogen adsorption by JUK-21(Cu) material. The first two panels (first line) represent the distances of adsorbed hydrogen molecules from each of the types of atoms building the MOF skeleton (or to another H_2 molecule), while the remaining figures (second and third line) show RDF profiles separately for each H_2-atom/H_2 interaction.

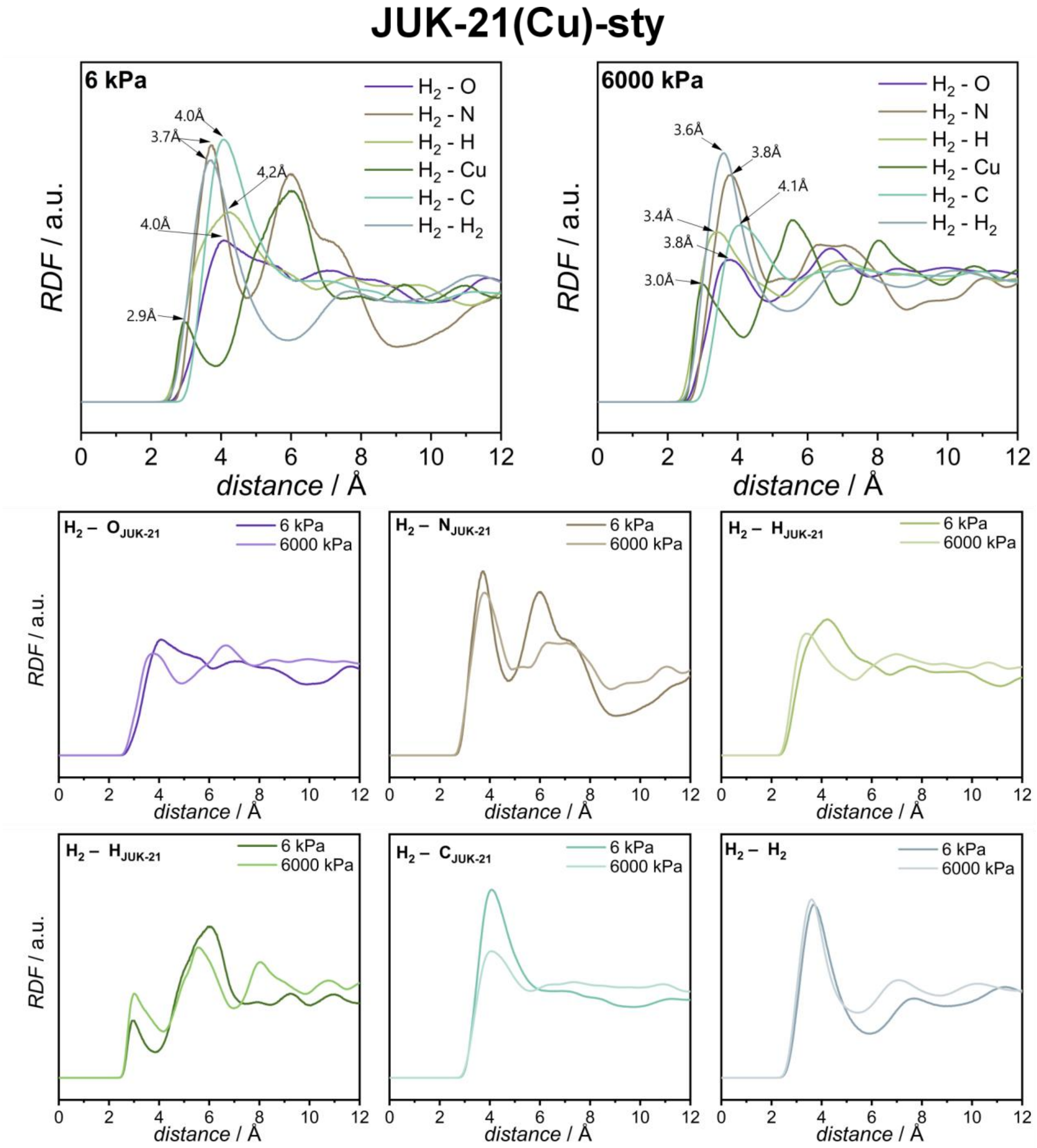

Figure S75. Radial distribution function (RDF) determined at two pressures (6 and 6000 kPa) of hydrogen adsorption by JUK-21(Cu)-sty material. The first two panels (first line) represent the distances of adsorbed hydrogen molecules from each of the types of atoms building the MOF skeleton (or to another H_2 molecule), while the remaining figures (second and third line) show RDF profiles separately for each H_2-atom/H_2 interaction.

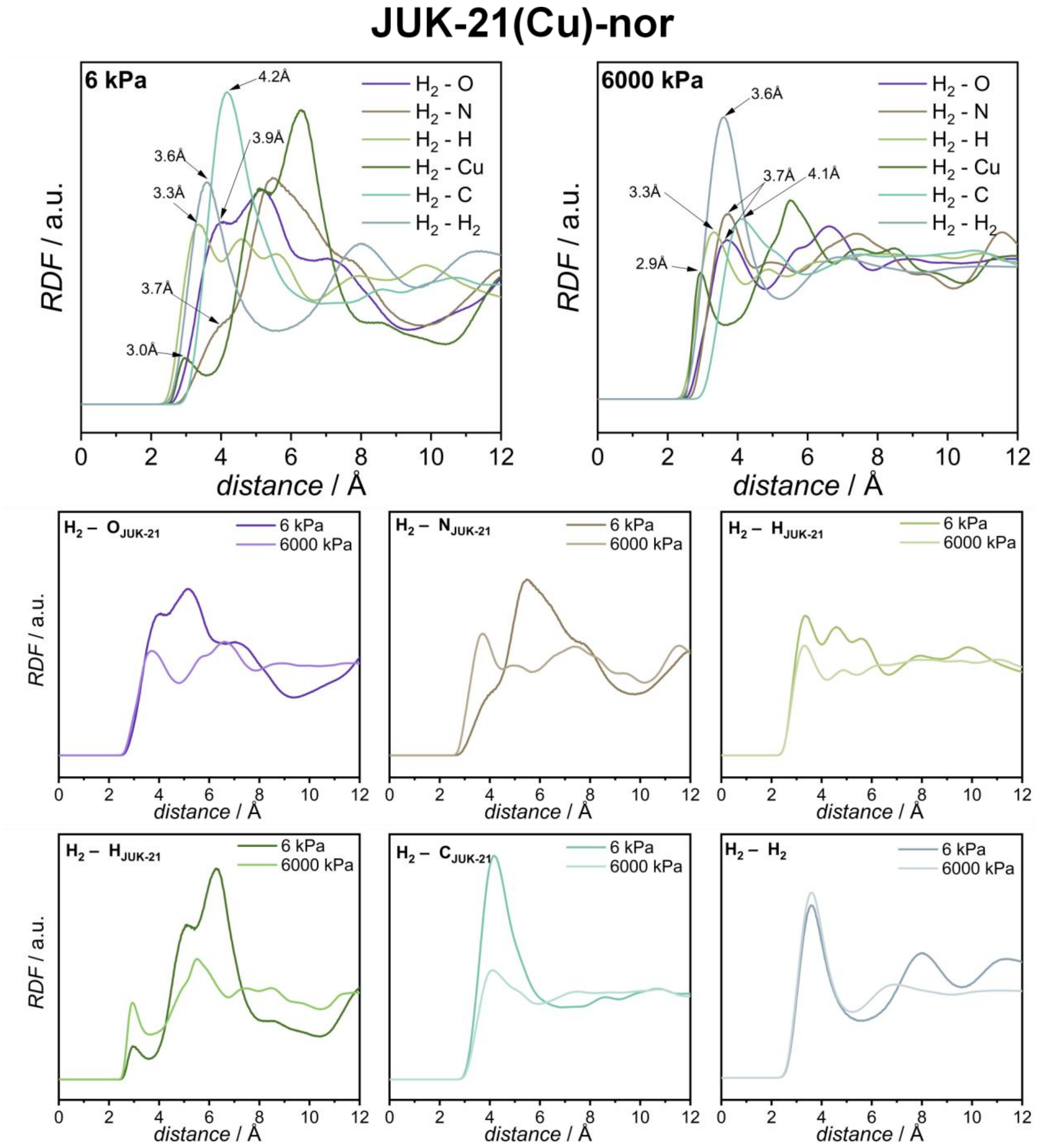

Figure S76. Radial distribution function (RDF) determined at two pressures (6 and 6000 kPa) of hydrogen adsorption by JUK-21(Cu)-nor material. The first two panels (first line) represent the distances of adsorbed hydrogen molecules from each of the types of atoms building the MOF skeleton (or to another H_2 molecule), while the remaining figures (second and third line) show RDF profiles separately for each H_2-atom/H_2 interaction.

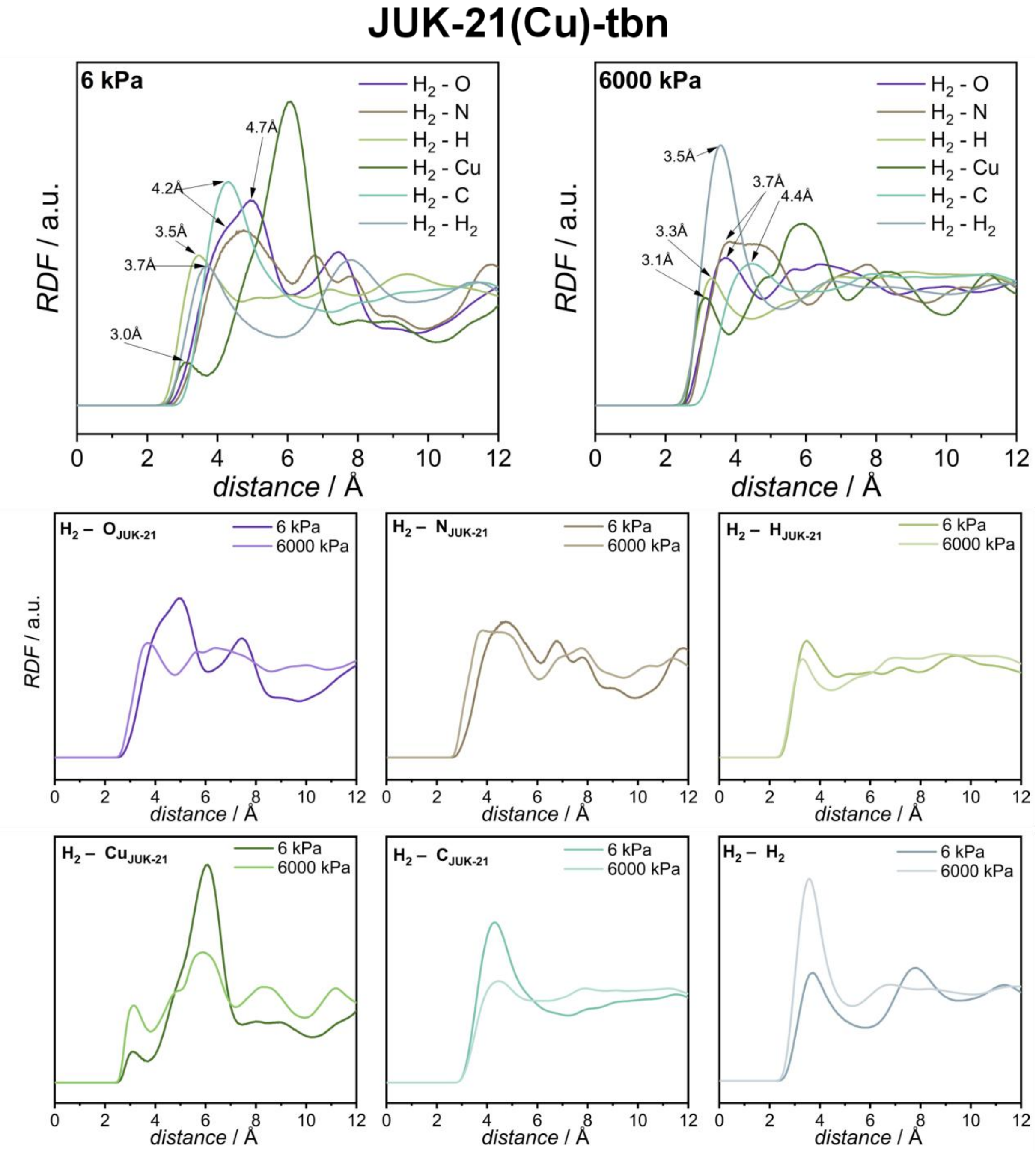

Figure S77. Radial distribution function (RDF) determined at two pressures (6 and 6000 kPa) of hydrogen adsorption by JUK-21(Cu)-tbn material. The first two panels (first line) represent the distances of adsorbed hydrogen molecules from each of the types of atoms building the MOF skeleton (or to another H_2 molecule), while the remaining figures (second and third line) show RDF profiles separately for each H_2-atom/H_2 interaction.

JUK-21(Cu)-phn

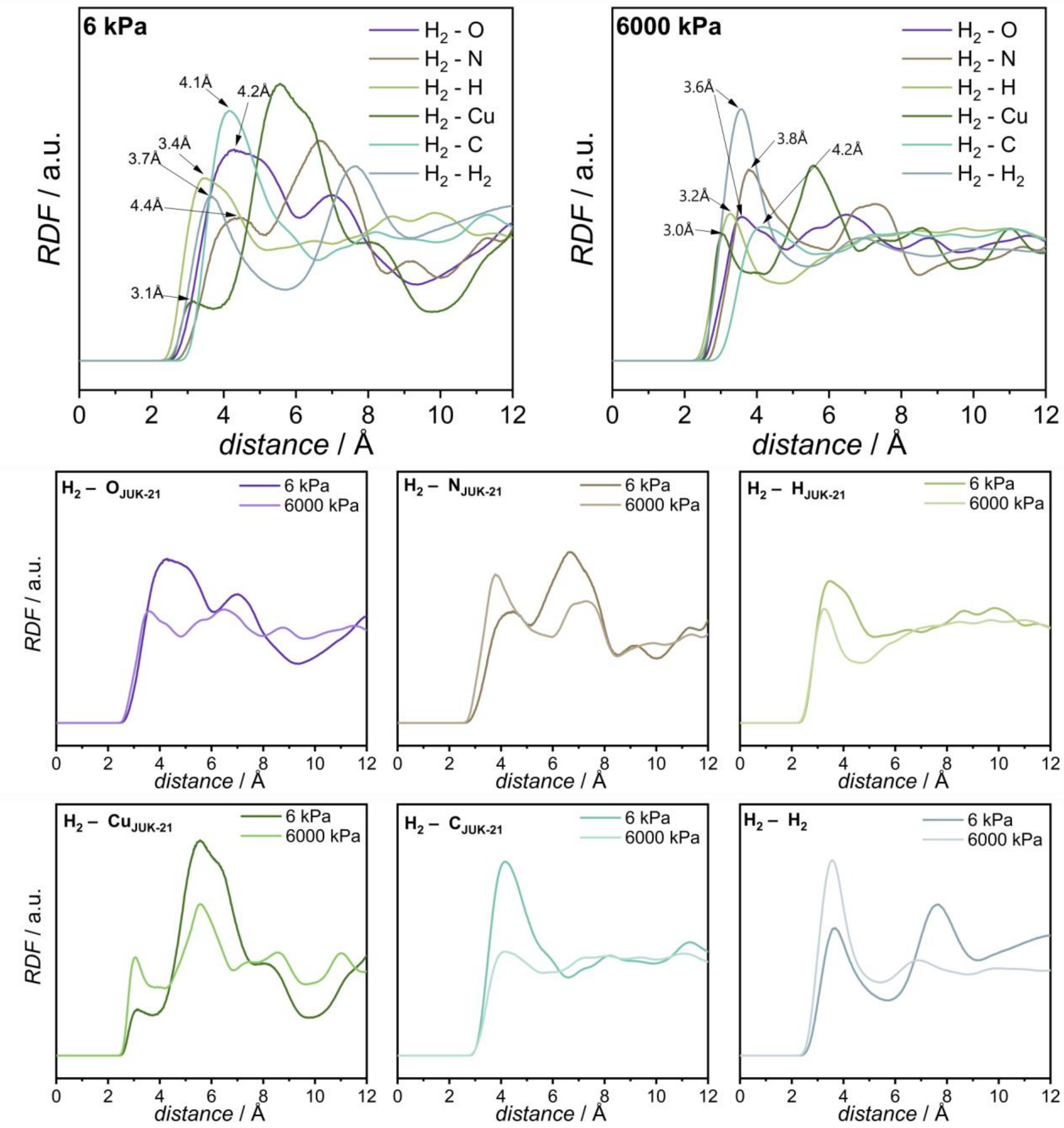

Figure S78. Radial distribution function (RDF) determined at two pressures (6 and 6000 kPa) of hydrogen adsorption by JUK-21(Cu)-phn material. The first two panels (first line) represent the distances of adsorbed hydrogen molecules from each of the types of atoms building the MOF skeleton (or to another H_2 molecule), while the remaining figures (second and third line) show RDF profiles separately for each H_2-atom/H_2 interaction.

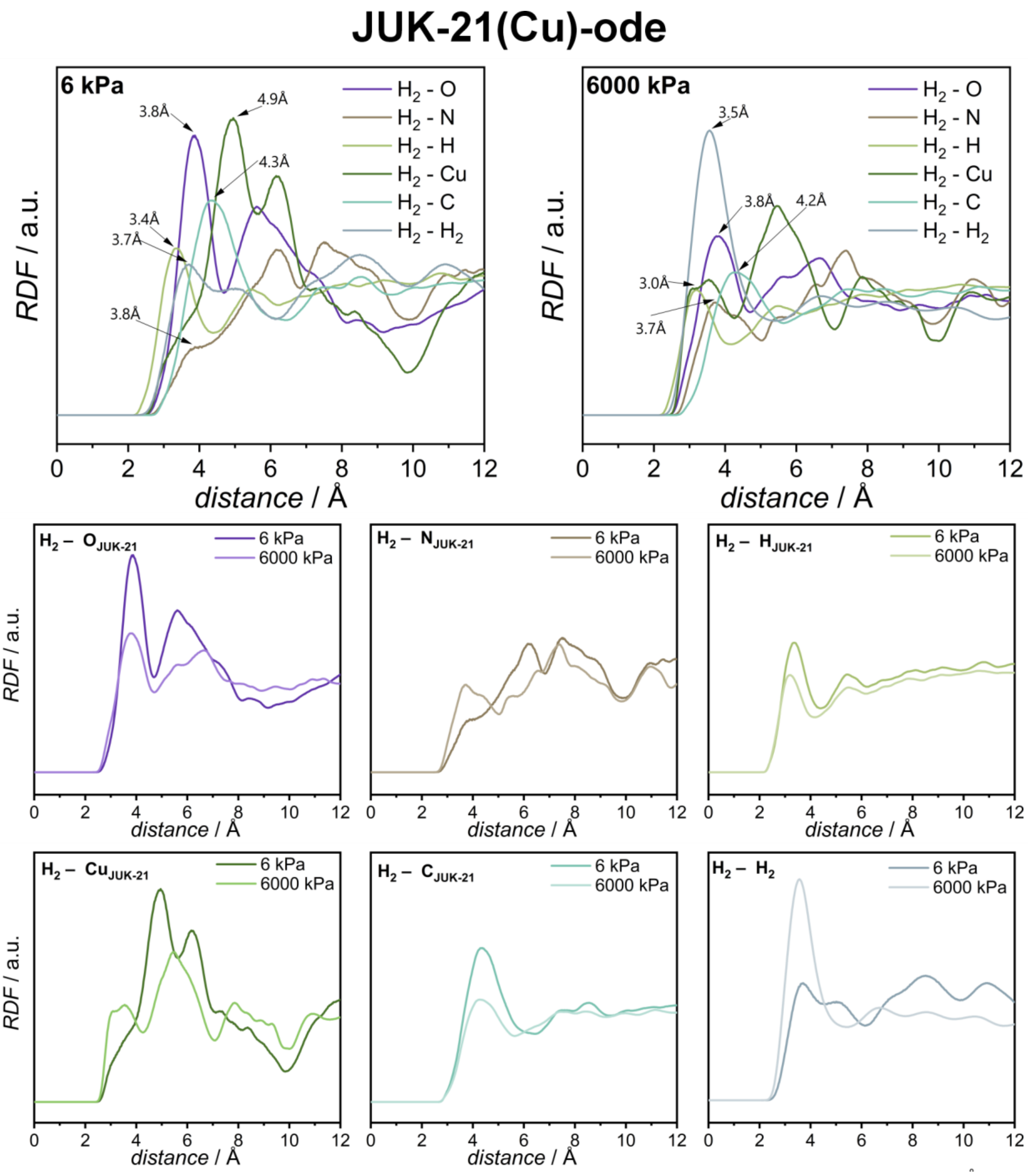

Figure S79. Radial distribution function (RDF) determined at two pressures (6 and 6000 kPa) of hydrogen adsorption by JUK-21(Cu)-ode material. The first two panels (first line) represent the distances of adsorbed hydrogen molecules from each of the types of atoms building the MOF skeleton (or to another H_2 molecule), while the remaining figures (second and third line) show RDF profiles separately for each H_2-atom/H_2 interaction.

S14.6. Low pressure hydrogen adsorption – heat of adsorption

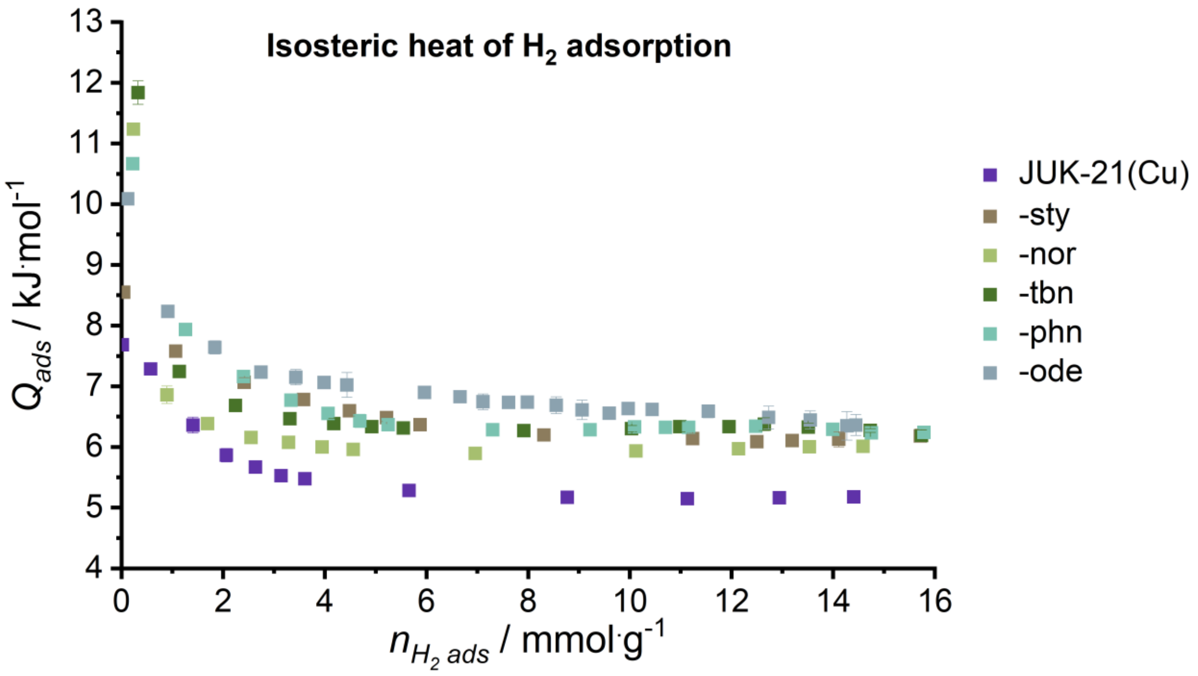

Figure S80. Comparison of isosteric adsorption heats for each of the materials studied, calculated using GCMC simulation.

In order to qualitatively compare experimental (Figure S68) and theoretically calculated (Figure S80) data on the heat of hydrogen adsorption by a series of JUK-21(Cu)-x materials, three ranges were investigated. The range of adsorption of the first portions of hydrogen (0-1 mmol/g), which is subject to excessive experimental error (cf. error bars in Figure S67), was instead investigated theoretically with GCMC simulations and using the Widom particle-insertion method. For the 1-2 mmol/g range, a maximum value was taken for the experimental data, similarly to the theoretical data. From the further isosteric heat of adsorption, the capacity that can be compared for the entire series of materials, i.e. 5 mmol/g, was selected. These data readings were collected in Table S12 and are presented as two graphical comparisons - in Figure S81 a summary comparison of the entire series of materials in the experimental and theoretical data, and in Figure S82 separately for each material.

Table S12. The hydrogen heat of adsorption values for the material series under study: experimental (exp) as read from Figure S68 (maximum value in the range of 1-2 mmol/g and value at the point of 5 mmol/g), theoretical (calc) as read from Figure S71 (maximum value, i.e., initial value (range 0-1 mmol/g), maximum value in the range of 1-2 mmol/g and value at the point of 5 mmol/g), and theoretical value calculated using the Widom particle-insertion method. All values are expressed in kJ/mol.

	Q_{exp} ^{max} @ 1-2 mmol/g	Q_{exp} @ 5 mmol/g	Q_{calc} ^{max} @ 0-1 mmol/g	Q_{calc} ^{max} @ 1-2 mmol/g	Q_{calc} @ 5 mmol/g	Q_{Widom}
**JUK-21(Cu)**	6.89	5.41	7.69	6.83	5.56	6.45
**JUK-21(Cu) -sty**	6.43	5.07	8.55	7.58	6.50	7.33
**JUK-21(Cu) -nor**	6.16	4.93	11.24	6.86	5.96	10.11
**JUK-21(Cu) -tbn**	7.15	5.81	11.84	7.35	6.34	11.08
**JUK-21(Cu) -phn**	7.19	5.71	10.67	7.94	6.40	9.65
**JUK-21(Cu) -ode**	5.59	3.68	10.09	8.24	6.96	8.89

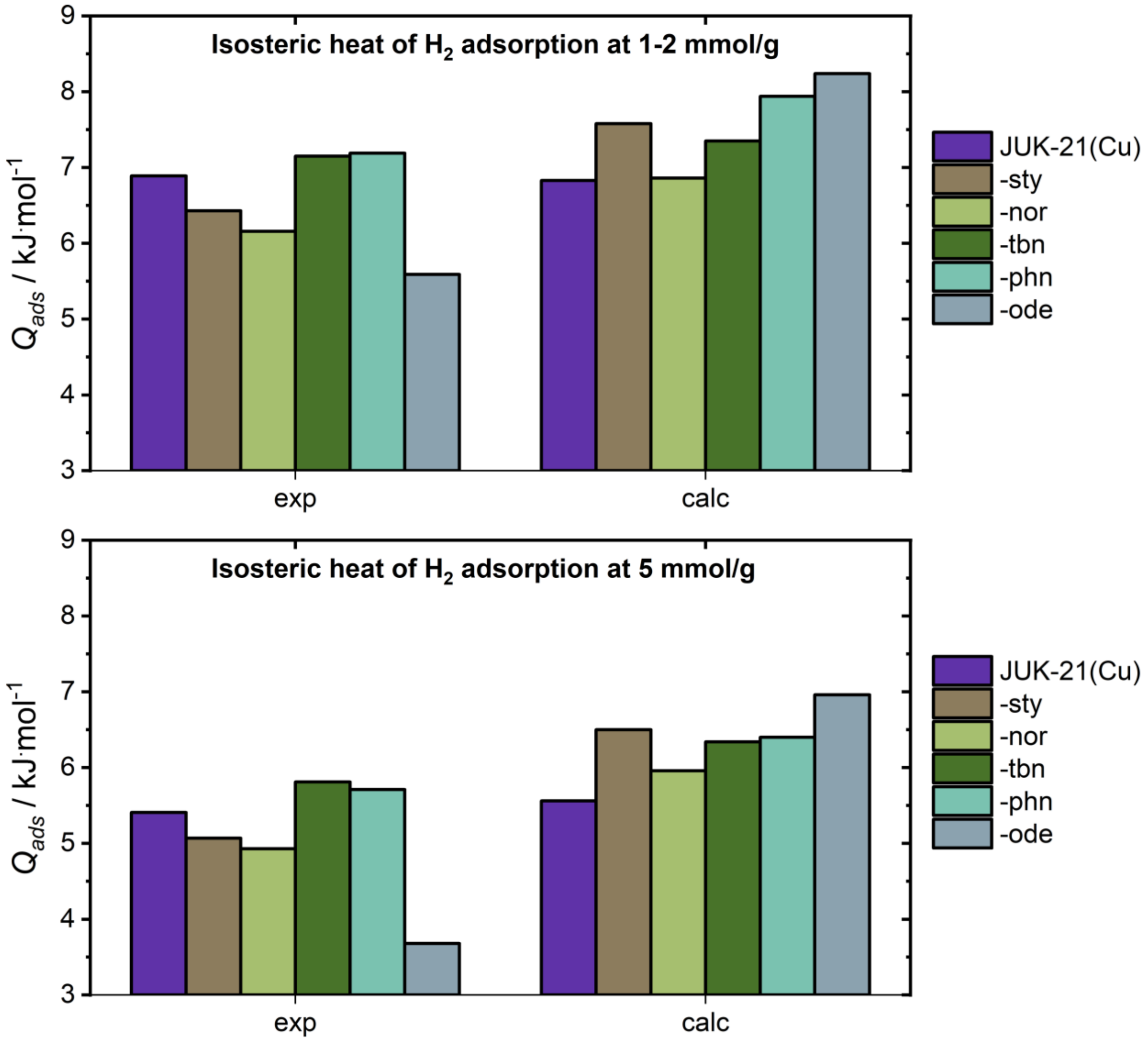

Figure S81. Comparison of experimental and calculated heat of adsorption values for the JUK-21(Cu)-x series, investigated in the 1-2 mmol/g range (top) and at the 5 mmol/g point (bottom). Values extracted from Table S12.

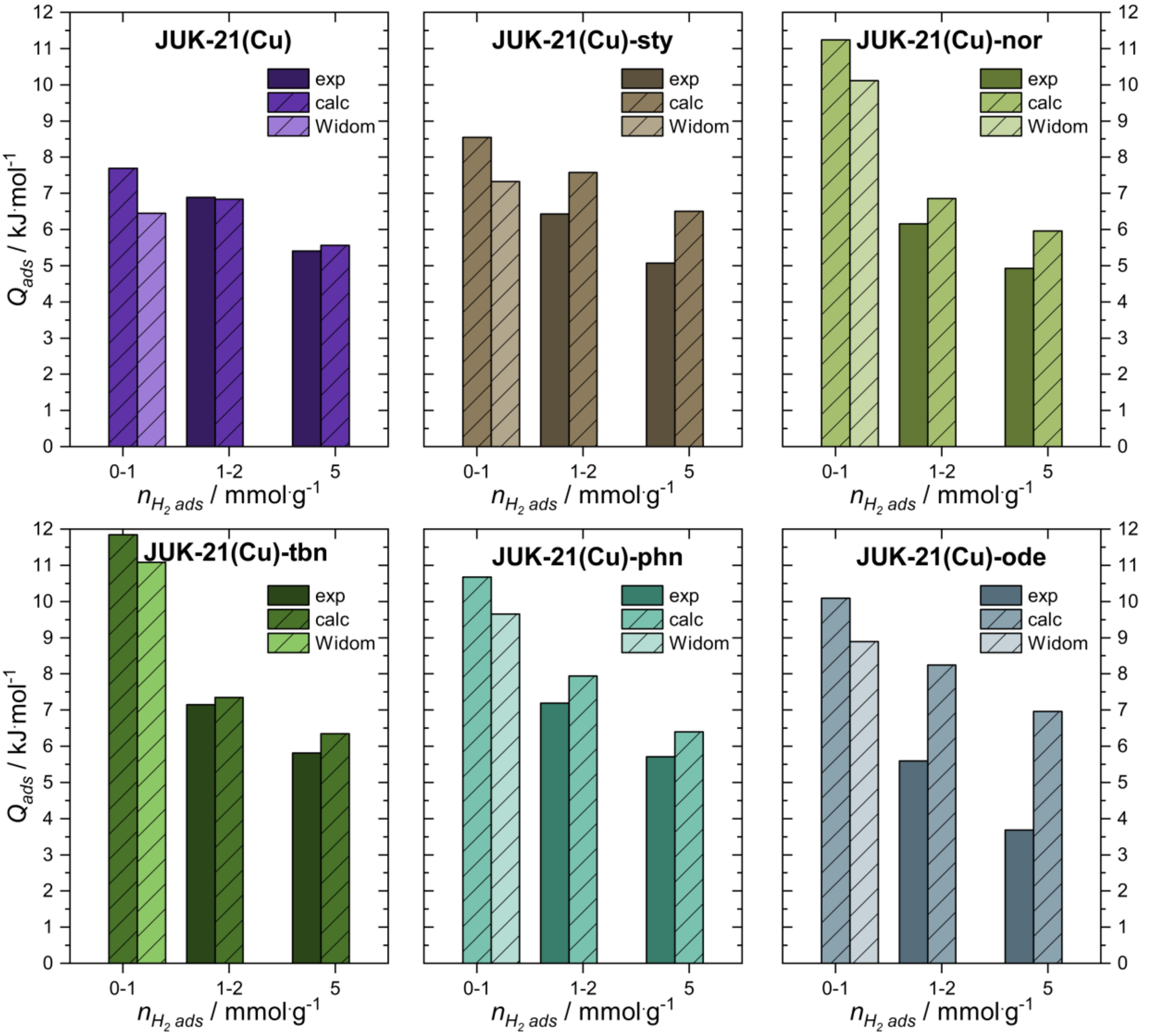

Figure S82. Comparison of experimental and calculated heat of adsorption values for the JUK-21(Cu)-x series, presented for each material separately, investigated in the 0-1 mmol/g range, 1-2 mmol/g range and at the 5 mmol/g point. Values extracted from Table S12.

S14.7. Low pressure nitrogen adsorption – isotherm simulation

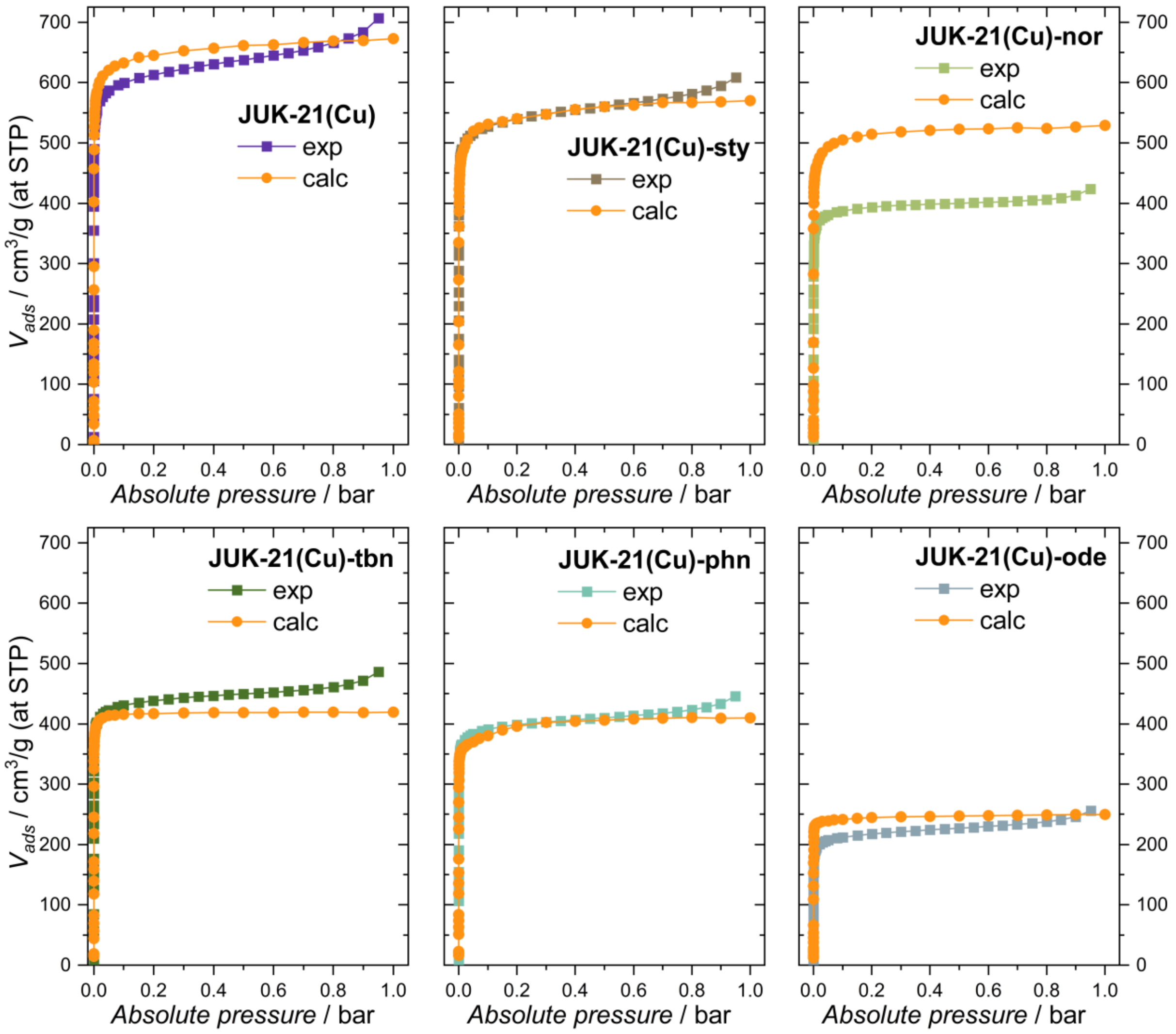

Figure S83. Nitrogen adsorption isotherms (desorption omitted for clarity) registered experimentally (exp, Figure S57) and calculated theoretically using GCMC simulations (calc) for a series of JUK-21(Cu)-x materials.

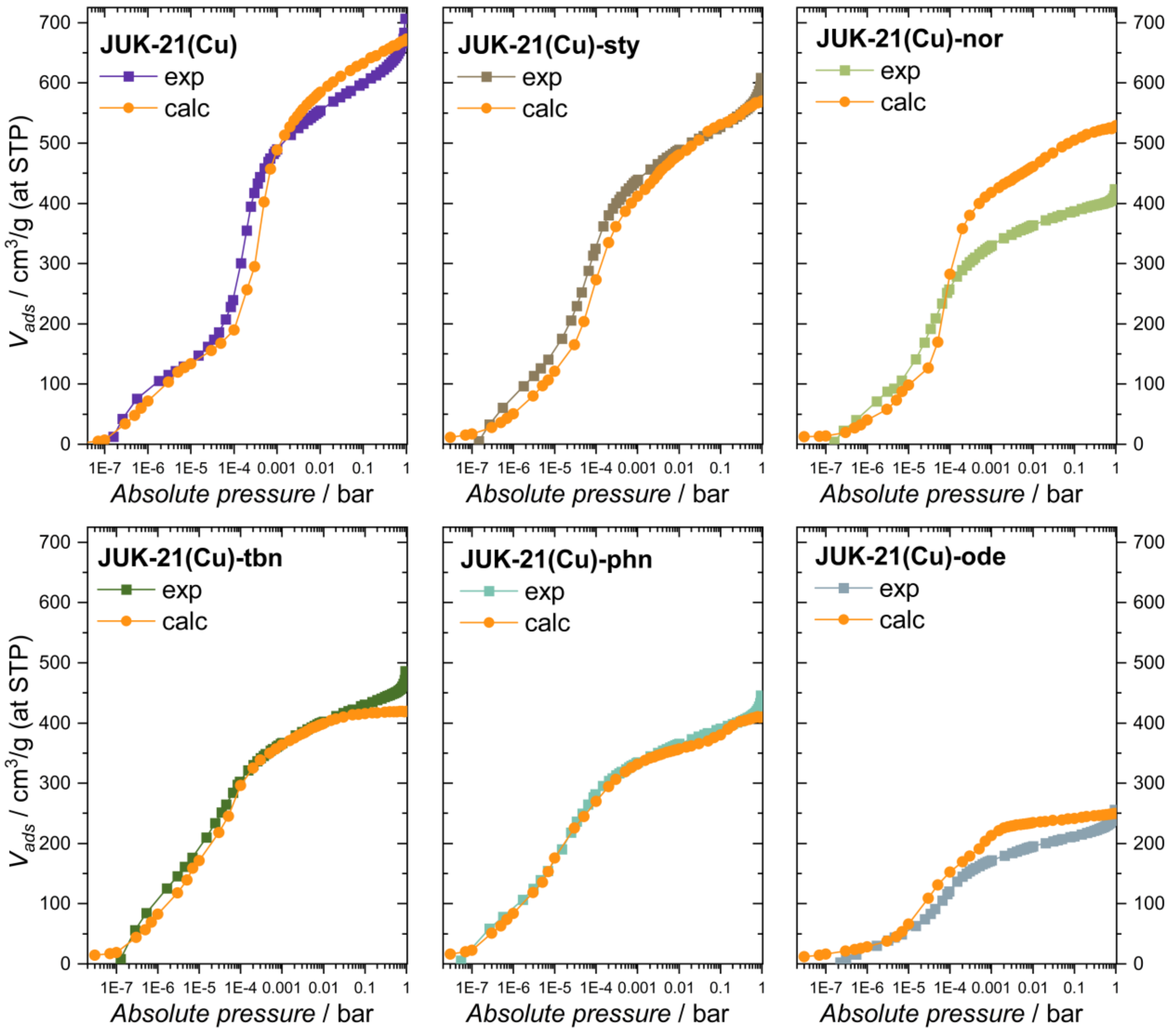

Figure S84. Nitrogen adsorption isotherms (desorption omitted for clarity) registered experimentally (exp, Figure S57) and calculated theoretically using GCMC simulations (calc) for a series of JUK-21(Cu)-x materials, expressed in logarithmic scale of pressure.

S15. References

[1] P. Leber, K. Kidder, D. Viray, E. Dietrich-Peterson, Y. Fang, A. Davis, *J. Phys. Org. Chem.* **2018**, *31*, e3888.

[2] A. N. Artemov, E. V. Sazonova, M. V. Revin, K. V. Rybkin, M. A. Lazarev, V. I. Faerman, *Russ. Chem. Bull.* **2011**, *60*, 2103–2106.

[3] T. Francart, A. van Wieringen, J. Wouters, *J. Neurosci. Methods* **2008**, *172*, 283–293.

[4] G. M. Sheldrick, *Acta Crystallogr. A* **2008**, *64*, 112–122.

[5] O. V. Dolomanov, L. J. Bourhis, R. J. Gildea, J. a. K. Howard, H. Puschmann, *J. Appl. Crystallogr.* **2009**, *42*, 339–341.

[6] J. W. M. Osterrieth, J. Rampersad, D. Madden, N. Rampal, L. Skoric, B. Connolly, M. D. Allendorf, V. Stavila, J. L. Snider, R. Ameloot, J. Marreiros, C. Ania, D. Azevedo, E. Vilarrasa-Garcia, B. F. Santos, X.-H. Bu, Z. Chang, H. Bunzen, N. R. Champness, S. L. Griffin, B. Chen, R.-B. Lin, B. Coasne, S. Cohen, J. C. Moreton, Y. J. Colón, L. Chen, R. Clowes, F.-X. Coudert, Y. Cui, B. Hou, D. M. D'Alessandro, P. W. Doheny, M. Dincă, C. Sun, C. Doonan, M. T. Huxley, J. D. Evans, P. Falcaro, R. Ricco, O. Farha, K. B. Idrees, T. Islamoglu, P. Feng, H. Yang, R. S. Forgan, D. Bara, S. Furukawa, E. Sanchez, J. Gascon, S. Telalović, S. K. Ghosh, S. Mukherjee, M. R. Hill, M. M. Sadiq, P. Horcajada, P. Salcedo-Abraira, K. Kaneko, R. Kukobat, J. Kenvin, S. Keskin, S. Kitagawa, K. Otake, R. P. Lively, S. J. A. DeWitt, P. Llewellyn, B. V. Lotsch, S. T. Emmerling, A. M. Pütz, C. Martí-Gastaldo, N. M. Padial, J. García-Martínez, N. Linares, D. Maspoch, J. A. Suárez del Pino, P. Moghadam, R. Oktavian, R. E. Morris, P. S. Wheatley, J. Navarro, C. Petit, D. Danaci, M. J. Rosseinsky, A. P. Katsoulidis, M. Schröder, X. Han, S. Yang, C. Serre, G. Mouchaham, D. S. Sholl, R. Thyagarajan, D. Siderius, R. Q. Snurr, R. B. Goncalves, S. Telfer, S. J. Lee, V. P. Ting, J. L. Rowlandson, T. Uemura, T. Iiyuka, M. A. van der Veen, D. Rega, V. Van Speybroeck, S. M. J. Rogge, A. Lamaire, K. S. Walton, L. W. Bingel, S. Wuttke, J. Andreo, O. Yaghi, B. Zhang, C. T. Yavuz, T. S. Nguyen, F. Zamora, C. Montoro, H. Zhou, A. Kirchon, D. Fairen-Jimenez, *Adv. Mater.* **2022**, *34*, 2201502.

[7] J. Rouquerol, P. Llewellyn, F. Rouquerol, in *Stud. Surf. Sci. Catal.*, Elsevier B. V., **2007**, pp. 49–56.

[8] M. Patel, A. K. Verma, in *Compr. Heterocycl. Chem. IV* (Eds.: D.S. Black, J. Cossy, C.V. Stevens), Elsevier, Oxford, **2022**, pp. 662–686.

[9] G. M. Sheldrick, *Acta Crystallogr. Sect. Found. Adv.* **2015**, *71*, 3–8.

[10] A. L. Spek, *Acta Crystallogr. Sect. C Struct. Chem.* **2015**, *71*, 9–18.

[11] G. Kresse, J. Furthmüller, *Comput. Mater. Sci.* **1996**, *6*, 15–50.

[12] G. Kresse, J. Hafner, *Phys. Rev. B* **1993**, *48*, 13115–13118.

[13] P. E. Blöchl, *Phys. Rev. B* **1994**, *50*, 17953–17979.

[14] G. Kresse, D. Joubert, *Phys. Rev. B* **1999**, *59*, 1758–1775.

[15] M. Methfessel, A. T. Paxton, *Phys. Rev. B* **1989**, *40*, 3616–3621.

[16] J. P. Perdew, K. Burke, M. Ernzerhof, *Phys. Rev. Lett.* **1996**, *77*, 3865–3868.

[17] J. P. Perdew, K. Burke, M. Ernzerhof, *Phys. Rev. Lett.* **1997**, *78*, 1396–1396.

[18] S. Grimme, *J. Comput. Chem.* **2004**, *25*, 1463–1473.

[19] S. Grimme, *J. Comput. Chem.* **2006**, *27*, 1787–1799.

[20] S. Grimme, *WIREs Comput. Mol. Sci.* **2011**, *1*, 211–228.

[21] H. J. Monkhorst, J. D. Pack, *Phys. Rev. B* **1976**, *13*, 5188–5192.

[22] A. M.-H. Yip, C. K.-H. Lai, K. S.-M. Yiu, K. K.-W. Lo, *Angew. Chem. Int. Ed.* **2022**, *61*, e202116078.

[23] M. P. Suh, H. J. Park, T. K. Prasad, D.-W. Lim, *Chem. Rev.* **2012**, *112*, 782–835.

[24] A. Nuhnen, C. Janiak, *Dalton Trans.* **2020**, *49*, 10295–10307.

[25] D. Frenkel, B. Smit, J. Tobochnik, S. R. McKay, W. Christian, *Comput. Phys.* **1997**, *11*, 351–354.

[26] S. L. Mayo, B. D. Olafson, W. A. Goddard, *J. Phys. Chem.* **1990**, *94*, 8897–8909.

[27] A. K. Rappe, C. J. Casewit, K. S. Colwell, W. A. I. Goddard, W. M. Skiff, *J. Am. Chem. Soc.* **1992**, *114*, 10024–10035.

[28] C. E. Wilmer, K. C. Kim, R. Q. Snurr, *J. Phys. Chem. Lett.* **2012**, *3*, 2506–2511.

[29] D. Frenkel, B. Smit, M. A. Ratner, *Phys. Today* **1997**, *50*, 66.

[30] D. Frenkel, B. Smit, M. A. Ratner, *Phys. Today* **1997**, *50*, 66.

[31] D. Dubbeldam, S. Calero, D. E. Ellis, R. Q. Snurr, *Mol. Simul.* **2016**, *42*, 81–101.

[32] D. Dubbeldam, A. Torres-Knoop, K. S. Walton, *Mol. Simul.* **2013**, *39*, 1253–1292.

[33] B. Chen, J. J. Potoff, J. I. Siepmann, *J. Phys. Chem. B* **2001**, *105*, 3093–3104.

[34] A. Martín-Calvo, E. García-Pérez, A. García-Sánchez, R. Bueno-Pérez, S. Hamad, S. Calero, *Phys. Chem. Chem. Phys.* **2011**, *13*, 11165–11174.

[35] C. S. Murthy, K. Singer, M. L. Klein, I. R. McDonald, *Mol. Phys.* **1980**, *41*, 1387–1399.

[36] J. J. Gutiérrez-Sevillano, J. M. Vicent-Luna, D. Dubbeldam, S. Calero, *J. Phys. Chem. C* **2013**, *117*, 11357–11366.

[37] B. Widom, *J. Chem. Phys.* **1963**, *39*, 2808–2812.
